# Anti-colorectal cancer effect of total minor ginsenosides produced by lactobacilli transformation of major ginsenosides by inducing apoptosis and regulating gut microbiota

**DOI:** 10.3389/fphar.2024.1496346

**Published:** 2025-01-08

**Authors:** Yunjiao Shen, Yansong Gao, Ge Yang, Zijian Zhao, Yujuan Zhao, Lei Gao, Shengyu Li

**Affiliations:** ^1^ Institute of Agro-food Technology, Jilin Academy of Agricultural Sciences (Northeast Agricultural Research Center of China), Changchun, China; ^2^ School of Chinese Materia Medica, State Key Laboratory of Component-based Chinese Medicine, Tianjin University of Traditional Chinese Medicine, Tianjin, China

**Keywords:** ginsenoside, lactic acid bacteria, minor ginsenosides, apoptosis, anti-cancer, gut microbiota

## Abstract

**Objective:**

Minor ginsenosides have demonstrated promising anticancer effects in previous reports. Total minor ginsenosides (TMG) were obtained through the fermentation of major ginsenosides with *Lactiplantibacillus plantarum*, and potential anticancer effects of TMGs on the mouse colon cancer cell line CT26.WT, *in vitro* and *in vivo*, were investigated.

**Materials and Methods:**

We employed the Cell Counting Kit-8 (CCK-8), TdT-mediated dUTP nick end labeling (TUNEL), and Western blot analysis *in vitro* to explore the anti-proliferative and pro-apoptotic functions of TMG in CT26.WT cells. *In vivo*, a xenograft model was established by subcutaneously injecting mice with CT26.WT cells and administering a dose of 100 mg/kg/day TMG to the tumor-bearing mice. The level of apoptosis and expression of various proteins in the tumor tissues were detected by immunohistochemistry and Western blot. High-throughput 16S rRNA sequencing was used to determine the alterations in the gut microbiota.

**Results:**

*In vitro* studies demonstrated that TMG significantly inhibited proliferation and promoted apoptosis in CT26.WT cells. Interestingly, TMG induced apoptosis in CT26.WT cells by affecting the Bax/Bcl-2/caspase-3 pathway. Furthermore, the result of the transplanted tumor model indicated that TMG substantially enhanced the activities of Bax and caspase-3, reduced the activity of Bcl-2, and suppressed the expression of Raf/MEK/ERK protein levels. Fecal analysis revealed that TMG reconstructed the gut microbiota in colorectal cancer-affected mice by augmenting the abundance of the advantageous bacterium *Lactobacillus* and decreasing the abundance of the harmful bacterium *Proteus*.

**Conclusion:**

TMG can exhibit potent anti-colorectal cancer effects through diverse apoptotic mechanisms, with their mode of action closely related to the regulation of gut microbiota.

## 1 Introduction

Globally, cancer is one of the most life-threatening diseases, and its incidence continues to increase (Gan et al., 2020). The induction of apoptosis in cancer cells is an important anti-tumor mechanism and one of the primary approaches to cancer treatment (Gan et al., 2020). Apoptosis is mainly triggered in different ways by corresponding signaling after the activation of receptors (Li-Weber, 2013). However, because cysteine proteases are responsible for cleaving many important cellular substrates, most apoptotic pathways ultimately affect them, and these cysteoaspartic enzymes have important roles in the apoptotic process (Qiao and Wong, 2009). Apoptosis is influenced by two distinct pathways: the mitochondrial pathway and the extrinsic death receptor pathway (Carneiro and El-Deiry, 2020). They can activate cysteoaspartase directly, leading to pro-apoptotic effects, through signaling mechanisms involving Bcl-2 family proteins (such as Bcl-2 and Bax) and the tumor necrosis factor (TNF) family (Jan and Chaudhry, 2019). It is also known that other processes, particularly the Raf/MEK/ERK signaling pathway, indirectly activate caspases by regulating apoptosis-related genes, which, in turn, stimulate mitochondria and death receptors to initiate the caspase-related apoptotic process (Zuo et al., 2015).

For thousands of years, various Asian countries have employed ginseng (*Panax ginseng C.A.Meyer*) as a traditional herbal remedy (Ratan et al., 2021). An essential component of ginseng, ginsenoside can exhibit a wide variety of pharmacological actions that include enhancing immunological function and acting as a potent antioxidant, anti-aggregation, anti-inflammatory, anti-stress, anti-fatigue, and anti-tumor agent (Song et al., 2022). Many studies have previously demonstrated that ginsenosides can exhibit significant anti-tumor effects in lung, colon, and liver cancer models (Hu et al., 2023). Ginsenosides have been shown to exhibit anti-tumor effects in both *in vitro* and *in vivo* cancer models. These effects include inducing apoptosis, inhibiting cancer cell proliferation and metastasis, promoting immunity, and hindering angiogenesis (Kim et al., 2021). For instance, ginsenoside Rb1 can promote apoptosis while reducing the development of uterine fibroid cells (Zhang et al., 2019). Ginsenoside Rg1 can prevent mitotic activities such as chromosomal alignment and spindle dynamics, which are necessary for stimulating the proliferation of cancer cells (Hong et al., 2022). Interestingly, it has been found that red ginseng contains increased levels of saponin Rg3 in comparison to white ginseng, thus further confirming its positive anti-proliferative effect on colorectal cancer cells, which, in turn, can exert anticancer effects (Fishbein et al., 2009). In addition, Wan et al. (2022) have also reported that heat-treated American ginseng berries exhibited a substantial anti-proliferative effect on gastric cancer cells, and the number of apoptotic cells was also significantly increased (Wan et al., 2022). This was mainly attributed to the decrease in the content of major ginsenosides such as Rg1, Re, and Rb1 and the increase in the content of minor ginsenosides such as Rg3 and Rg2 after the heat treatment. Moreover, some minor ginsenosides have been found to display significant anticancer activity. For example, ginsenoside CK can effectively inhibit the proliferation of gastric carcinoma HGC-27 cells and induce apoptosis by modulating the PI3K/AKT/NF-κB pathway (Wan et al., 2022). During the G0/G1 phase, ginsenoside Rg3 showed the ability to interrupt the EGFR/Ras/Raf/MEK/ERK signaling pathway, thereby resulting in decreased proliferation and induction of apoptosis in A549 lung cancer cells (Liang et al., 2021).

In this study, we obtained total minor ginsenosides (TMG) through the fermentation of total ginsenosides extracted by *Lactobacillus plantarum* MB11 and then purified by chromatography on a macroporous resin column (Shen et al., 2023). TMG is a mixture of eight minor ginsenosides such as Rg6, F4, Rk3, Rh4, 20(R)-Rg3, 20(S)-Rg3, CK, and Rh2. At present, there are many studies on the anti-cancer activity of minor ginsenoside monomers such as Rg3, CK, and Rh4 (Liu J. et al., 2022; Wu L. et al., 2024; Ying et al., 2023). Based on the chemical composition of minor ginsenosides contained in TMG, we believe that TMG may be used for the treatment of cancer. This assumption, together with the need for novel therapeutic strategies for colon cancer, leads us to investigate the anti-carcinogenic effects of TMG on colon cancer cells *in vitro* and *in vivo* and elucidate the mechanism of its action. As a result, in the current study, we further investigated the function and mechanism of TMG against colorectal cancer by investigating their impact on the survival and apoptotic rate of CT26.WT cells *in vitro* and protein levels of the Raf/MEK/ERK pathway and apoptotic factor Bax/Bcl-2/caspase-3 in a colorectal cancer mouse model. Furthermore, 16S ribosomal RNA (rRNA) gene sequencing targeting the V3–V4 region was used to profile the gut microbiota.

## 2 Materials and methods

### 2.1 Preparation of total minor ginsenosides

TMG were obtained through *L. plantarum* MB11 fermentation of the major ginsenosides in De Man, Rogosa, and Sharpe (MRS) broth at 37°C for 21 days. Based on previous research, the fermentation broth was freeze-dried, subjected to D101 macroporous resin column chromatography, and eluted with water and 30%, 50%, and 70% (v/v) ethanol (Shen et al., 2023). The eluted fraction with 70% ethanol contained the desired ginsenosides. This fraction was then vacuum-concentrated and lyophilized to obtain a powdered product, referred to as TMG, which served as the test sample. TMG were examined using high-performance liquid chromatography (HPLC) and liquid chromatography mass spectrometry (LC-MS) techniques to determine its composition, which is principally composed of the following ginsenosides: Rg6 (3.2 ± 0.4 mg/g), F4 (4.4 ± 0.3 mg/g), Rh4 (43.3 ± 0.8 mg/g), Rk3 (80.2 ± 1.2 mg/g), 20(R)-Rg3 (185.5 ± 3.5 mg/g), 20(S)-Rg3 (55.1 ± 0.6 mg/g), CK (37.6 ± 0.7 mg/g), and Rh2 (165.2 ± 4.5 mg/g).

### 2.2 Cell lines and cell culture

The CT26.WT cells were procured from Wuhan Servicebio Technology Co., Ltd., Wuhan, China. The cells were maintained in the RPMI-1640 medium supplemented with 10% fetal bovine serum, 100 IU/mL penicillin, and 100 μg/mL streptomycin. They were cultured in a humidified environment at 37°C with 5% CO_2_ for 24 h, with passaging performed every 2–3 days.

### 2.3 Cell viability assay

Cell viability analyses were performed using the CCK-8 assay. (US Everbright Inc.). CT26.WT cells were seeded in 6-well plates at a density of 1.0 × 10^4^ cells/well and allowed to incubate for 24 h before the treatment. Subsequently, the cells were exposed to TMGs at concentrations of 0, 25, 50, 100, and 200 μg/mL, all dissolved in PBS. The cell culture was then incubated in a CO_2_ incubator for 48 h at 37°C and 5% CO_2_. Subsequently, the cells were incubated in a 10% (v/v) CCK-8 solution for 3 h. The absorbance was measured at 450 nm using a SpectraMax ABS Plus Microplate Reader from Molecular Devices, Shanghai, China (Kong et al., 2021).

The cell viability was calculated using the following formula:

$$\text{Cell viability }\%=\frac{A_{\text{test}}}{A_{\text{control}}}\times 100\%.$$

### 2.4 TUNEL assay

The apoptosis state of CT26.WT cells was evaluated by performing the TUNEL assay. In a 6-well plate, 2 × 10^5^ CT26.WT cells were cultured with sterile coverslips and exposed to control (0 μg/mL), cyclophosphamide (CTX) (100 μg/mL), and TMG (200 μg/mL) for 24 h; in this experiment, CTX served as the positive control. After reaching the desired size, cells were washed thrice with PBS and fixed with 4% paraformaldehyde for 30 min. Following fixation and another round of washing with PBS, the cells were treated with 0.3% Triton X-100 for 20 min at room temperature, followed by another three PBS washes. Next, the cells were covered with TUNEL kit buffer (provided by Wuhan Service Biotechnology Co., Ltd.) and incubated at room temperature for 10 min. A mixture of TDT enzyme: dUTP: buffer (1:5:50) was applied to the cells and incubated at 37°C for 2 h. After three 5-min PBS washes, the PBS was removed, and the cells were stained with the DAPI dye. The samples were incubated in the dark at room temperature for 10 min, and the images were captured under a fluorescence microscope for observation. The number of TUNEL-positive cells was determined using ImageJ software (Wang et al., 2022).

### 2.5 Animal and experimental protocol

A total of 30 BALB/c mice (female, weighing 20–22 g) were obtained from Liaoning Changsheng Biotechnology Co., Ltd. (Liaoning, China) and acclimated for 1 week prior to the study’s start. All animal procedures followed the criteria established by the Jilin Academy of Agricultural Sciences’ Animal Care and Ethics Committee (Ethical reference number: 57/2021). The mice’s right flanks were subcutaneously implanted with a suspension of 1 × 10^7^ live CT26.WT cells in 0.2 mL PBS for inducing tumors. Once the tumors reached a size of approximately 0.5 cm (Liu et al., 2005), the mice were randomly assigned to three groups: the model group, the CTX group, and the TMG group. In the TMG group, mice received a dose of 100 mg/kg/day of TMG administered via gavage. The CTX group received intraperitoneal injections of CTX at a dose of 25 mg/kg/day via gavage. The model group was administered 0.9% NaCl via gavage. All mice received 21 days of drug administration before being euthanized to collect feces and tumor tissues, which were immediately frozen in liquid nitrogen and stored at −80°C for further analysis.

### 2.6 Western blotting

The transfected cells were collected from each group after 48 h of cultivation. The CT26.WT cells and tumor tissues were lysed with the RIPA lysis buffer, followed by centrifugation to collect the supernatant. The protein concentration was determined using a BCA Kit. Protein samples were resolved on 8%, 10.0%, or 12% SDS-PAGE by boiling with denaturation buffer [1.5 mol Tris-HCl, 10% sodium dodecyl sulfate (SDS), TEMED, and 10% ammonium persulfate]. After that, the samples were incubated for 40 min on a polyvinylidene fluoride (PVDF) membrane obtained from Millipore (Massachusetts, United States). A measure of 3% BSA in Tris-buffered saline with 0.05% Tween-20 (TBST) was used for the membrane blocking. Next, the membrane was treated with the various primary antibodies at a temperature of 4°C for 12 h. The primary antibodies used were anti-β-actin (GeneTex, GTX629630), anti-Raf (CST, #9422), anti-phosphorylated (p)-Raf (CST, #9421), anti-MEK (Bioss, bs-1433R), anti-p-MEK (Bioss, bs-3270R), anti-ERK1/2 (GeneTex, GTX134462), anti-p-ERK1/2 (GeneTex, GTX24819), anti-Bcl-2 (Bioss, bs-4563R), anti-caspase-3 (CST, #9661), anti-cleaved-caspase-3 (CST, #9661), anti-Bax (Proteintech, 50599-2-Ig), and anti-GAPDH (Proteintech, 60004-1-Ig). After this, the membrane was exposed to horseradish peroxidase (HRP)-conjugated secondary antibody at 37°C for 1 h. The quantification of the immunoreactive bands was carried out by utilizing Image Quant LAS 4000 (Fuji Film, Tokyo, Japan) software by using β-actin and GAPDH as the loading controls.

### 2.7 Immunohistochemical staining

The tumors were fixed in 4% paraformaldehyde before being implanted in the paraffin block. Thereafter, the slides with 5-μm-thick sections were made. These sections were dewaxed with xylene and rehydrated progressively with graded alcohol solutions. They were then incubated with 3% hydrogen peroxide for 25 min at room temperature away from light. Following that, antigen retrieval was carried out in PBS (pH = 7.4). The sections were then exposed to different primary antibodies, including anti-cleaved-caspase-3 (1:200, GB11532, Servicebio), anti-Bcl-2 (1:500, GB113375, Servicebio), and anti-Bax (1:400, GB114122, Servicebio). After blocking with 10% goat serum for an hour, the sections were incubated with the primary antibody at 4°C overnight. The slices were then exposed to an HRP-labeled secondary antibody. DAB staining was employed, followed by counterstaining with hematoxylin. The slides were visualized using a bright-field microscope (Nikon ECLIPSE E100, Nikon, Tokyo, Japan).

### 2.8 Gut microbial analysis

The mouse feces were collected aseptically, and the samples were sent to Sangon Biotech Co., Ltd. (Shanghai, China) for paired-end sequencing on the Illumina MiSeq platform, PCR amplification of the V3–V4 hypervariable region, and the total DNA extraction of the fecal microbiota. The sequencing information has been uploaded to the National Center for Biotechnology Information database, and the accession number is Project No. PRJNA1003433. In addition, for *alpha* (*α*)-diversity analysis, Chao1, Shannon, Simpson, and Goods indexes were used to evaluate the microbial diversity in each group. Beta (*β*)-diversity was measured using the principal coordinates analysis. The unweighted UniFrac distance was employed to determine the between-group differences. Relative abundances at the phylum and genus levels were analyzed to understand the microbial community structure. The potential association between the gut microbiota and apoptosis was evaluated by microorganisms, and the correlation between apoptotic variables and microorganisms was examined by Spearman’s analysis.

### 2.9 Statistical analysis

Each data displays the mean value and standard deviation (SD). We used one-way ANOVA and Tukey’s multiple comparison test to examine whether there were any significant differences. Additionally, the data were collected and made visually appealing by creating bar and line charts using the Origin 8.0 program. The threshold for statistical significance was *p* < 0.05.

## 3 Result

### 3.1 TMG reduced the viability of CT26.WT cells

**FIGURE 1:**
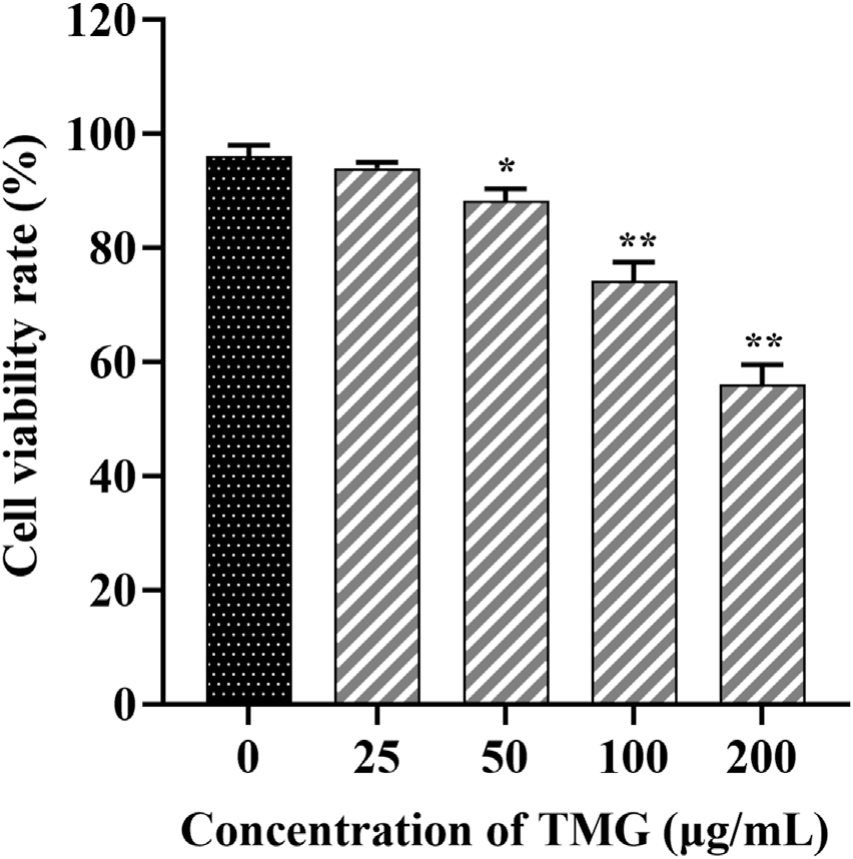
The effects of TMG on the viability of CT26.WT cells were investigated. After treating with different doses of the TMG (0, 25, 50, 100, and 200 μg/mL) for 24 h as indicated, the viability was assessed using the CCK-8 assay. The data are presented as the mean ± SD from experiments conducted in triplicate. The statistical analysis used one-way ANOVA, and pairwise comparisons were carried out using Tukey’s *post hoc* test, implemented through the SPSS Statistics version 20. The statistical significant level indications were *p < 0.05 and **p < 0.01 compared to the control group.

Figure 1 illustrates that TMG inhibited the proliferation of CT26.WT cells in a dose-dependent manner (at concentrations of 0, 25, 50, 100, and 200 μg/mL). Significant inhibition was observed at a concentration of 200 μg/mL after 24 h of TMG treatment, with the inhibition rate reaching 43.97%. A concentration of 25 μg/mL showed no significant inhibitory effect on CT26.WT cells, with a cell viability rate of 94.36% ± 1.78%. In contrast, 50 μg/mL concentration exhibited a modest inhibitory effect, resulting in a slightly reduced cell viability rate of 88.31% ± 1.63% (*p* < 0.05).

### 3.2 TMG induces CT26.WT cell apoptosis

**FIGURE 2:**
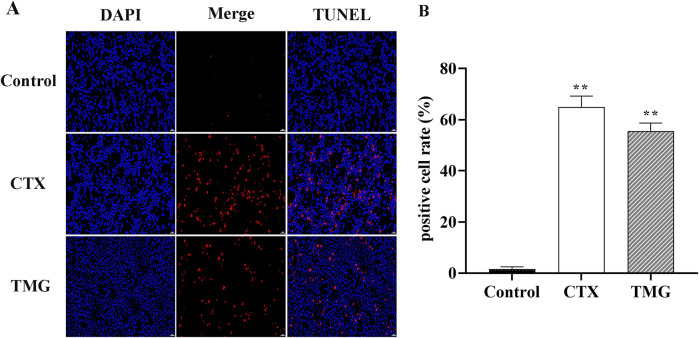
Effect of TMG on the apoptosis rate of CT26.WT cells. **(A)** TUNEL assay to determine cell apoptosis in CT26 cells in each group under 20 × visual field. **(B)** Quantitative analysis of TUNEL staining in each group. The data were analyzed using one-way ANOVA, indicating statistical significance at *p < 0.05 and **p < 0.01 compared to the control group. All values are reported as the mean ± SD.

To further characterize the apoptotic cell death by TMG, we performed the TUNEL staining assay (Figure 2A). TMG-treated cells were stained with TUNEL, and fluorescence intensity was found to be increased. The apoptotic cell number of CT26.WT colon cancer cells reached 54.01% ± 5.14% (*p* < 0.01) after treatment with TMG for 24 h (Figure 2B). Therefore, TMG could exhibit substantial anti-tumor effects on CT26.WT cell by inducing apoptosis.

### 3.3 Effects of TMG on CT26.WT cell cleaved-caspase-3, caspase-3, Bcl-2, and Bax expression

**FIGURE 3:**
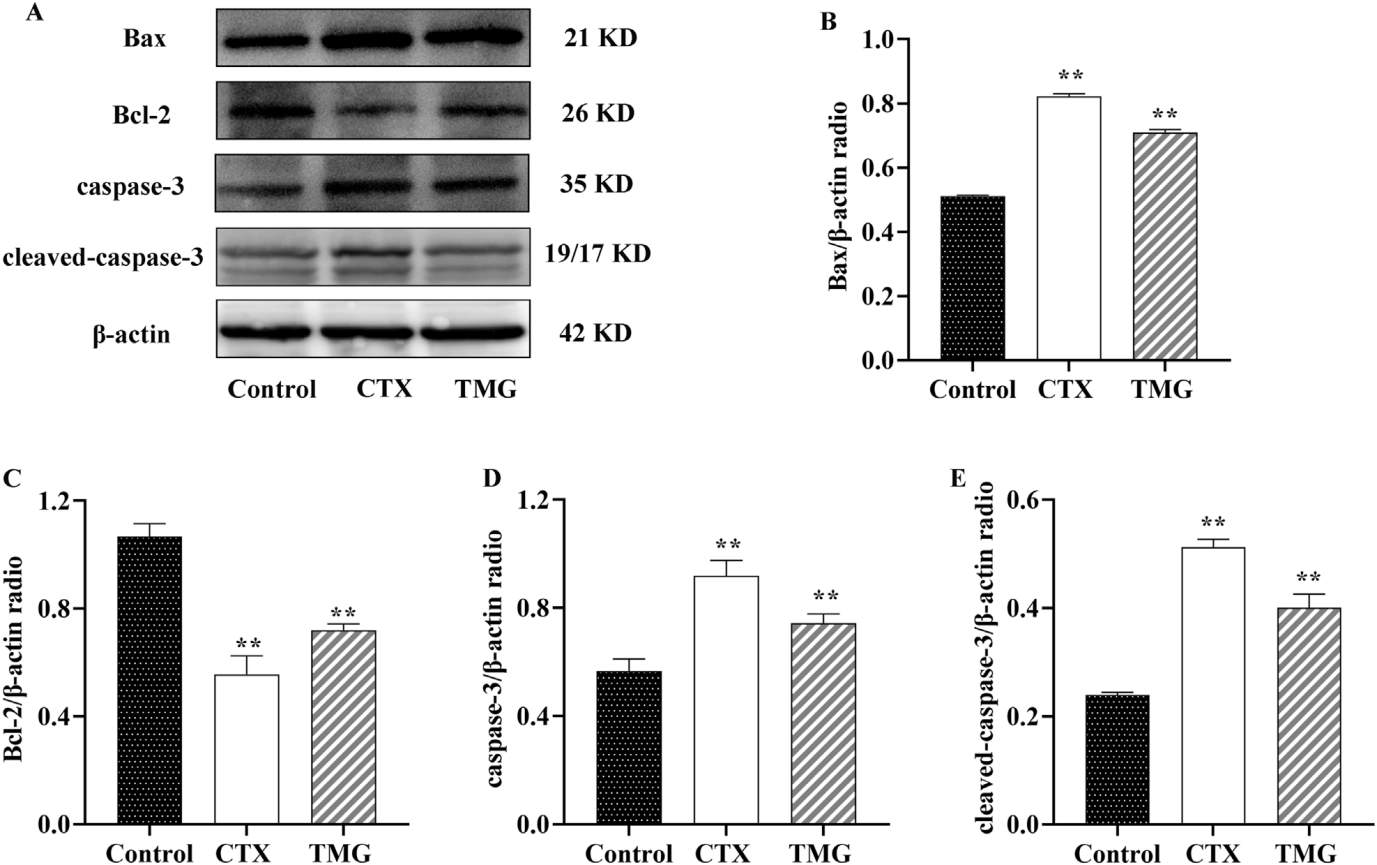
Expressions of Bax, Bcl-2, caspase-3, and cleaved-caspase-3 in CT26.WT cells. **(A)** Representative Western blot images. **(B–E)** Quantitative analysis of Western blot results, showing the ratio of proteins. All data were analyzed by one-way ANOVA: *p < 0.05 and **p < 0.01 vs. control group; n = 3. The data are presented as the mean ± SD. β-actin was used as the standard control.

The protein expression of apoptosis-related markers, including Bax, Bcl-2, caspase-3, and cleaved-caspase-3, was analyzed by Western blotting on CT26.WT cell lines (Figure 3A). When compared to the control group, CT26.WT cells treated with TMG showed a significant reduction in Bcl-2 protein expression by 32.45% (Figure 3B, *p* < 0.05). In contrast, untreated control cells exhibited lower levels of Bax, caspase-3, and cleaved-caspase-3 proteins. Specifically, the protein levels of Bax, caspase-3, and cleaved-caspase-3 increased by 38.46%, 27.88%, and 67.41%, respectively, in the treated cells, respectively (Figures 3C–E, *p* < 0.05).

### 3.4 Impact of TMG therapy on caspase-3, Bcl-2, and Bax expression in the CT26-bearing mice

**FIGURE 4:**
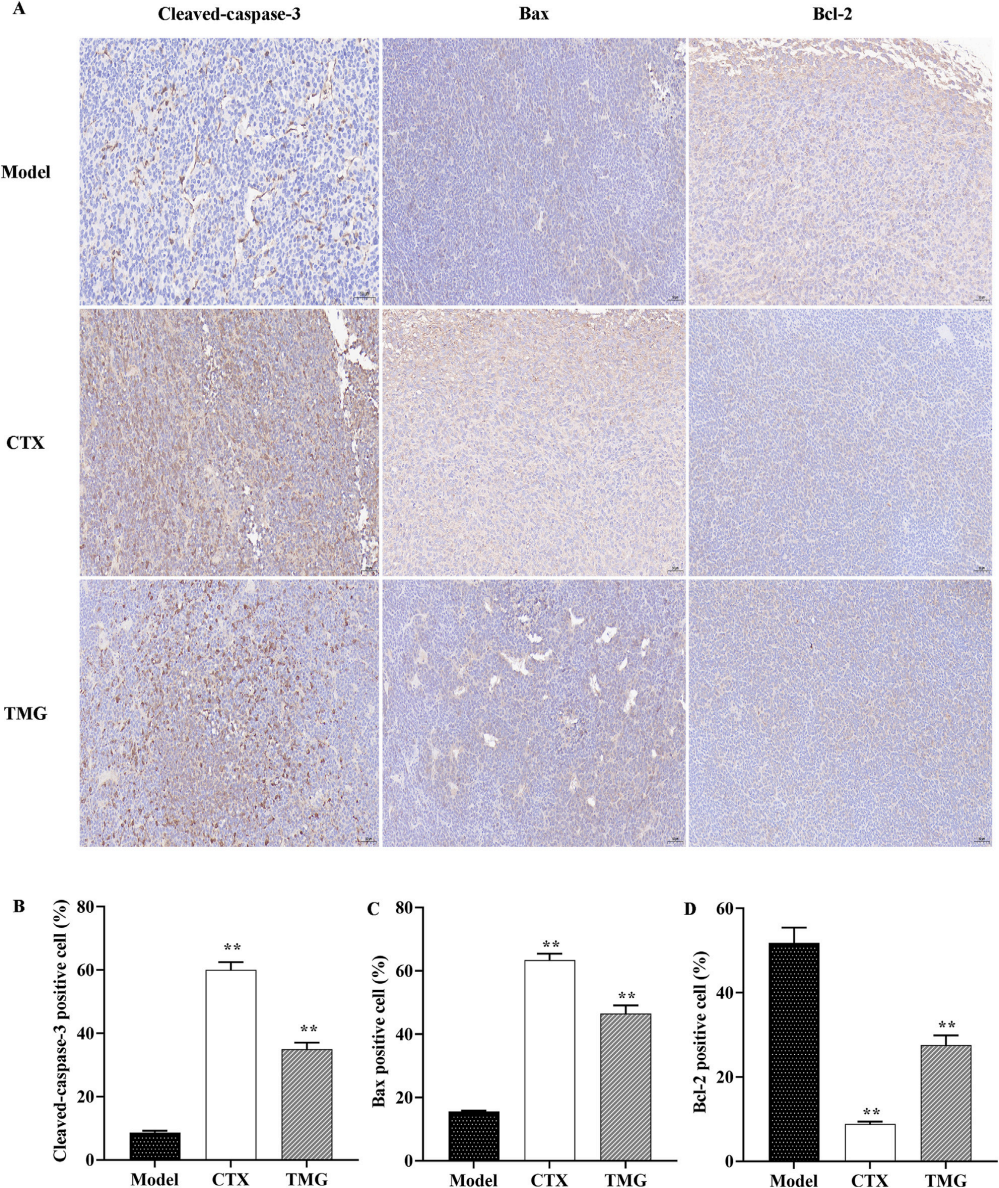
TMG inhibit colon carcinoma xenograft tumor growth. IHC was used to identify Bax, Bcl-2, and cleaved-caspase-3 expression in tumor tissue (microscope: 200x). **(A)** Quantitative analysis of IHC for the cleaved-caspase-3, Bax, and Bcl-2 protein expression. **(B-D)** Data are presented as the means with standard deviations (error bars); n = 3 for each group. The comparison between the TMG and model groups shows a calculated significance of **p < 0.01.

The results indicated that TMG at a dose of 25 mg/kg significantly increased (*p* < 0.05) the expression of cleaved-caspase-3 (Figure 4B) and Bax (Figure 4C) compared to the model. Moreover, TMG had a significant inhibitory effect (*p* < 0.05) on the expression of Bcl-2 in the transplanted mouse CT26 colorectal cancer tissues, as shown in Figure 4D. Upon treatment with TMG, the percentage of cells positive for cleaved-caspase-3 increased significantly to 35.02%. Similarly, the percentage of cells positive for Bax also increased significantly, reaching 46.49%. On the contrary, the percentage of positive cells for Bcl-2 decreased to 27.53% following treatment with TMG.

### 3.5 TMG exerts anticancer activity by inhibiting the Raf/MEK/ERK signaling pathway in the CT26-bearing mice

**FIGURE 5:**
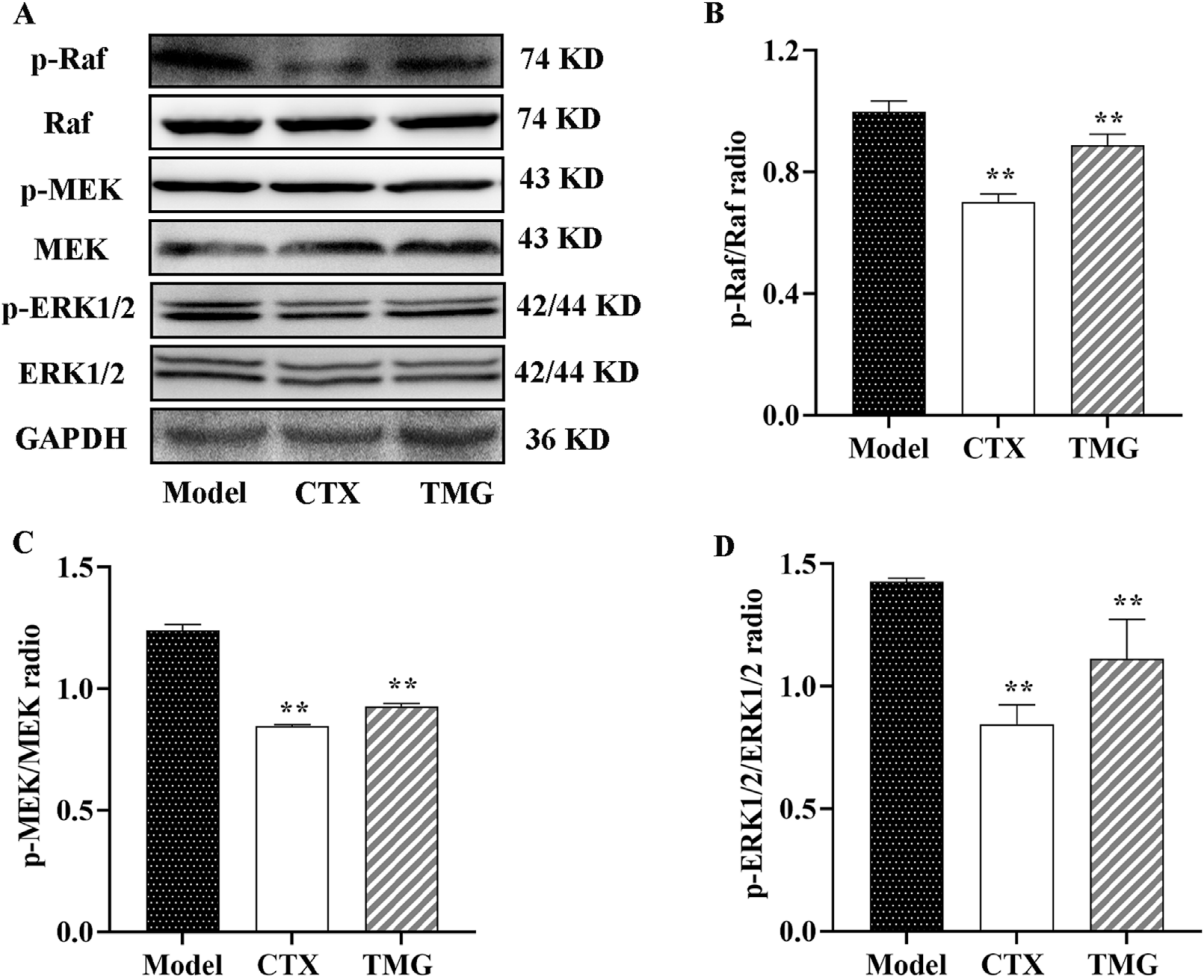
Expressions of phosphorylated and unphosphorylated Raf, MEK, and ERK1/2 in the CT26-bearing mice. **(A)** Representative Western blot images. **(B–D)** Quantitative analysis of Western blot result, showing the ratio of phosphorylated/unphosphorylated proteins. All data were analyzed by one-way ANOVA: *p < 0.05 and **p < 0.01 vs. model group; n = 3. The data were presented as the mean ± SD. GAPDH was used as the standard control.

To further characterize the mechanism underlying the inhibition of cell proliferation by TMG, we examined whether it affected Raf, MEK, and ERK expressions in the tumor tissues using the Western blotting analysis (Figure 5A). The study found that compared with the treatment group, the model group had higher levels of p-Raf, p-MEK, and p-ERK1/2, but the expression levels of these proteins were significantly inhibited by TMG by 15.37%, 27.41%, and 33.1%, respectively (*p* < 0.01, Figures 5B–D).

### 3.6 Effects of TMG on the composition of the gut microbiota in the CT26-bearing mice

**FIGURE 6:**
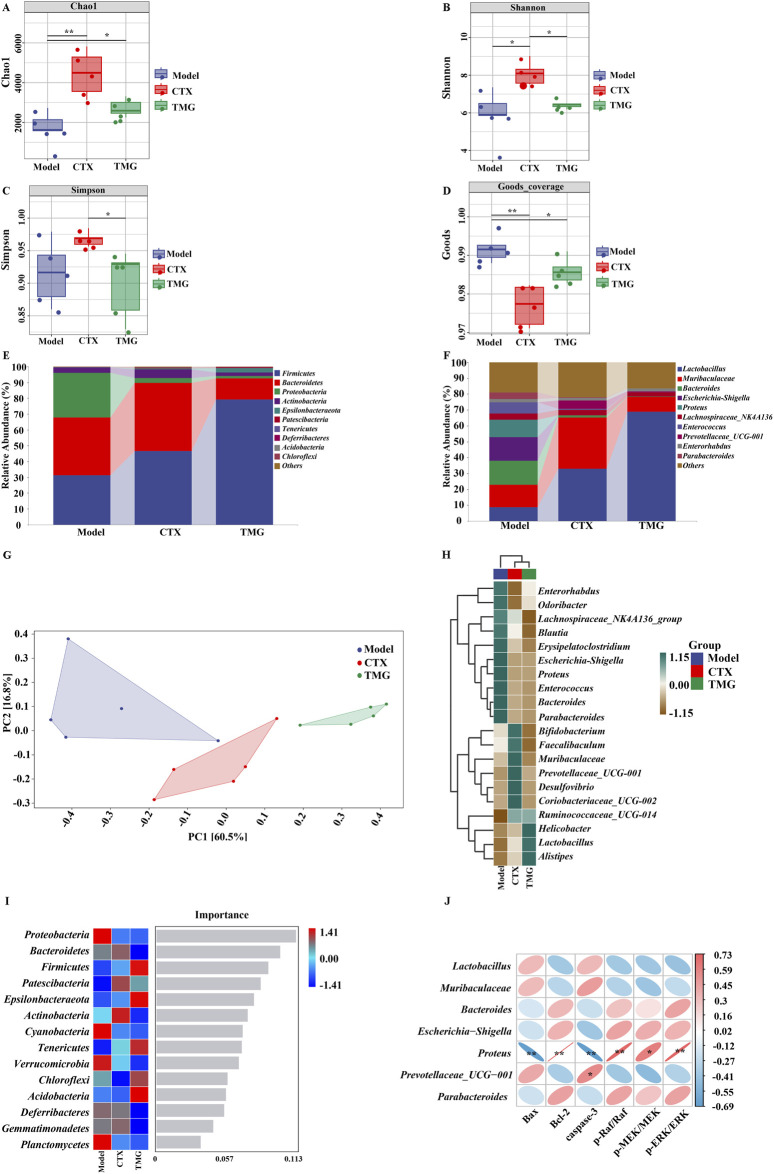
TMG effects on the diversity and composition of the gut microbiota. Chao1 **(A)**, Shannon **(B)**, Simpson **(C)** and Goods **(D)** indices. Species composition at the phylum level **(E)**. Species composition at the genus level **(F)**. PCoA analysis based on unweighted UniFrac phylogeny distances **(G)**. Heatmaps of each group of samples at the phylum level **(H)**. Number of important species in an area **(I)**. Correlation between the gut microbiota and apoptosis factors, where red indicates a positive correlation and blue indicates a negative correlation **(J)**. *p < 0.05 and **p < 0.01 vs. model group (n = 5).

To further illustrate the holistic mechanisms underlying the effects of TMG on CT26-bearing mice, we explore the influence of TMG on the gut microbiota by high-throughput DNA sequencing analysis of 16S rRNA in fecal bacteria across all groups of mice. First, we performed α-diversity analysis, in which the Chao1 index reflects the community richness, the Shannon and Simpson indexes visualize the species diversity, and the Goods index represents the coverage of the sequencing libraries for each individual sample. Compared with the model group, the treatments in both the CTX and TMG groups effectively improved the Chao1 index by 161.17% and 59.74%. (Figure 6A, *p* < 0.05), respectively. For the Shannon index, a significant difference was observed between the CTX and model groups (Figure 6B, *p* < 0.05), but there was no significant difference between the TMG and model groups. Additionally, in the context of the Simpson index, compared with the model group, both the CTX and TMG groups displayed insignificant differences (Figure 6C). Furthermore, compared to the model group, both the CTX and TMG groups showed significant differences in the Goods index (*p* < 0.05). Notably, the Goods index for each group was greater than 97%, indicating that a substantial majority of sequences within each group were accurately identified. This high percentage emphasizes that the sequencing results can reflect the composition and diversity of the microorganisms in the samples (Figure 6D). To clarify which bacteria were altered by TMG, the top 10 species of the highest abundance at various taxonomic levels were analyzed (Figures 6E,F). At the phylum level, the probiotic phylum *Firmicutes* composed the majority in all samples, and it was downregulated in CT26-bearing mice, whereas TMG evidently reversed the decline in *Firmicutes* abundance (Figure 6E). At the genus level, the probiotic phylum *Lactobacillus* constituted the majority in all samples. In CT26-bearing mice, it was observed that *Lactobacillus* abundance was diminished; however, TMG were found to effectively reverse this decline (Figure 6F). Meanwhile, TMG decreased the fecal abundances of *Proteobacteria* in CT26-bearing mice. *β*-diversity at the operational taxonomic unit (OTU) level was analyzed by principal component analysis (PCA), whose scatter plot showed that the model group mice were far different from those in the other two groups, which were clustered, indicating that the principal component of fecal bacteria in CT26-bearing mice was altered, and TMG decreased the *β*-diversity of the gut microbiota in CT26-bearing mice (Figure 6G). We compared the groups by plotting a heatmap of species abundance at the genus level (Figure 6H). In the model group, *Helicobacter*, *Lactobacillus*, and *Alistipes*, which are beneficial strains, were less abundant, while *Enterococcus*, *Proteus*, and *Blautia*, which are harmful strains, were more abundant. However, TMG treatment significantly improved the abundance of these floras.

Notably, *Proteobacteria* emerged as a marker species for discernible distinctions between groups at the phylum level, as revealed by the forest randomization analysis (Figure 6I). Furthermore, the possible connection between gut microbiota and apoptosis factors was further verified through Spearman analysis (Figure 6J). This analysis indicated a potential positive correlation between *Proteus* and the levels of Bcl-2 (*p* = 0.002 and R = 0.73), p-Raf/Raf (*p* = 0.003 and R = 0.72), p-MEK/MEK (*p* = 0.017 and R = 0.61), and p-ERK/ERK (*p* = 0.002 and R = 0.72), but it exhibited a negative correlation with the levels of Bax (*p* = 0.005 and R = −0.69) and caspase-3 (*p* = 0.004 and R = −0.33) apoptosis factors. These findings collectively suggested that TMG may potentially affect cancer-associated apoptosis by modulating factors such as *Lactobacillus* and *Proteus* (*p* < 0.01).

## 4 Discussion

Ginsenosides are the primary active constituents of ginseng, and minor ginsenosides have been previously reported to exhibit superior anti-colon cancer activity (Tang et al., 2018). For instance, prior studies have shown that ginsenosides can inhibit the growth of colorectal cancer under both *in vitro* and *in vivo* settings by exhibiting anti-inflammatory, autophagy-inducing, and anti-angiogenesis effects (Shao et al., 2022; Tang et al., 2018; Wu et al., 2022). Thus, we prepared a total minor ginsenoside through fermentation and transformation by *Lactobacillus plantarum* and subsequently evaluated its potential effect on colorectal cancer both in controlled laboratory settings and in living organisms. Experiments using CCK-8 and TUNEL assays revealed that treatment with the TMG significantly inhibited cancer cell proliferation. The inhibitory mechanism of these minor ginsenosides on tumorigenesis and development could be related to their impact on Bax, Bcl-2, caspase-3, and Raf/MEK/ERK signaling pathways. In addition, TMGs were found to positively affect the structure of the gut microbiota and the microbial community. These results indicated that the minor ginsenoside complex could induce apoptosis, thereby effectively suppressing colorectal cancer growth.

Apoptosis is an important mechanism responsible for tumor cytotoxicity (Pileczki et al., 2016). In this study, the results of the CCK-8 proliferation assay and TUNEL fluorescence staining analysis indicated that TMG exhibited a significant inhibitory effect on the proliferation of CT26.WT colon cancer cells and induced their apoptosis. Interestingly, evidence from prior studies clearly suggests that ginsenosides, such as Rg3, CK, and Rh2, and other substances can suppress the growth of tumor cells and trigger apoptosis of CRC cells by increasing caspase-3 expression (Han et al., 2016; Yang et al., 2017; Zhang et al., 2013). Ginsenoside Rg3 combined with 5-fluorouracil (5-FU) was able to significantly reduce the viability of CT26.WT colon cancer (Liu Z. et al., 2022). In addition, it has been reported in the literature that minor ginsenoside Rg3 inhibited proliferation and induced apoptosis in HCT116, HT29, and SW480 colorectal cells. Moreover, the results of our Western blot analysis revealed that TMG significantly increased the expression levels of Bax and caspase-3 proteins both *in vivo* and in CT26.WT cells but significantly attenuated the expression of the anti-apoptotic protein Bcl-2. It has been established that the Bcl-2 protein family, consisting of pro- and anti-apoptotic members including Bax and Bad, plays an important role in the regulation of apoptosis. When the Bax/Bcl-2 ratio increases, cytochrome c is released from the mitochondria into the cytoplasm, which can then trigger the activation of caspase-3 (Yamada et al., 2014). In addition, the results of Jiang et al. (2011) also confirmed that ginsenoside Rg3 alone or in combination with CTX induced apoptosis in HCC cells by activating an intrinsic pathway and altering the expression of the Bcl-2 family of proteins, which inhibited tumor growth *in vivo* and prolonged the survival in mice (Jiang et al., 2011).

Increasing evidence suggests that the abnormal activation of the Raf/MEK/ERK pathway plays a key role in regulating the apoptosis of colorectal cancer cells (Li et al., 2014). It was observed that the treatment with TMG activated the Raf/MEK/ERK pathway, leading to anti-tumor effects and apoptosis. Moreover, the administration of TMG activated the Raf/MEK/ERK signaling pathway, resulting in anti-tumor effects and apoptosis. This study also determined the *in vivo* expression of Raf, MEK, ERK, p-Raf, p-MEK, and p-ERK proteins. The results demonstrated a significant reduction in the expression levels of p-Raf, p-MEK, and p-ERK proteins. In agreement with our findings, Huang et al. have previously reported that ginsenoside C3C12PPD inhibited tumor proliferation via the suppression of the Raf/MEK/ERK and AKT/mTOR protein signaling pathways, in addition to the AKT/GSK-3/-linker protein. This resulted in a significant decrease in the expression levels of p-Raf, p-MEK, and p-ERK proteins (Huang et al., 2019). Moreover, ERK phosphorylation can lead to an elevation in Bcl-2, a reduction in Bax, and eventually cause a downregulation of caspase-3 activity (Xu et al., 2016). Our immunohistochemical results confirmed that TMGs could enhance caspase-3 activity. This observation suggested that apoptosis induced by TMG through the inhibition of the Raf/MEK/ERK pathway under *in vivo* conditions might involve Bcl-2/Bax/caspase-3, which further supported the results of the *in vitro* experiments.

In recent years, experiments have revealed that approximately 20% of human malignancies could be linked to bacteria, and the relationship between cancer and microbes is complex (Martel et al., 2012). In addition, imbalances in the gut microbiota and disturbances in the flora structure, such as a decrease in the populations of beneficial bacteria and the presence of *Enterobacteriaceae* and other pathogenic bacteria, can lead to a micro-ecological imbalance that can further damage the cellular DNA, activate oncogenic signaling pathways, and thereby ultimately affect the development of CRC (Elinav et al., 2013; Sun and Kato, 2016). *Proteus*, a pathogenic Gram-negative bacterium that is commonly found in soil and water supplies, has recently been identified to be strongly associated with colorectal cancer (Mobley, 2019). For example, Mobley et al. found that *Proteus mirabilis* was present in the cells of colorectal adenomas and cancers but not in the normal colorectal mucosa cells (Mobley, 2019). Interestingly, we analyzed the relationship between intestinal flora and apoptotic factors by Spearman’s analysis, and the results indicated that TMG treatment significantly decreased the abundance of *Proteus* flora. Moreover, Liu et al. (2023) also hypothesized that *Proteus* could be closely related to the progression of CRC and that an increase in its abundance might promote the development and progression of CRC, which was exactly in line with our results. It was also found that the abundance of *Bacteroidetes* was reduced in TMG-treated colorectal cancer mice and the abundance of *Akkermansia* was also reduced, but it was also observed that the abundance of *Bacteroidetes* was impaired in colorectal cancer patients, whereas the abundance of *Akkermansia* increased and was negatively correlated with tumor volume (Chen et al., 2020). Moreover, recent studies have also confirmed that the oral administration of *Lactobacillus casei* ATCC 393 can significantly inhibit the growth of colorectal cancer cells by a mechanism that could lead to the upregulation of TNF-related apoptosis-inducing ligand (TRAIL) and the downregulation of survivin, a key effector molecule involved in the process of cellular apoptosis (Ghorbani et al., 2022).

Numerous studies have demonstrated a significant correlation between gut microbial dysbiosis and tumorigenesis. Moreover, it has been well established that the gut microbiota not only influences tumor development but also affects the efficacy of tumor therapies (Zhao et al., 2023). In recent years, the role of the intestinal flora in disease control, particularly through the regulation of apoptosis and proliferation pathways, has garnered increasing attention (Deng et al., 2023). The present study supports the hypothesis that TMG enhance the abundance of intestinal flora and modulate related protein pathways, thereby exerting anti-tumor effects. Espley et al. observed that anthocyanin treatment increased the levels of beneficial gut bacteria in mice and reduced inflammatory marker levels, leading to decreased levels of leukotriene B4, prostaglandin E2 (PGE2), and TNF-α. The inhibition of these inflammatory mediators through active phenolic compounds and beneficial gut microbiota suggests a promising strategy for cancer prevention (Espley et al., 2014). Additionally, ginseng fermentation solution (GFS) has been shown to correct intestinal flora imbalances by modulating the PI3K/Akt pathway, thereby reducing Bax protein expression and increasing Bcl-2 protein expression. Notably, an increased abundance of the *Aspergillus* phylum has demonstrated a protective effect against alcoholic liver disease (Wu Y. et al., 2024). These findings indicate that TMG have the potential to regulate apoptosis-related factors and pathways, as well as maintain a balance between beneficial and harmful gut bacteria, leading to an anti-colorectal cancer effect. This study further highlights that ginsenoside fermentation by *Lactobacillus plantarum*, a probiotic, not only enhances the bioactivity of ginsenosides but also mitigates the impact of harmful elements. This approach offers a novel strategy for future CRC treatment and enhances drug bioavailability.

## 5 Conclusion

In conclusion, our research findings indicate that TMG can inhibit the proliferation of CT.26.WT cells and induce their apoptosis *in vitro*. *In vivo*, TMG-induced tumor growth inhibition was observed in mice with colorectal cancer, mediated by the regulation of the Raf/ MEK/ ERK pathway. Furthermore, both *in vivo* and *in vitro* experiments have demonstrated that TMG reduces the expression of pro-apoptotic factors, such as Bax, Bcl-2, and caspase-3. Consequently, this effect leads to anti-tumor activity. Finally, TMG can also reduce *Proteus* by modulating the intestinal microbiota while increasing the abundance of *Lactobacillus*; *Proteus* has also been shown to be associated with apoptotic factors, such as caspase-3, and regulatory proteins. Considering these findings, TMG may exert an anti-colorectal cancer effect by improving the balance of intestinal microbes and affecting the Raf/MEK/ ERK pathway and apoptosis-related factors.

## Data Availability

The datasets presented in this study can be found in online repositories. The names of the repository/repositories and accession number(s) can be found in the article/supplementary material.
