# Supplementary material for: Anti-colorectal cancer effect of total minor ginsenosides produced by lactobacilli transformation of major ginsenosides by inducing apoptosis and regulating gut microbiota
Source: Front Pharmacol. 2025 Jan 8;15:1496346. doi: 10.3389/fphar.2024.1496346 (PMC11750747; doi:10.3389/fphar.2024.1496346)
Supplement: Supplementary file 1 [file DataSheet1.pdf]

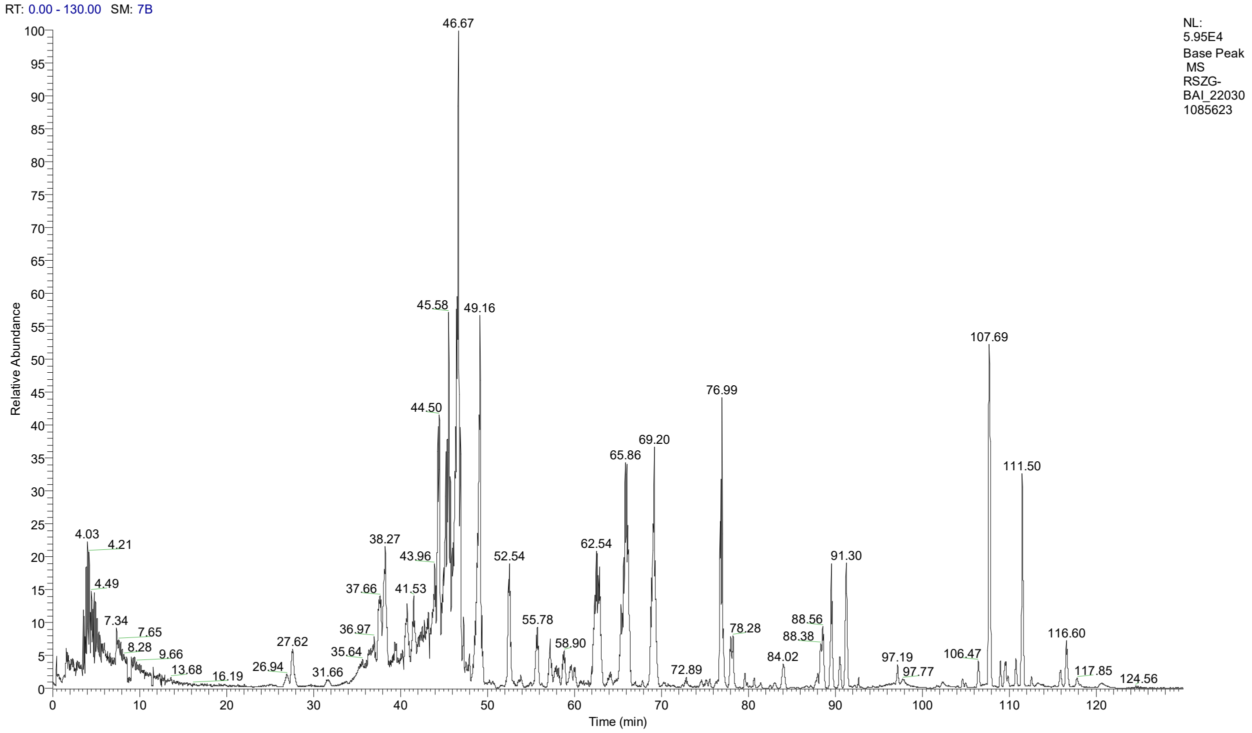
RT: 0.00 - 130.00 SM: 7B
NL: 5.95E4
Base Peak MS
RSZG-BAI_220301085623
Relative Abundance
Time (min)
46.67
45.58
49.16
107.69
76.99
44.50
69.20
65.86
111.50
4.03
4.21
38.27
62.54
91.30
52.54
43.96
4.49
37.66
41.53
88.56
55.78
116.60
7.34
7.65
78.28
88.38
58.90
8.28
9.66
27.62
36.97
35.64
84.02
97.19
106.47
13.68
16.19
26.94
31.66
72.89
97.77
117.85
124.56

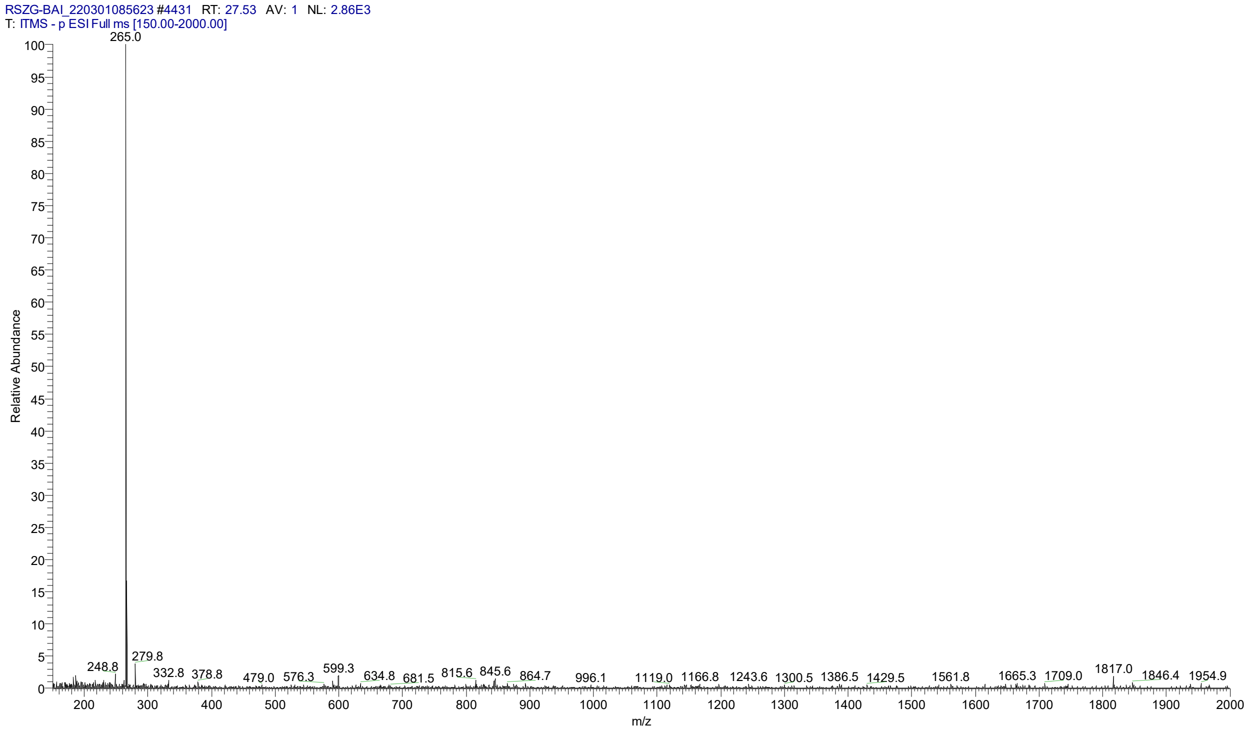
RSZG-BAI_220301085623 #4431 RT: 27.53 AV: 1 NL: 2.86E3
T: ITMS - p ESI Full ms [150.00-2000.00]
Relative Abundance
m/z
265.0
279.8
248.8
332.8
378.8
479.0
576.3
599.3
634.8
681.5
815.6
845.6
864.7
996.1
1119.0
1166.8
1243.6
1300.5
1386.5
1429.5
1561.8
1665.3
1709.0
1817.0
1846.4
1954.9

RSZG-BAI_220301085623 #5092 RT: 31.67 AV: 1 NL: 7.50E2
T: ITMS - p ESI Full ms [150.00-2000.00]

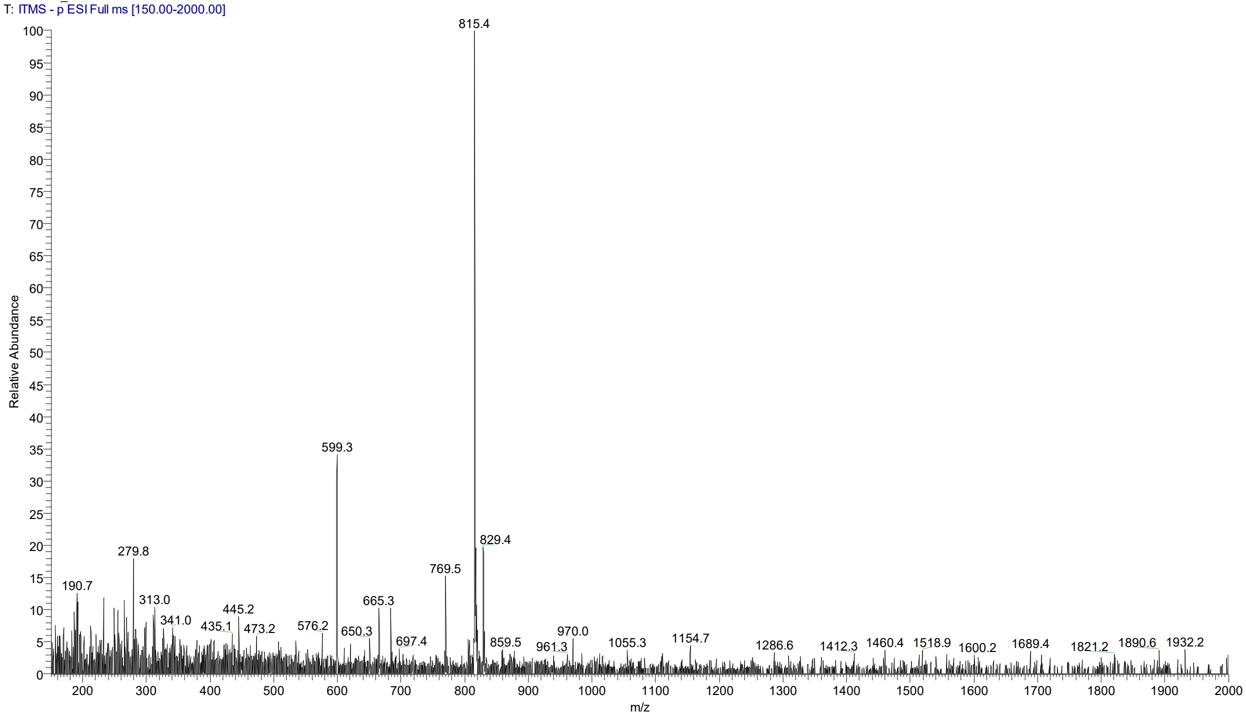

RSZG-BAI_220301085623 #6053 RT: 37.69 AV: 1 NL: 8.36E3
T: ITMS - p ESI Full ms [150.00-2000.00]

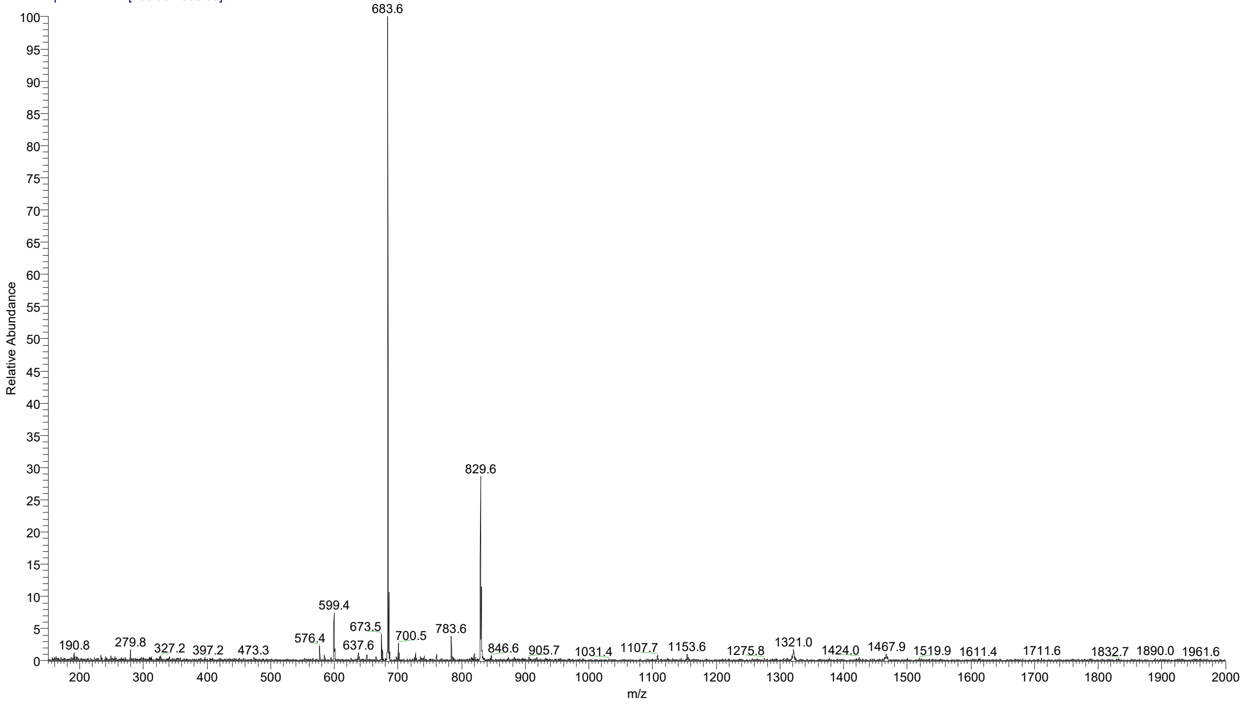

RSZG-BAI_220301085623 #6125 RT: 38.14 AV: 1 NL: 1.09E4
T: ITMS - p ESI Full ms [150.00-2000.00]

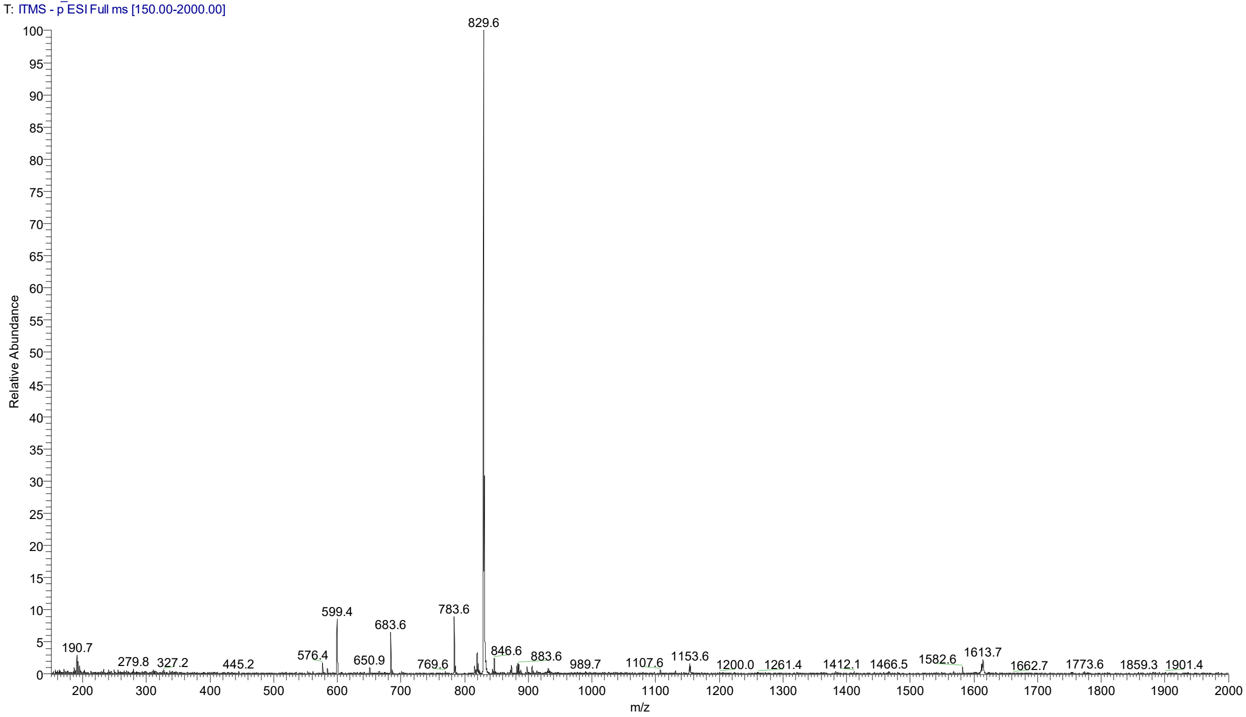

RSZG-BAI_220301085623 #6527 RT: 40.66 AV: 1 NL: 5.26E3
T: ITMS - p ESI Full ms [150.00-2000.00]

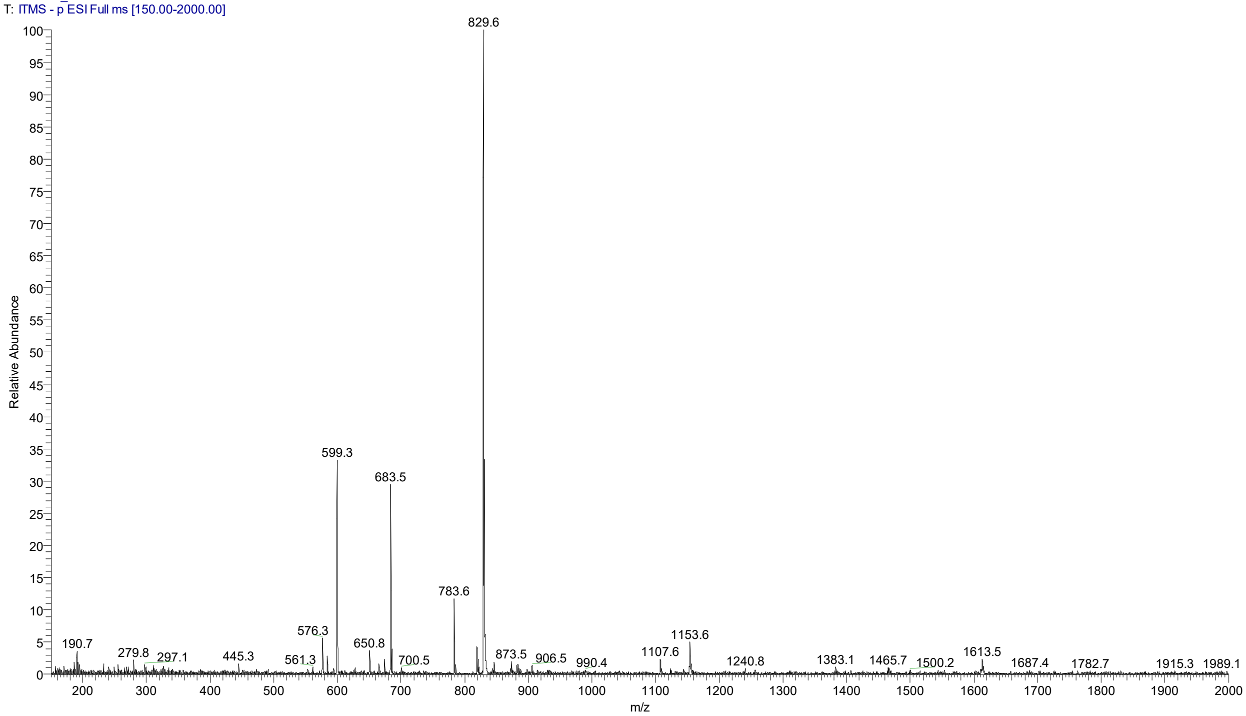

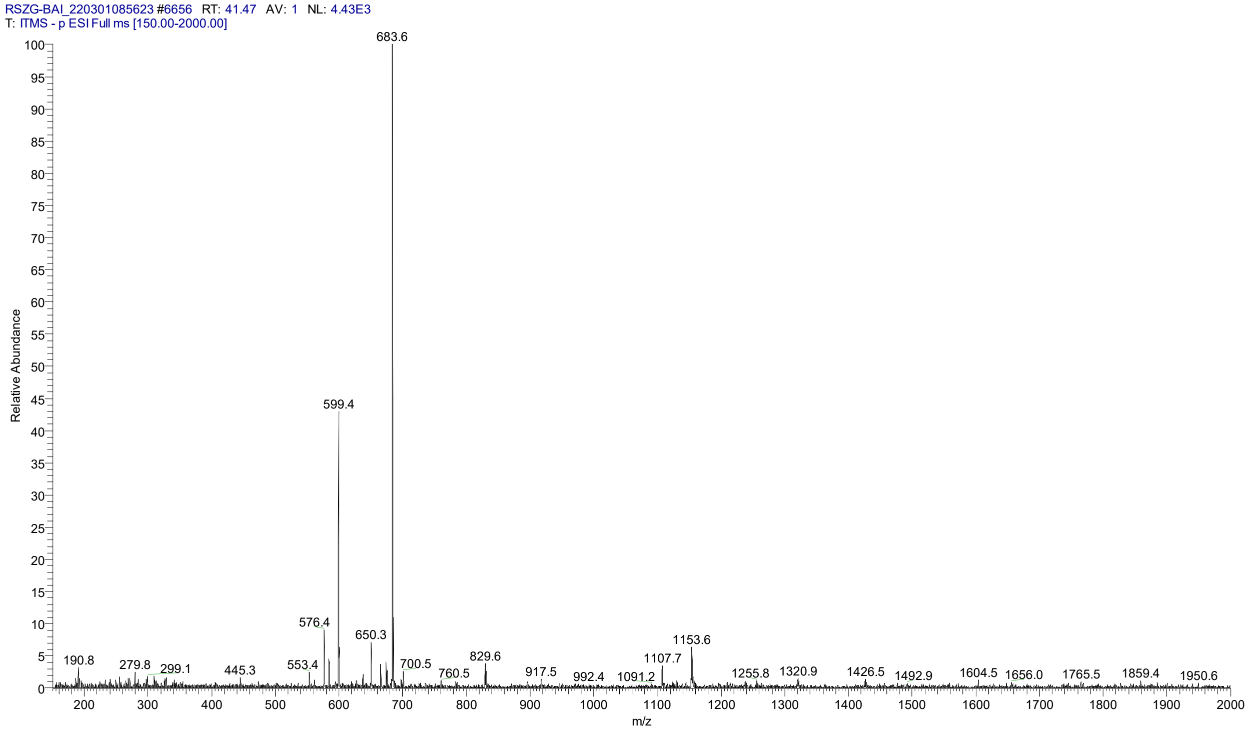
RSZG-BAI_220301085623 #6656 RT: 41.47 AV: 1 NL: 4.43E3
T: ITMS - p ESI Full ms [150.00-2000.00]
Relative Abundance
m/z
683.6
599.4
576.4
650.3
1153.6
1107.7
829.6
700.5
553.4
445.3
299.1
279.8
190.8
760.5
917.5
992.4
1091.2
1255.8
1320.9
1426.5
1492.9
1604.5
1656.0
1765.5
1859.4
1950.6

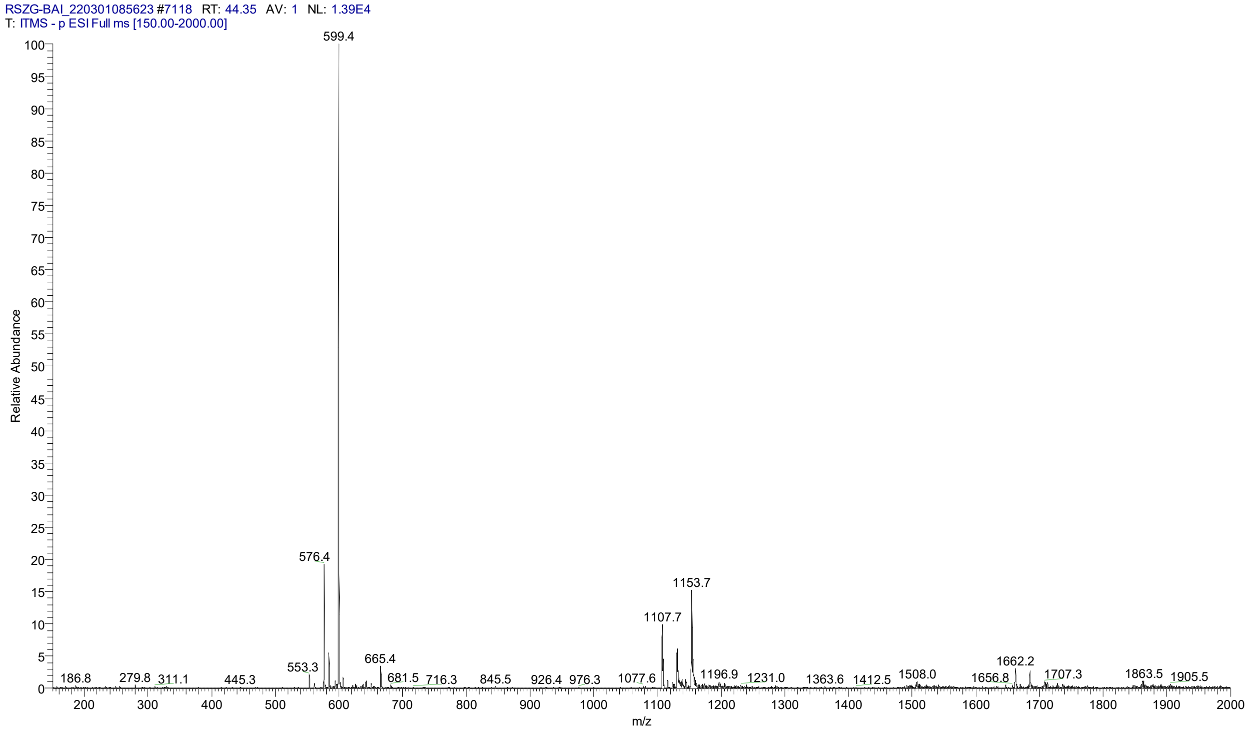
RSZG-BAI_220301085623 #7118 RT: 44.35 AV: 1 NL: 1.39E4
T: ITMS - p ESI Full ms [150.00-2000.00]
Relative Abundance
m/z
599.4
576.4
1153.7
1107.7
665.4
1662.2
553.3
186.8
279.8
311.1
445.3
681.5
716.3
845.5
926.4
976.3
1077.6
1196.9
1231.0
1363.6
1412.5
1508.0
1656.8
1707.3
1863.5
1905.5

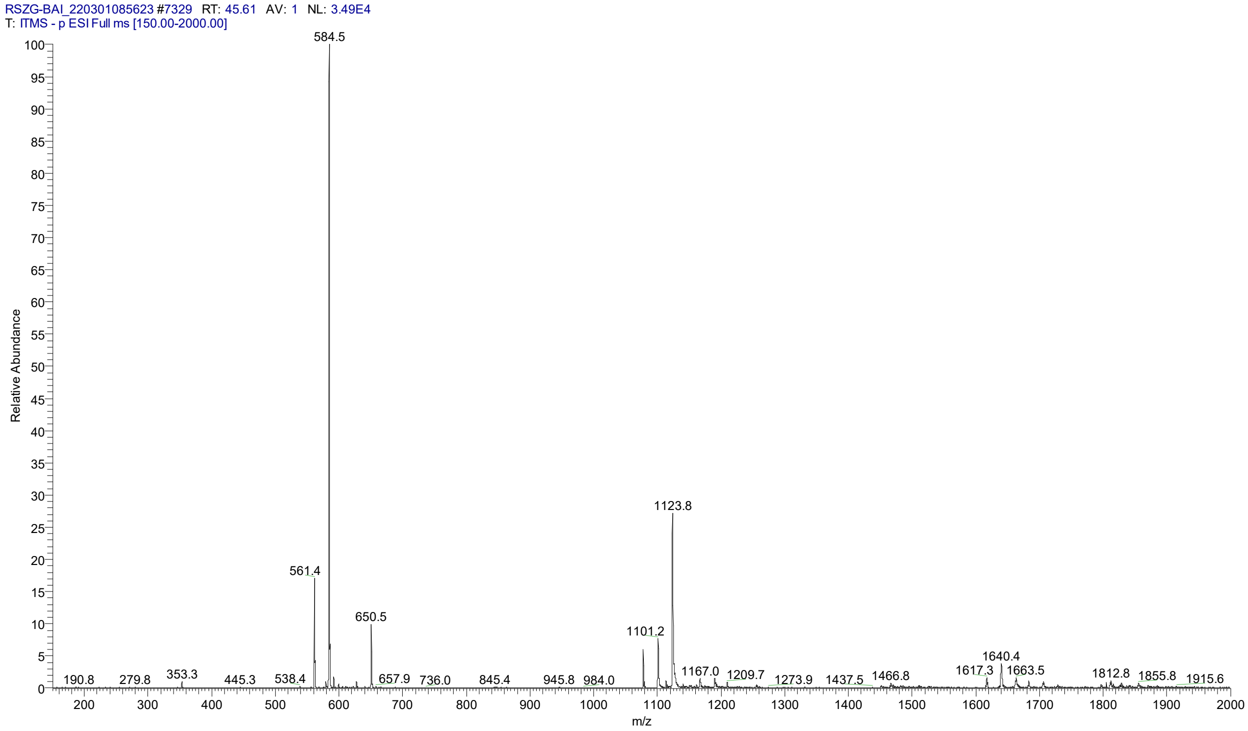
RSZG-BAI_220301085623 #7329 RT: 45.61 AV: 1 NL: 3.49E4
T: ITMS - p ESI Full ms [150.00-2000.00]
Relative Abundance
m/z
584.5
1123.8
561.4
650.5
1101.2
190.8
279.8
353.3
445.3
538.4
657.9
736.0
845.4
945.8
984.0
1167.0
1209.7
1273.9
1437.5
1466.8
1617.3
1640.4
1663.5
1812.8
1855.8
1915.6

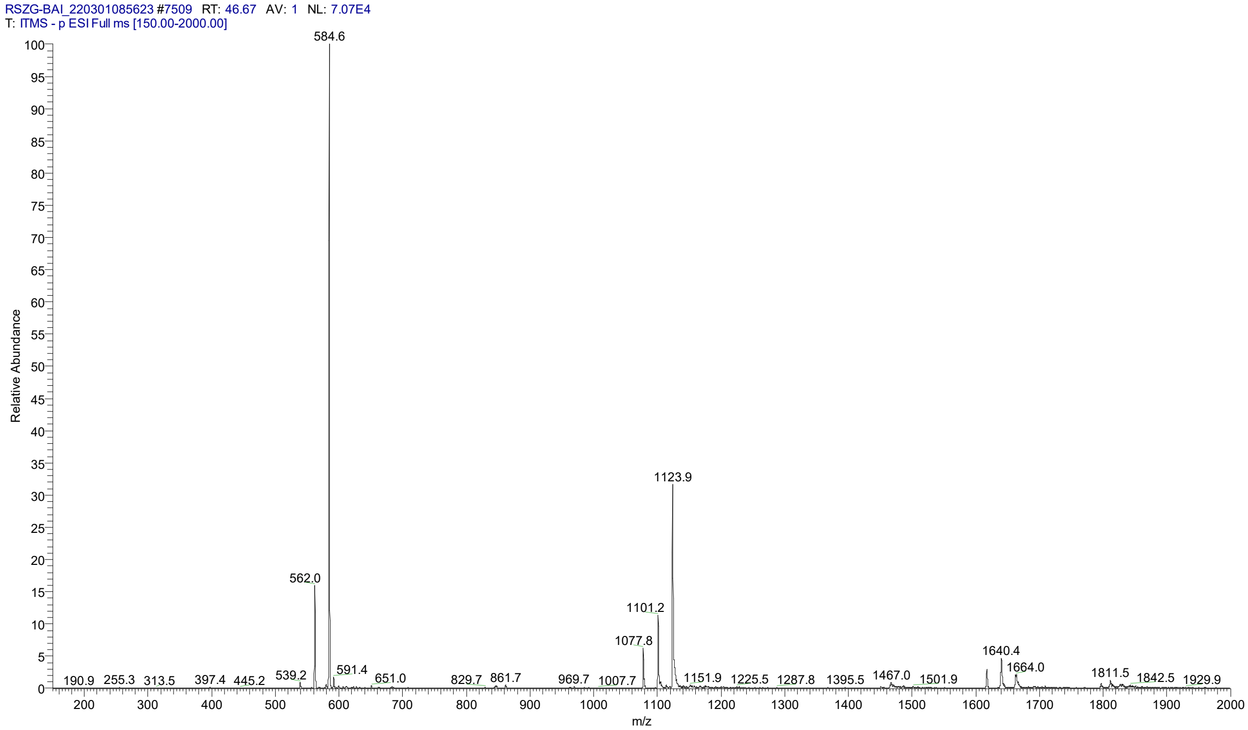
RSZG-BAI_220301085623 #7509 RT: 46.67 AV: 1 NL: 7.07E4
T: ITMS - p ESI Full ms [150.00-2000.00]
Relative Abundance
m/z
584.6
1123.9
562.0
1101.2
1077.8
190.9
255.3
313.5
397.4
445.2
539.2
591.4
651.0
829.7
861.7
969.7
1007.7
1151.9
1225.5
1287.8
1395.5
1467.0
1501.9
1640.4
1664.0
1811.5
1842.5
1929.9

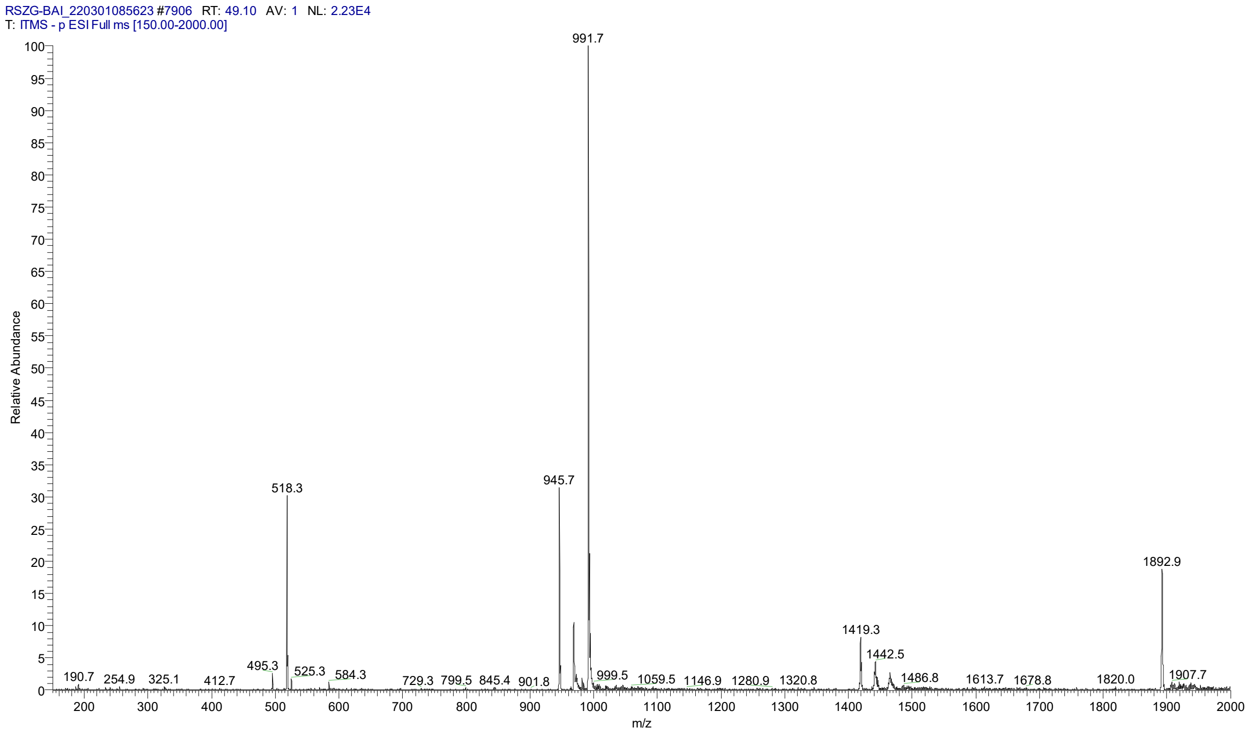
RSZG-BAI_220301085623 #7906 RT: 49.10 AV: 1 NL: 2.23E4
T: ITMS - p ESI Full ms [150.00-2000.00]
Relative Abundance
m/z
991.7
518.3
945.7
1892.9
1419.3
1442.5
190.7
254.9
325.1
412.7
495.3
525.3
584.3
729.3
799.5
845.4
901.8
999.5
1059.5
1146.9
1280.9
1320.8
1486.8
1613.7
1678.8
1820.0
1907.7

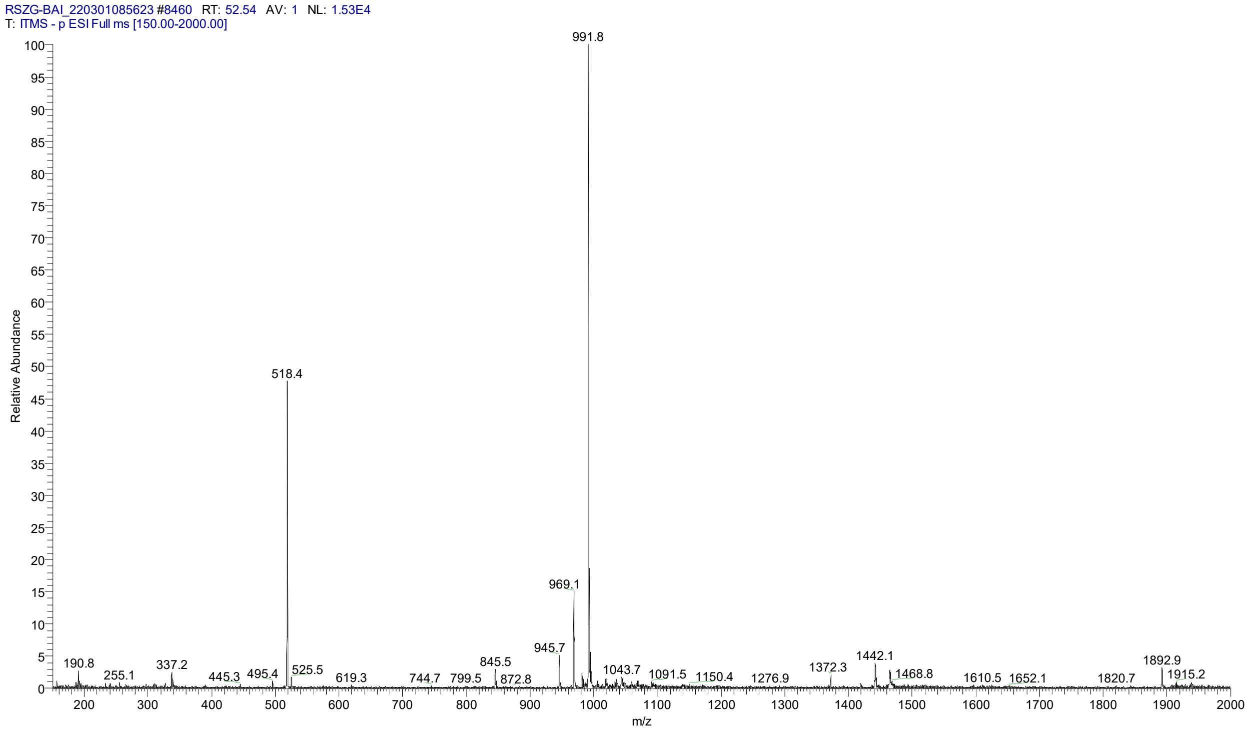
RSZG-BAI_220301085623 #8460 RT: 52.54 AV: 1 NL: 1.53E4
T: ITMS - p ESI Full ms [150.00-2000.00]
Relative Abundance
m/z
991.8
518.4
969.1
945.7
845.5
190.8
255.1
337.2
445.3
495.4
525.5
619.3
744.7
799.5
872.8
1043.7
1091.5
1150.4
1276.9
1372.3
1442.1
1468.8
1610.5
1652.1
1820.7
1892.9
1915.2

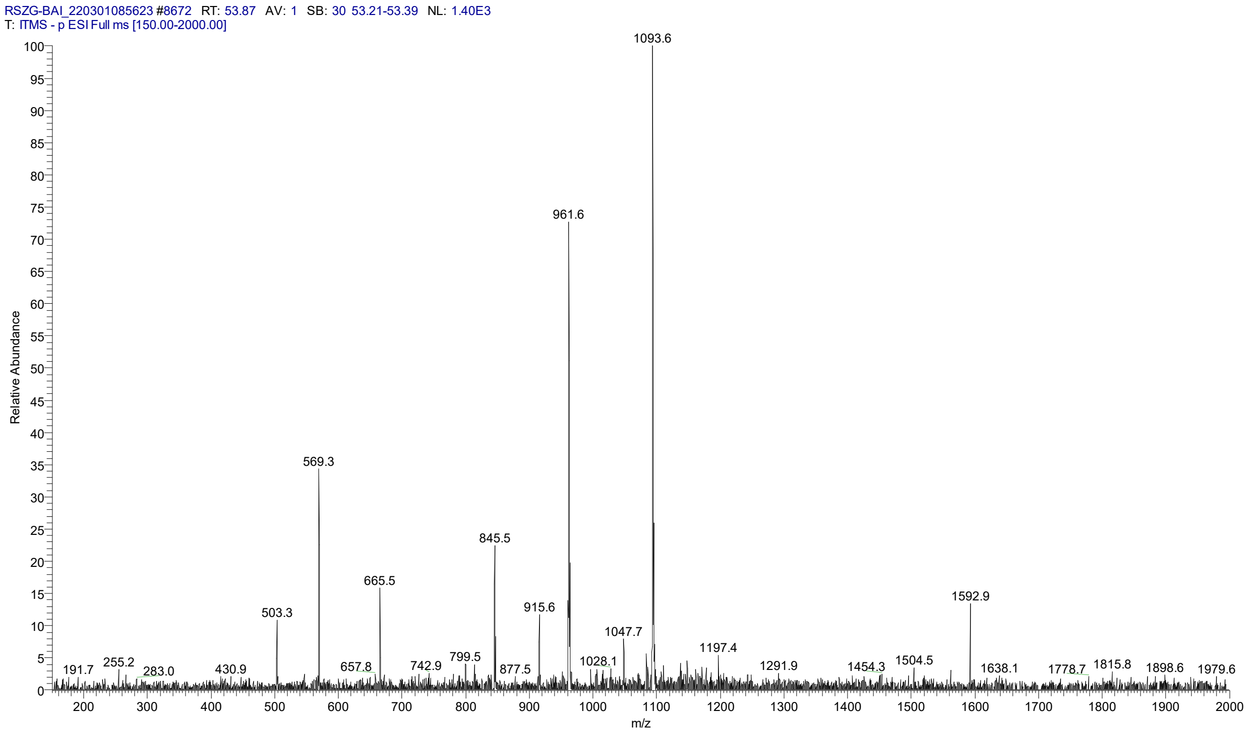
RSZG-BAI_220301085623 #8672 RT: 53.87 AV: 1 SB: 30 53.21-53.39 NL: 1.40E3
T: ITMS - p ESI Full ms [150.00-2000.00]
Relative Abundance
m/z
1093.6
961.6
569.3
845.5
665.5
503.3
915.6
1592.9
1047.7
1197.4
191.7
255.2
283.0
430.9
657.8
742.9
799.5
877.5
1028.1
1291.9
1454.3
1504.5
1638.1
1778.7
1815.8
1898.6
1979.6

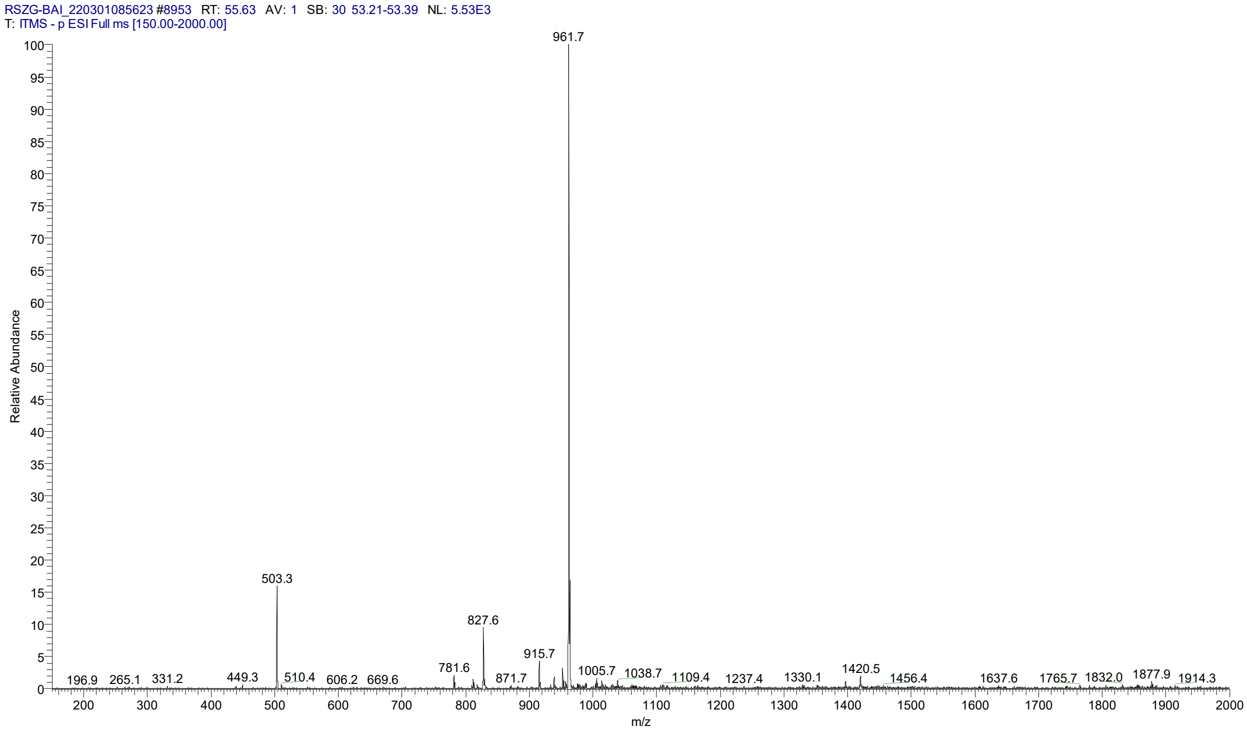
RSZG-BAI_220301085623 #8953 RT: 55.63 AV: 1 SB: 30 53.21-53.39 NL: 5.53E3
T: ITMS - p ESI Full ms [150.00-2000.00]
Relative Abundance
m/z
961.7
503.3
827.6
915.7
781.6
196.9
265.1
331.2
449.3
510.4
606.2
669.6
871.7
1005.7
1038.7
1109.4
1237.4
1330.1
1420.5
1456.4
1637.6
1765.7
1832.0
1877.9
1914.3

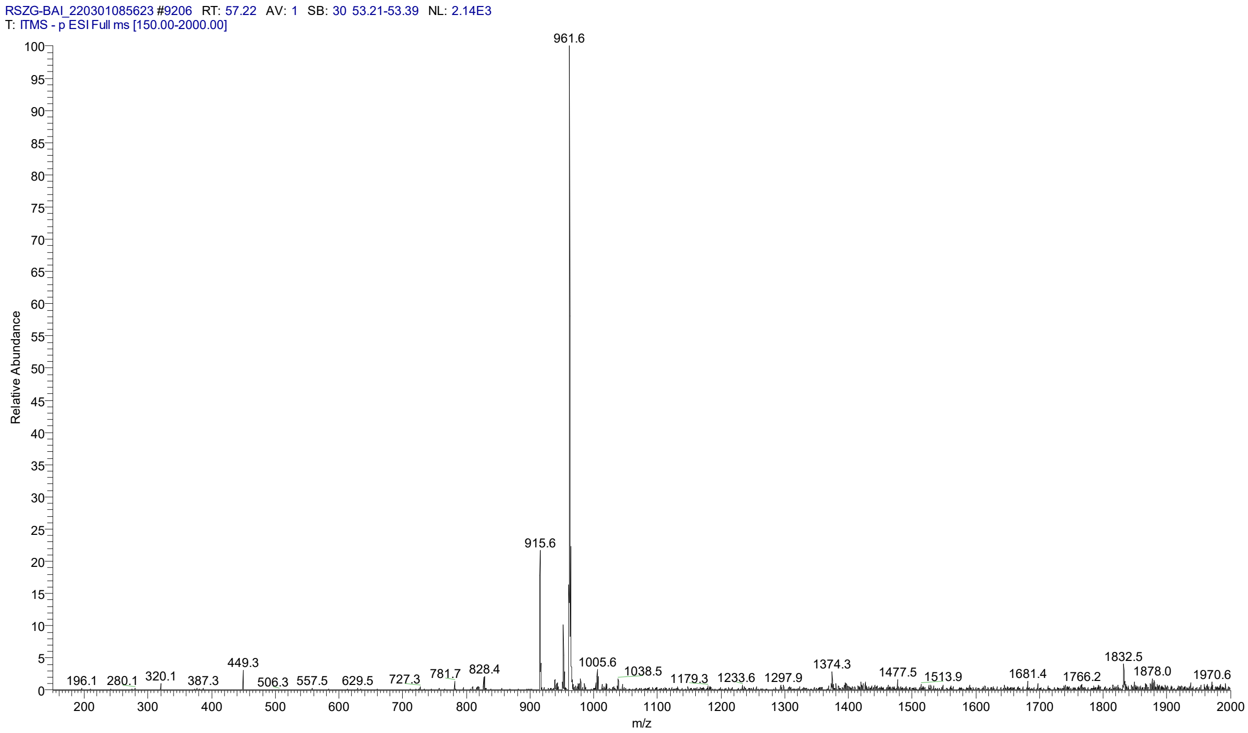

RSZG-BAI_220301085623 #9206 RT: 57.22 AV: 1 SB: 30 53.21-53.39 NL: 2.14E3
T: ITMS - p ESI Full ms [150.00-2000.00]
Relative Abundance
961.6
915.6
196.1
280.1
320.1
387.3
449.3
506.3
557.5
629.5
727.3
781.7
828.4
1005.6
1038.5
1179.3
1233.6
1297.9
1374.3
1477.5
1513.9
1681.4
1766.2
1832.5
1878.0
1970.6
m/z

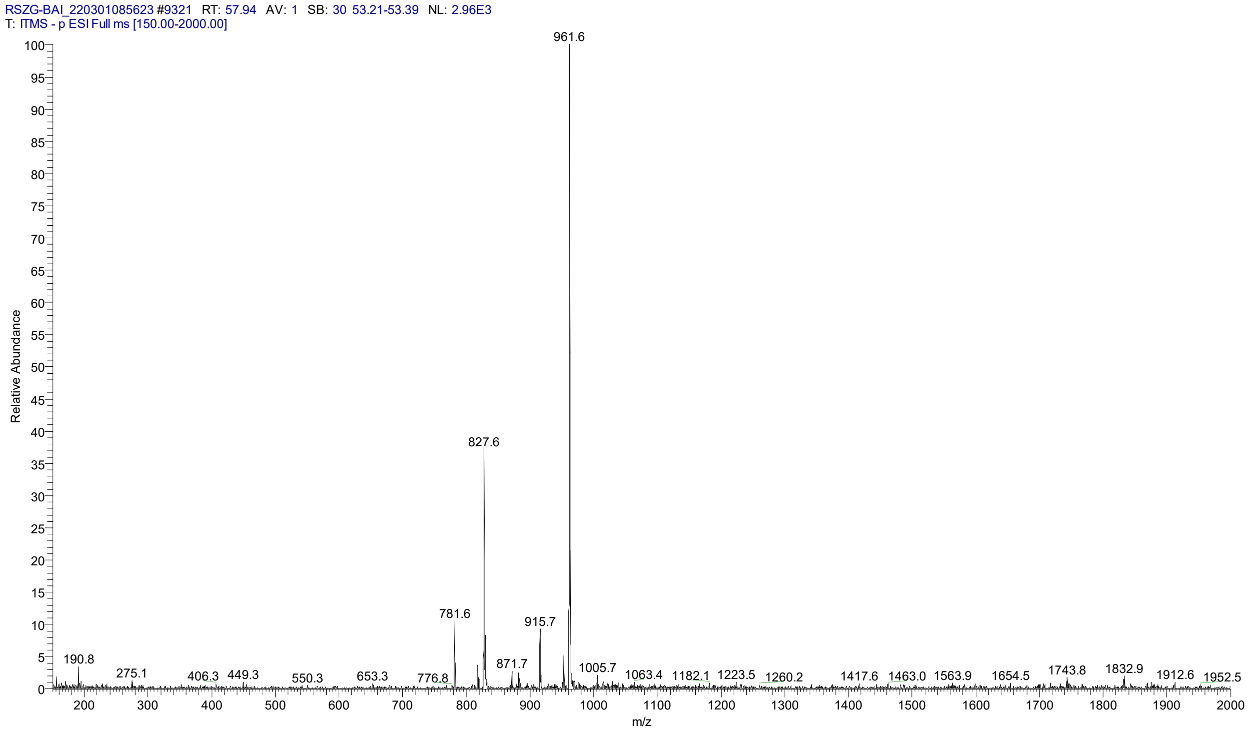

RSZG-BAI_220301085623 #9321 RT: 57.94 AV: 1 SB: 30 53.21-53.39 NL: 2.96E3
T: ITMS - p ESI Full ms [150.00-2000.00]
Relative Abundance
961.6
827.6
781.6
915.7
190.8
275.1
406.3
449.3
550.3
653.3
776.8
871.7
1005.7
1063.4
1182.1
1223.5
1260.2
1417.6
1463.0
1563.9
1654.5
1743.8
1832.9
1912.6
1952.5
m/z

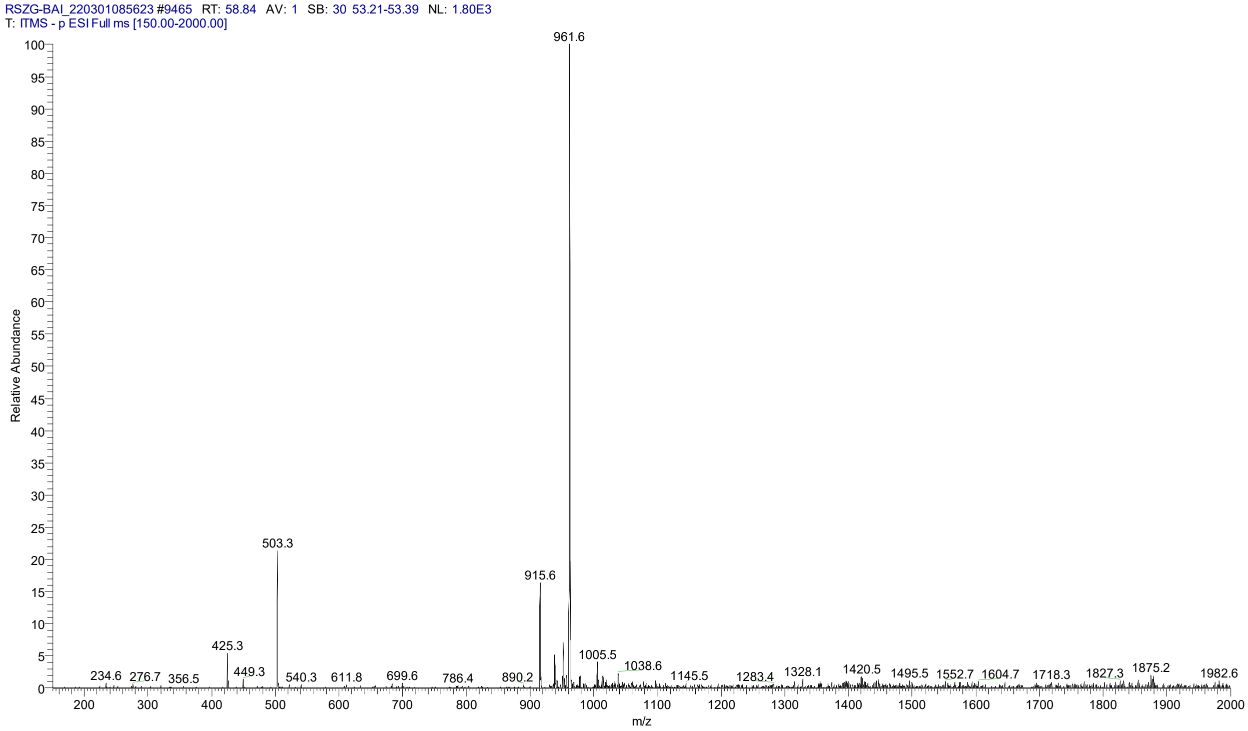
RSZG-BAI_220301085623 #9465 RT: 58.84 AV: 1 SB: 30 53.21-53.39 NL: 1.80E3
T: ITMS - p ESI Full ms [150.00-2000.00]
Relative Abundance
m/z
961.6
503.3
915.6
425.3
1005.5
1038.6

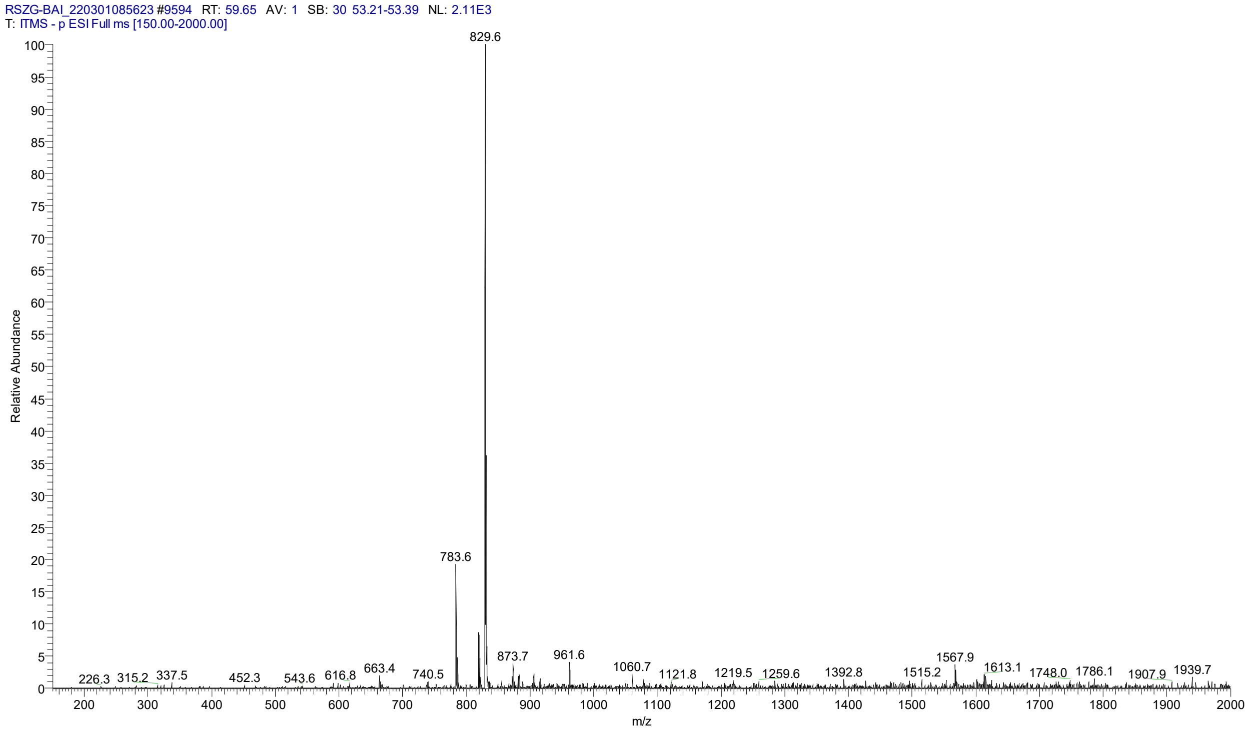
RSZG-BAI_220301085623 #9594 RT: 59.65 AV: 1 SB: 30 53.21-53.39 NL: 2.11E3
T: ITMS - p ESI Full ms [150.00-2000.00]
Relative Abundance
m/z
829.6
783.6
873.7
961.6
1567.9

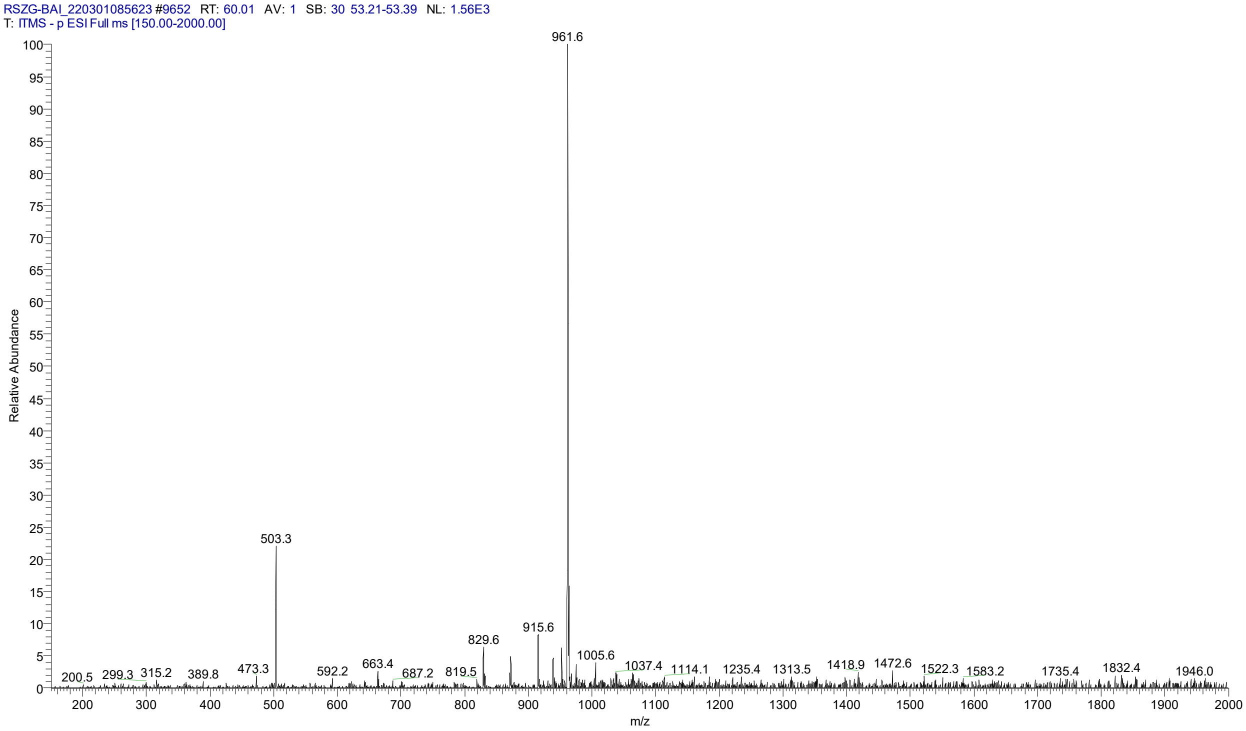
RSZG-BAI_220301085623 #9652 RT: 60.01 AV: 1 SB: 30 53.21-53.39 NL: 1.56E3
T: ITMS - p ESI Full ms [150.00-2000.00]
Relative Abundance
m/z
961.6
503.3
915.6
829.6
1005.6
1037.4
1114.1
1235.4
1313.5
1418.9
1472.6
1522.3
1583.2
1735.4
1832.4
1946.0
200.5
299.3
315.2
389.8
473.3
592.2
663.4
687.2
819.5

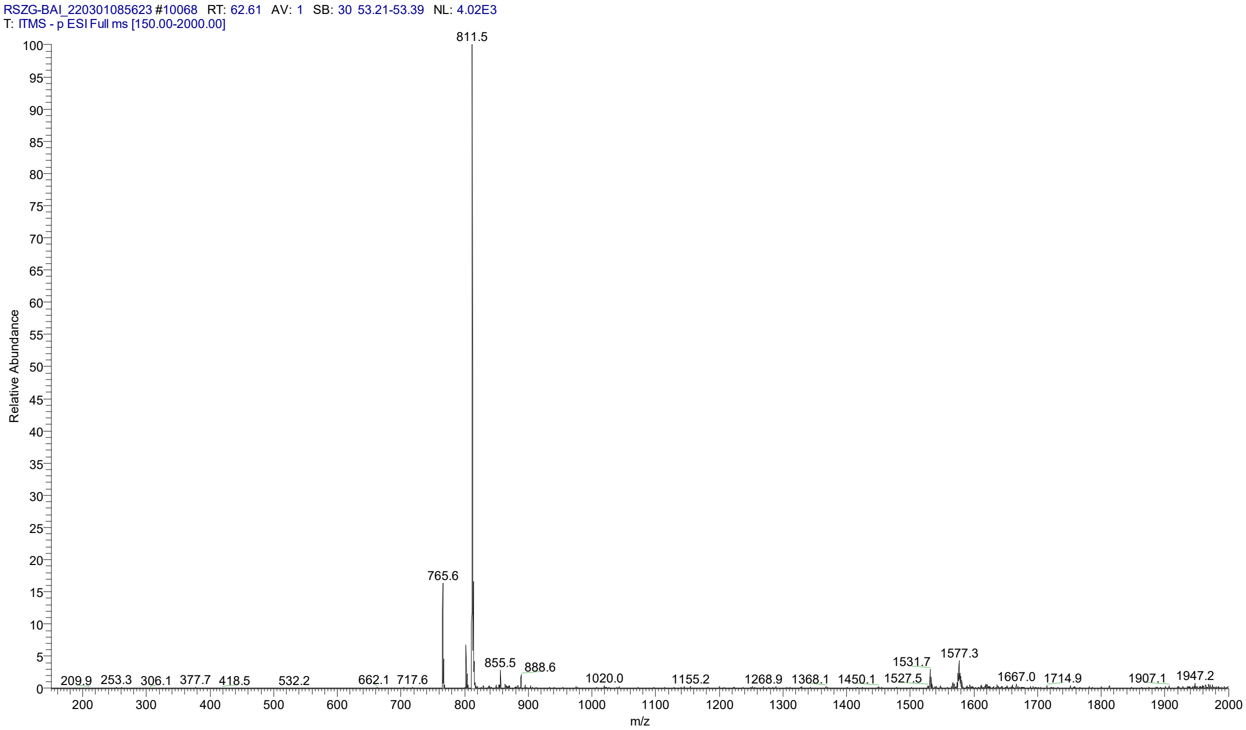
RSZG-BAI_220301085623 #10068 RT: 62.61 AV: 1 SB: 30 53.21-53.39 NL: 4.02E3
T: ITMS - p ESI Full ms [150.00-2000.00]
Relative Abundance
m/z
811.5
765.6
855.5
888.6
1020.0
1155.2
1268.9
1368.1
1450.1
1527.5
1531.7
1577.3
1667.0
1714.9
1907.1
1947.2
209.9
253.3
306.1
377.7
418.5
532.2
662.1
717.6

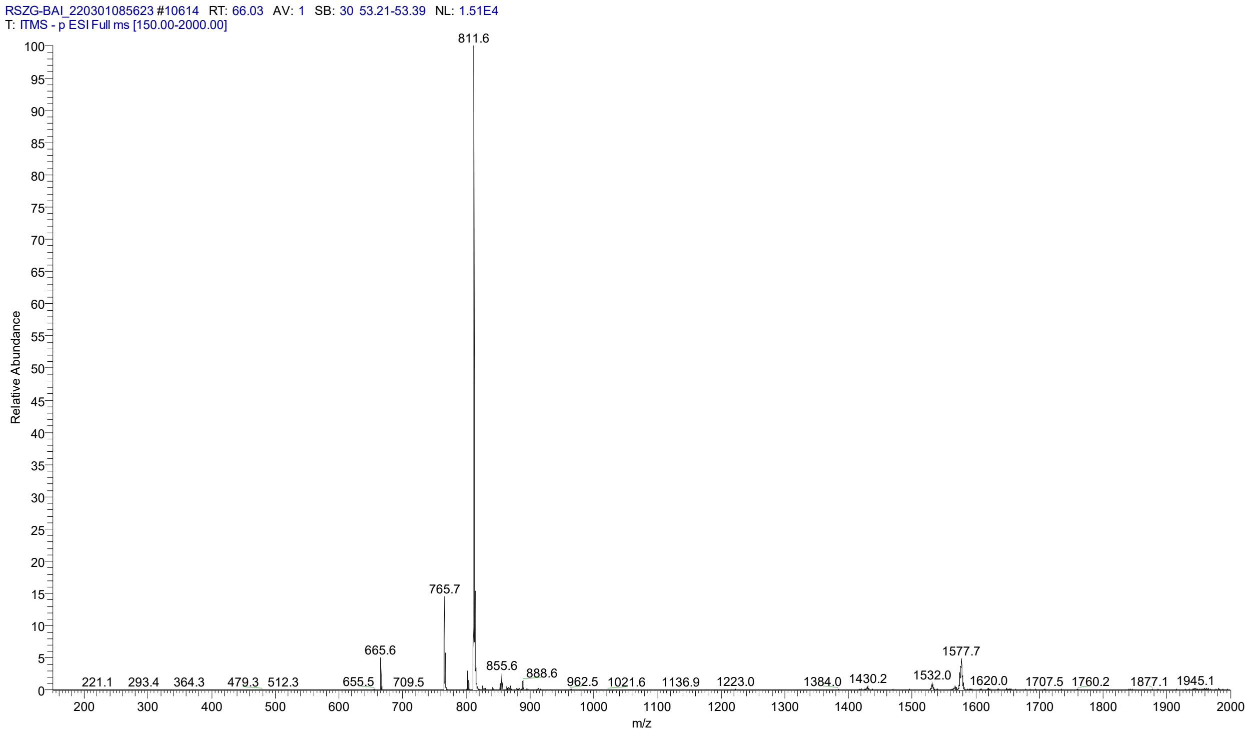
RSZG-BAI_220301085623 #10614 RT: 66.03 AV: 1 SB: 30 53.21-53.39 NL: 1.51E4
T: ITMS - p ESI Full ms [150.00-2000.00]
Relative Abundance
m/z
811.6
765.7
665.6
855.6
888.6
1577.7
221.1
293.4
364.3
479.3
512.3
655.5
709.5
962.5
1021.6
1136.9
1223.0
1384.0
1430.2
1532.0
1620.0
1707.5
1760.2
1877.1
1945.1

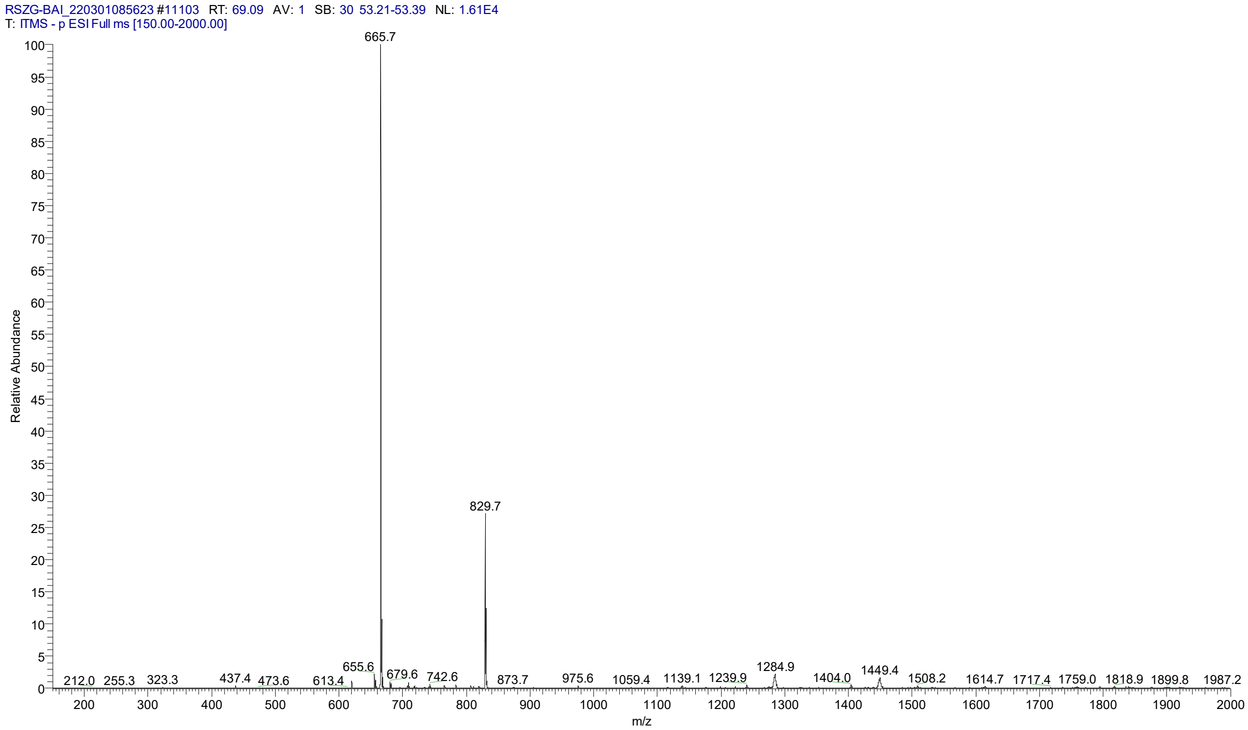
RSZG-BAI_220301085623 #11103 RT: 69.09 AV: 1 SB: 30 53.21-53.39 NL: 1.61E4
T: ITMS - p ESI Full ms [150.00-2000.00]
Relative Abundance
m/z
665.7
829.7
655.6
679.6
742.6
1284.9
1449.4
212.0
255.3
323.3
437.4
473.6
613.4
873.7
975.6
1059.4
1139.1
1239.9
1404.0
1508.2
1614.7
1717.4
1759.0
1818.9
1899.8
1987.2

RSZG-BAI_220301085623 #12382 RT: 77.10 AV: 1 SB: 30 53.21-53.39 NL: 3.81E3
T: ITMS - p ESI Full ms [150.00-2000.00]

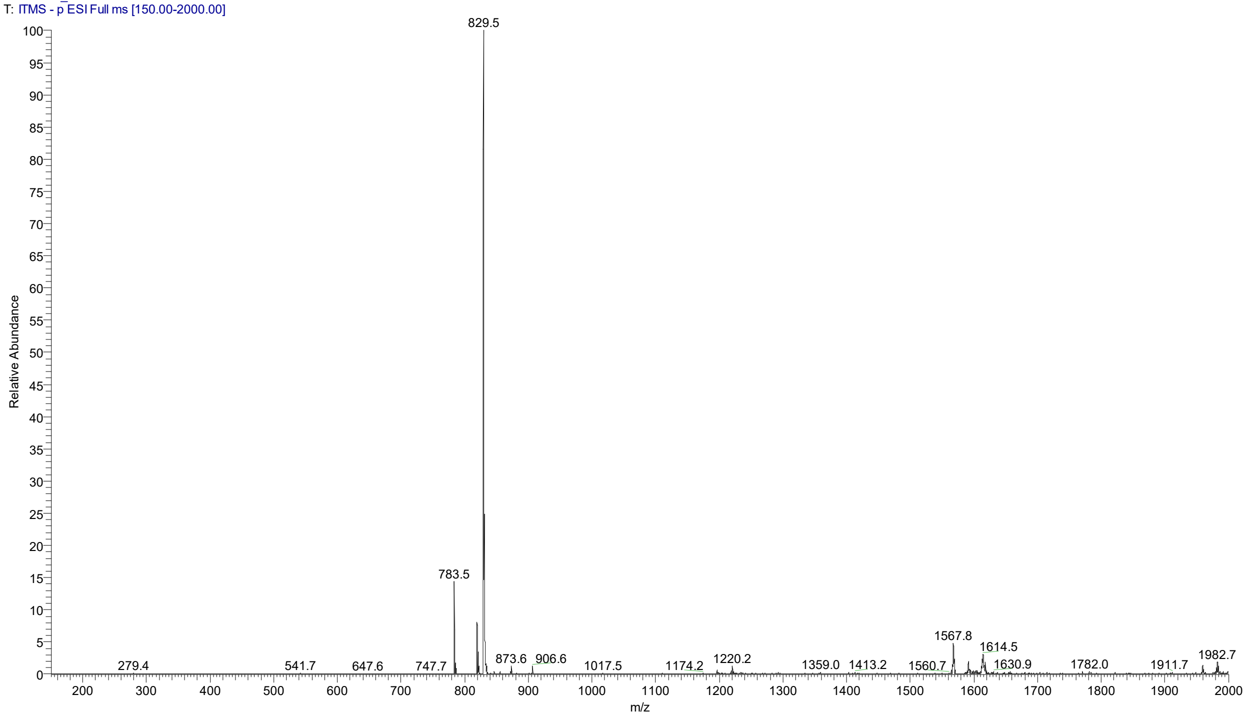

RSZG-BAI_220301085623 #12569 RT: 78.27 AV: 1 SB: 30 53.21-53.39 NL: 5.75E3
T: ITMS - p ESI Full ms [150.00-2000.00]

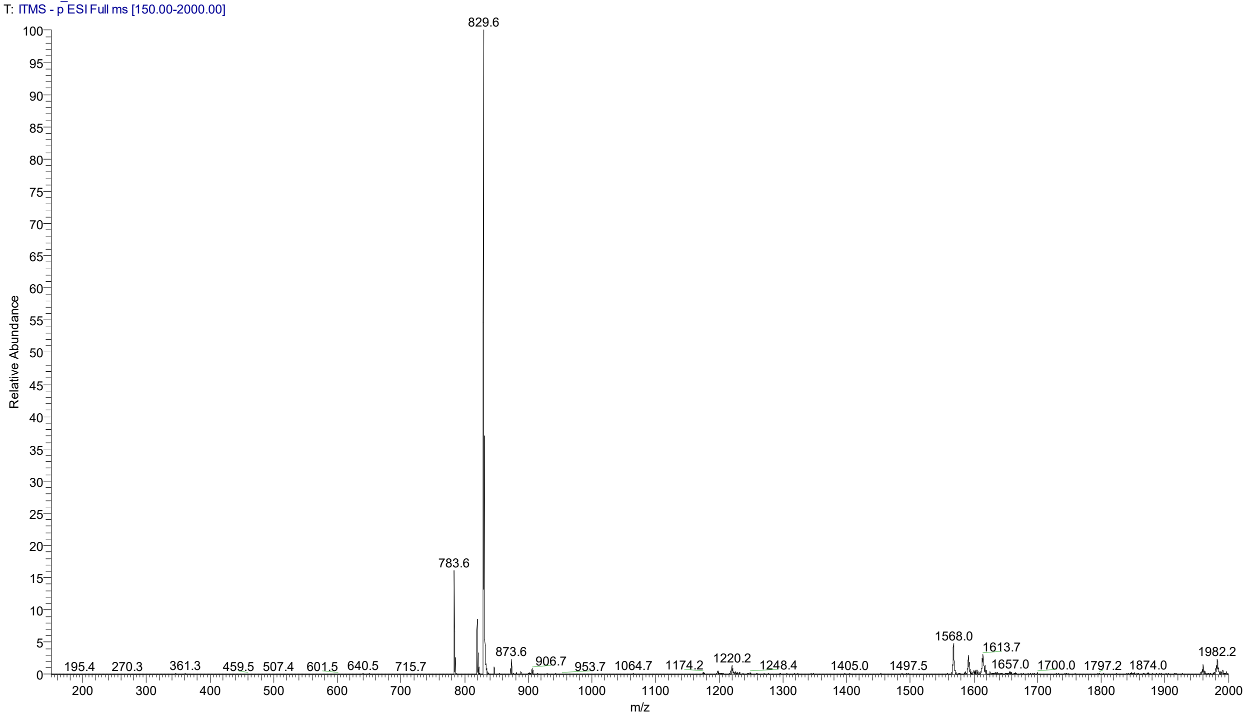

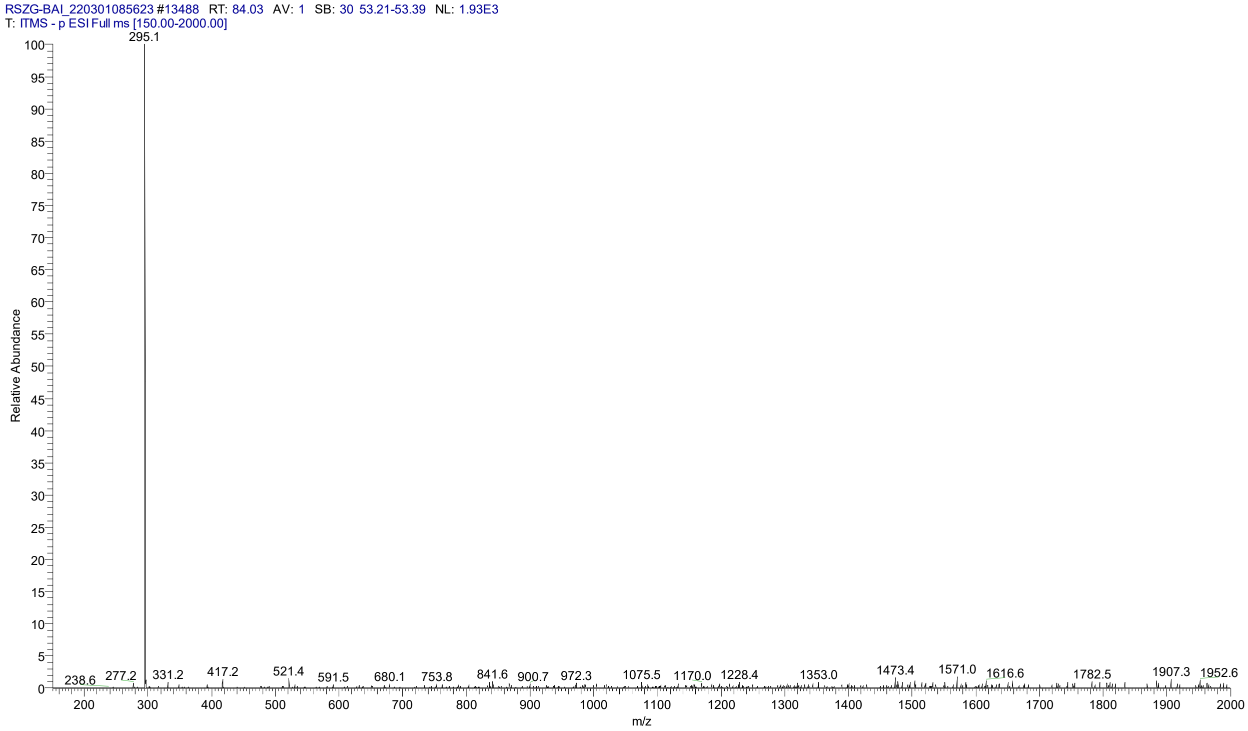
RSZG-BAI_220301085623 #13488 RT: 84.03 AV: 1 SB: 30 53.21-53.39 NL: 1.93E3
T: ITMS - p ESI Full ms [150.00-2000.00]
Relative Abundance
m/z
295.1
238.6
277.2
331.2
417.2
521.4
591.5
680.1
753.8
841.6
900.7
972.3
1075.5
1170.0
1228.4
1353.0
1473.4
1571.0
1616.6
1782.5
1907.3
1952.6

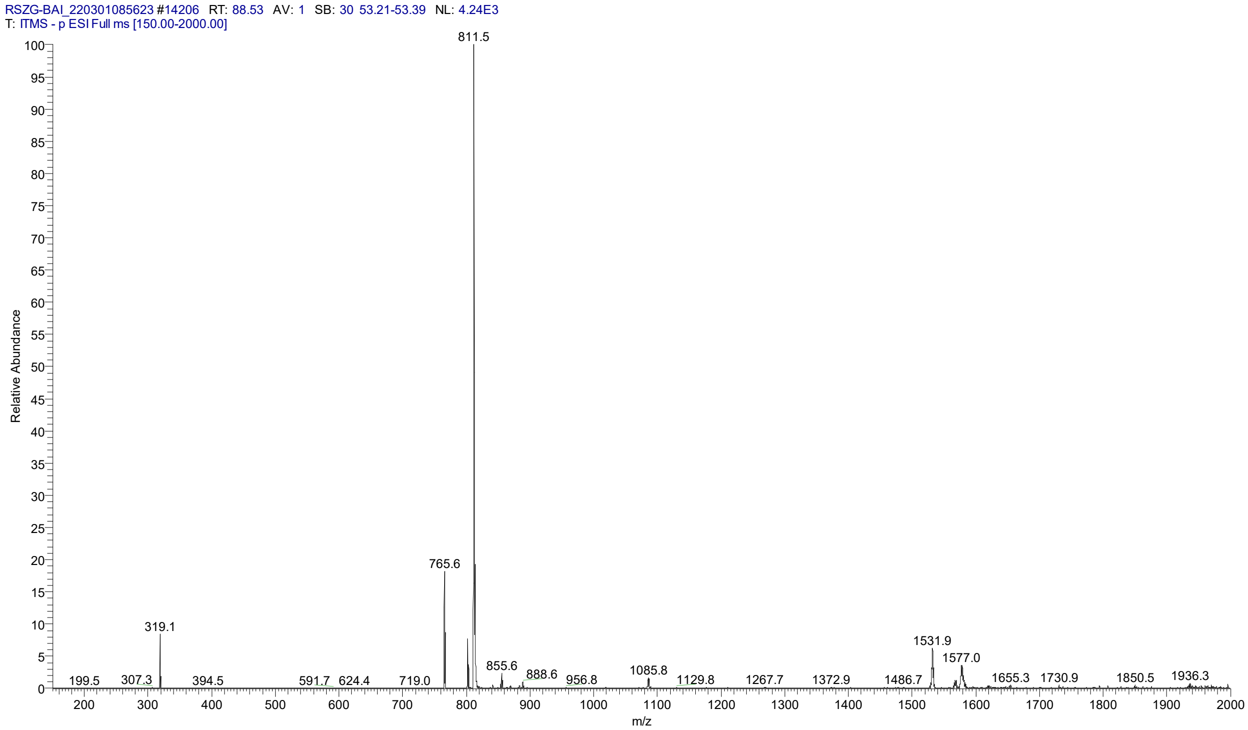
RSZG-BAI_220301085623 #14206 RT: 88.53 AV: 1 SB: 30 53.21-53.39 NL: 4.24E3
T: ITMS - p ESI Full ms [150.00-2000.00]
Relative Abundance
m/z
811.5
765.6
319.1
199.5
307.3
394.5
591.7
624.4
719.0
855.6
888.6
956.8
1085.8
1129.8
1267.7
1372.9
1486.7
1531.9
1577.0
1655.3
1730.9
1850.5
1936.3

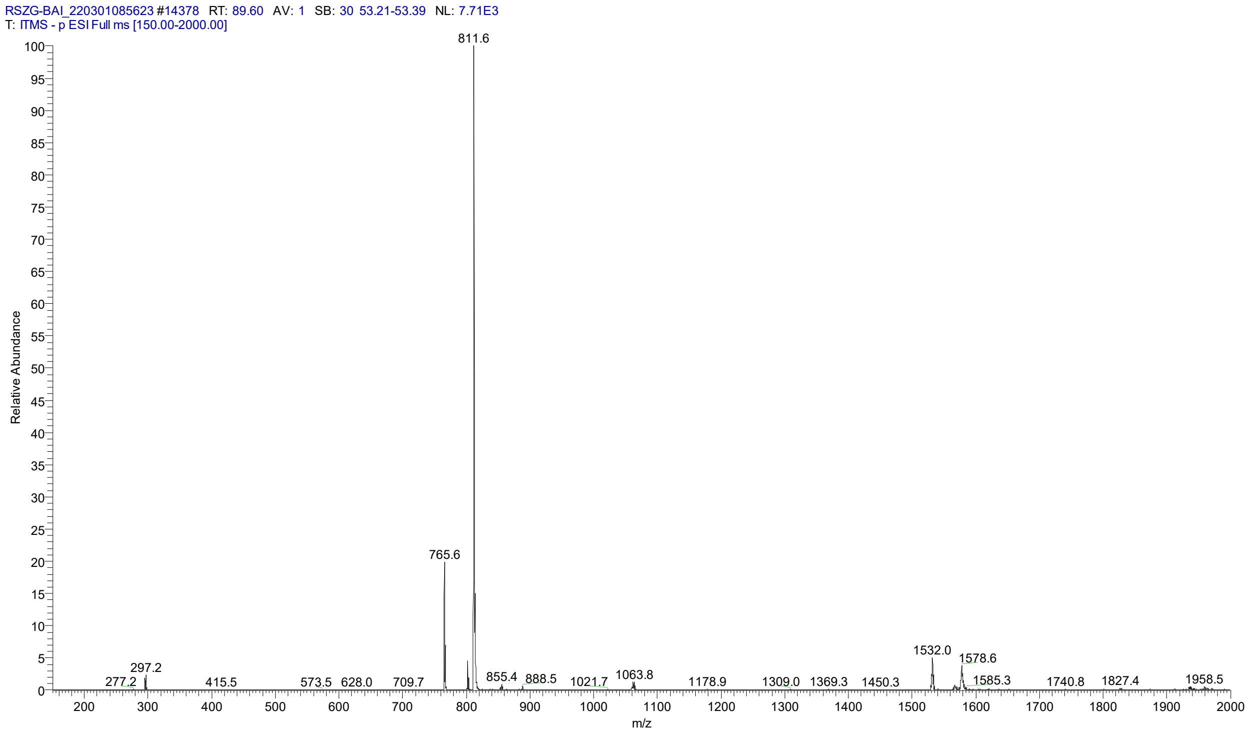
RSZG-BAI_220301085623 #14378 RT: 89.60 AV: 1 SB: 30 53.21-53.39 NL: 7.71E3
T: ITMS - p ESI Full ms [150.00-2000.00]
Relative Abundance
m/z
811.6
765.6
297.2
277.2
415.5
573.5
628.0
709.7
855.4
888.5
1021.7
1063.8
1178.9
1309.0
1369.3
1450.3
1532.0
1578.6
1585.3
1740.8
1827.4
1958.5

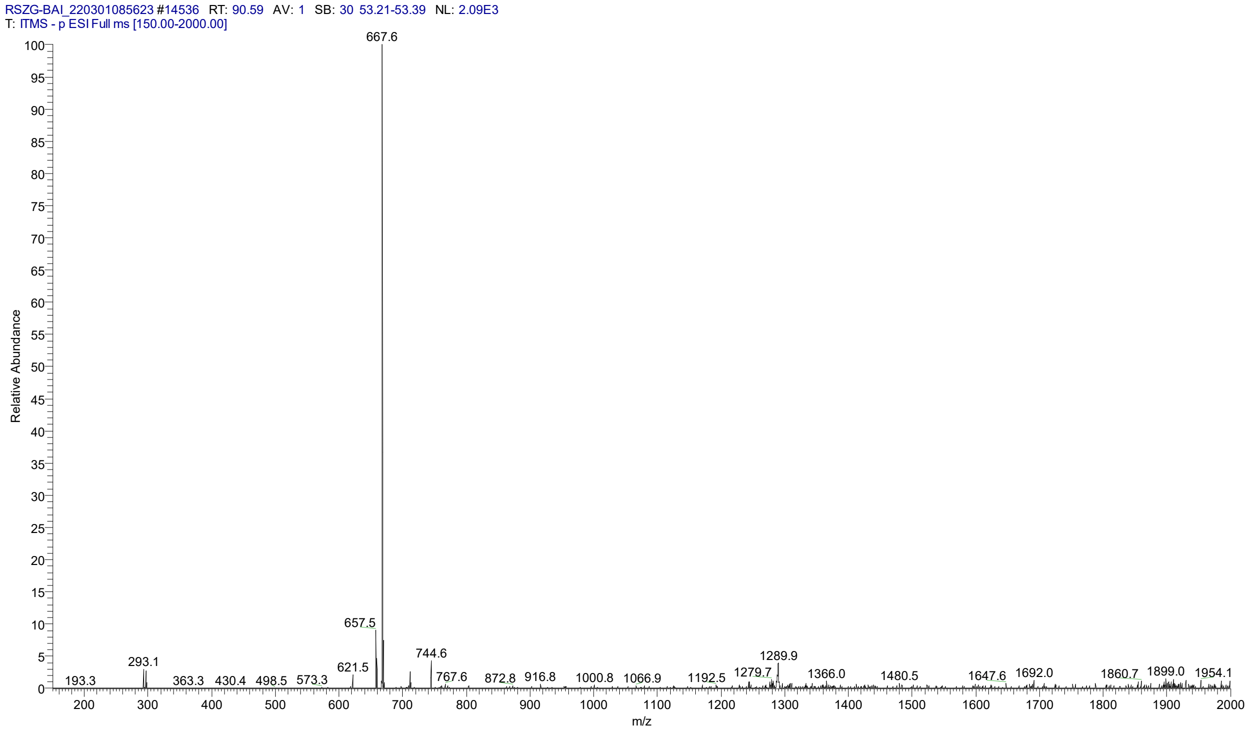
RSZG-BAI_220301085623 #14536 RT: 90.59 AV: 1 SB: 30 53.21-53.39 NL: 2.09E3
T: ITMS - p ESI Full ms [150.00-2000.00]
Relative Abundance
m/z
667.6
657.5
744.6
621.5
293.1
193.3
363.3
430.4
498.5
573.3
767.6
872.8
916.8
1000.8
1066.9
1192.5
1279.7
1289.9
1366.0
1480.5
1647.6
1692.0
1860.7
1899.0
1954.1

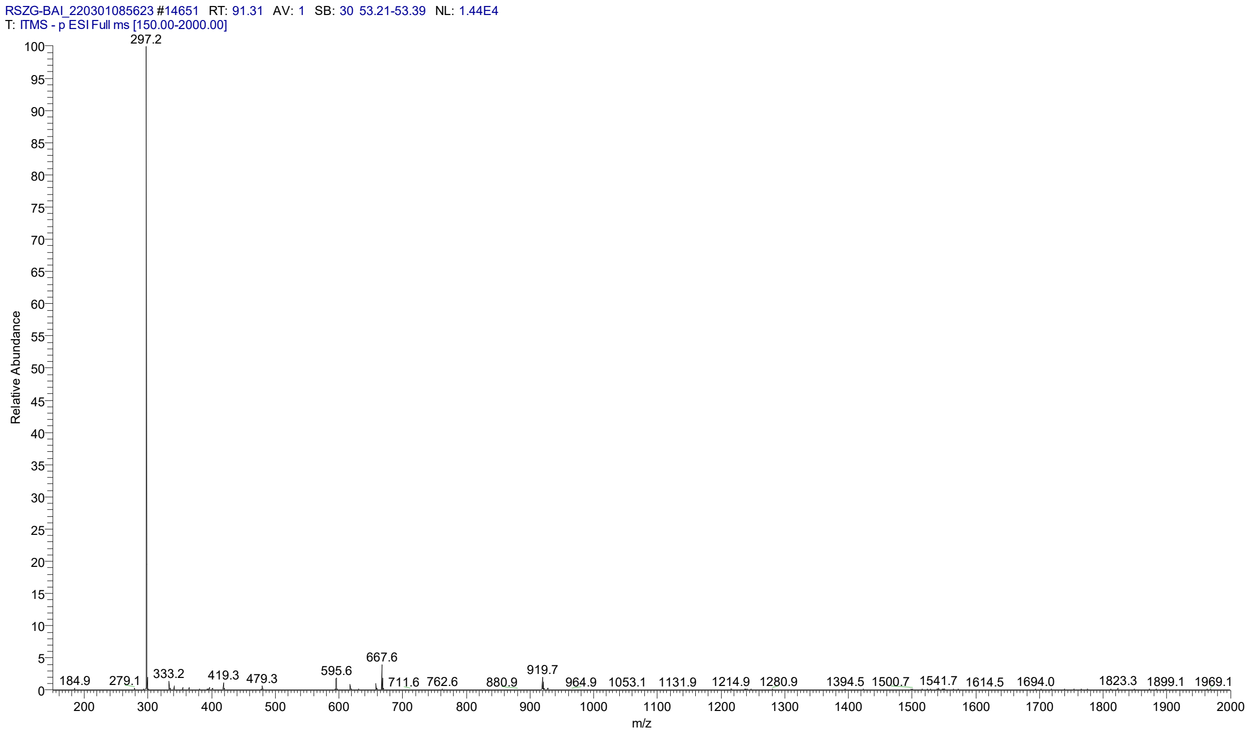
RSZG-BAI_220301085623 #14651 RT: 91.31 AV: 1 SB: 30 53.21-53.39 NL: 1.44E4
T: ITMS - p ESI Full ms [150.00-2000.00]
297.2
333.2
419.3
479.3
595.6
667.6
711.6
762.6
880.9
919.7
964.9
1053.1
1131.9
1214.9
1280.9
1394.5
1500.7
1541.7
1614.5
1694.0
1823.3
1899.1
1969.1
184.9
279.1
Relative Abundance
m/z

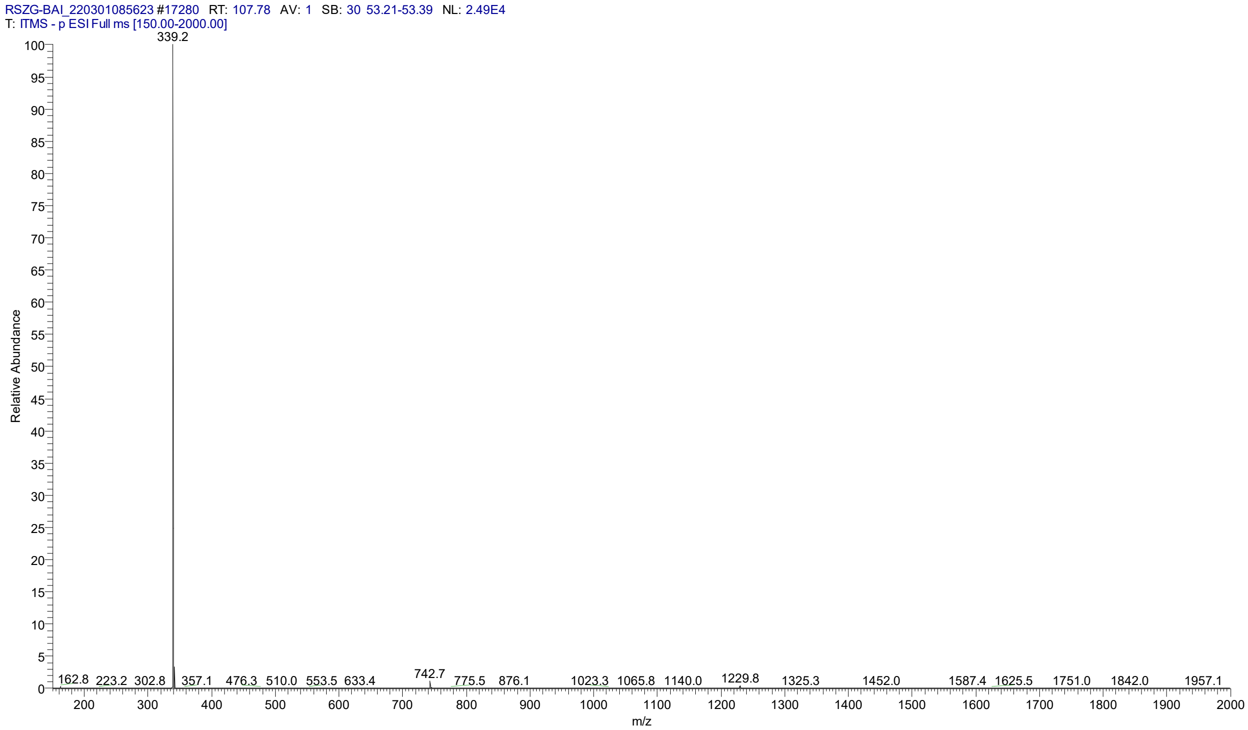
RSZG-BAI_220301085623 #17280 RT: 107.78 AV: 1 SB: 30 53.21-53.39 NL: 2.49E4
T: ITMS - p ESI Full ms [150.00-2000.00]
339.2
162.8
223.2
302.8
357.1
476.3
510.0
553.5
633.4
742.7
775.5
876.1
1023.3
1065.8
1140.0
1229.8
1325.3
1452.0
1587.4
1625.5
1751.0
1842.0
1957.1
Relative Abundance
m/z

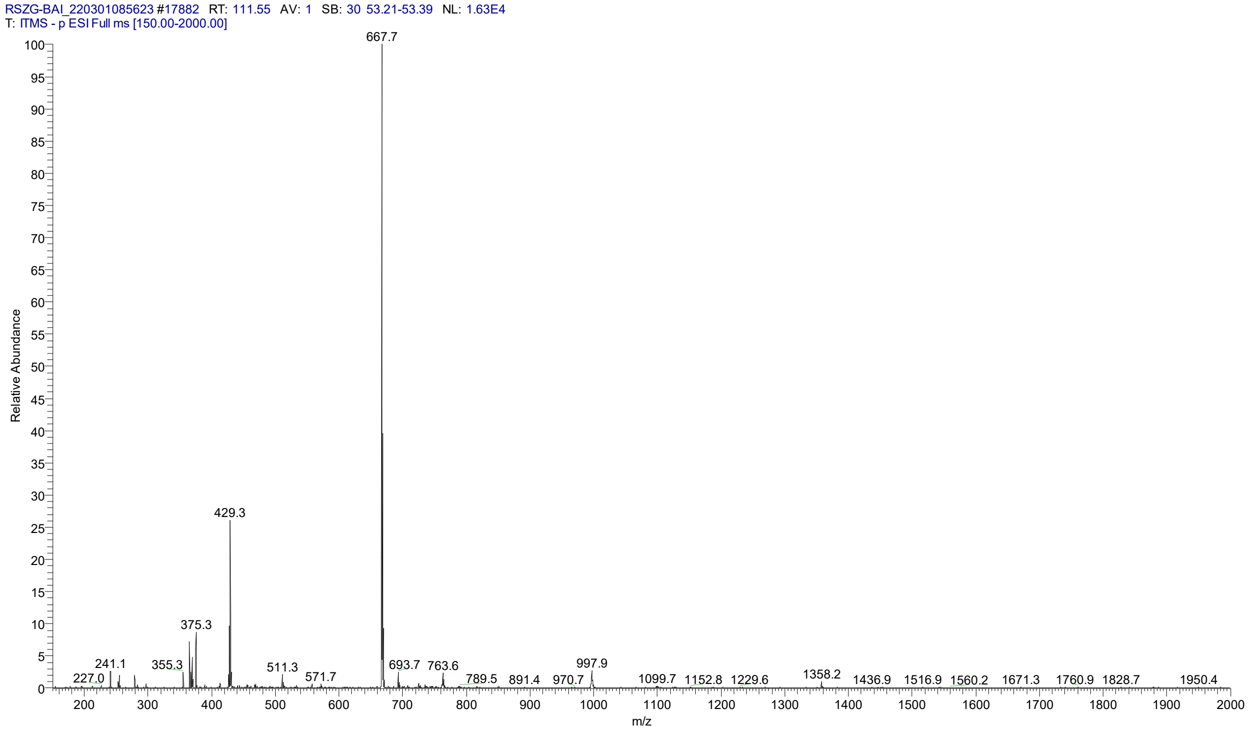
RSZG-BAI_220301085623 #17882 RT: 111.55 AV: 1 SB: 30 53.21-53.39 NL: 1.63E4
T: ITMS - p ESI Full ms [150.00-2000.00]
Relative Abundance
667.7
429.3
375.3
355.3
241.1
227.0
511.3
571.7
693.7
763.6
789.5
891.4
970.7
997.9
1099.7
1152.8
1229.6
1358.2
1436.9
1516.9
1560.2
1671.3
1760.9
1828.7
1950.4
m/z

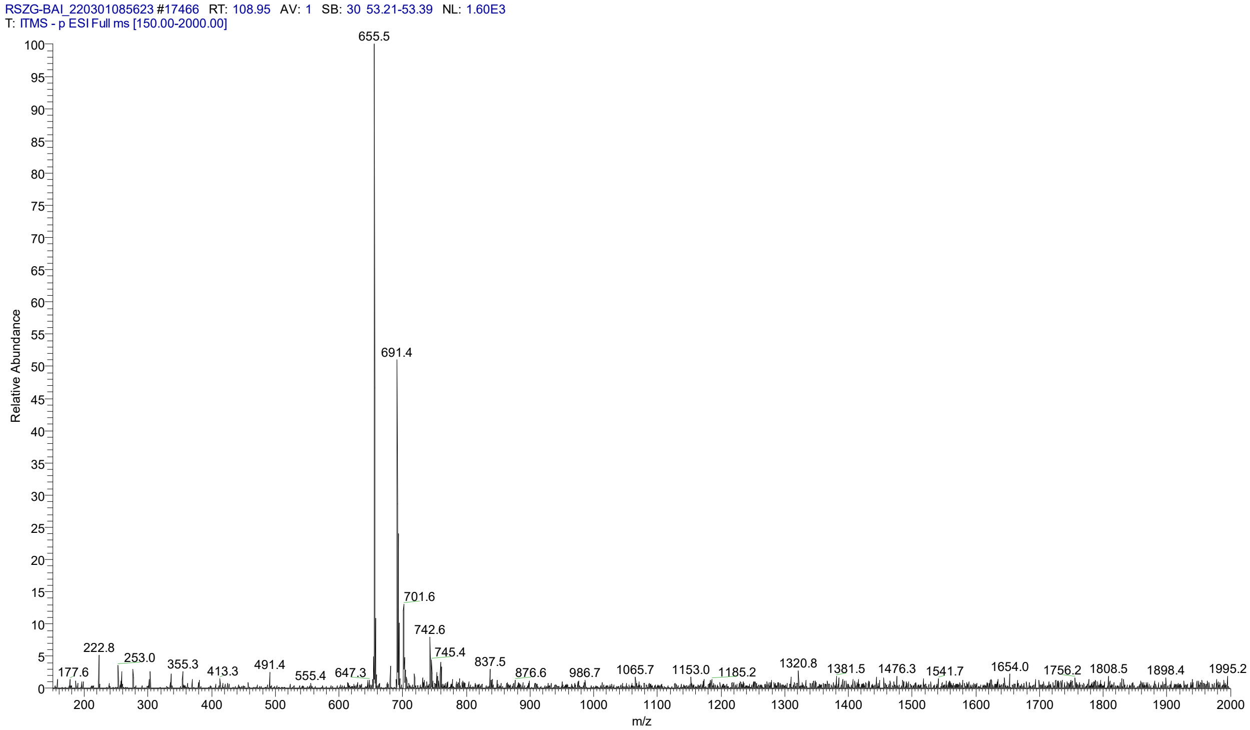
RSZG-BAI_220301085623 #17466 RT: 108.95 AV: 1 SB: 30 53.21-53.39 NL: 1.60E3
T: ITMS - p ESI Full ms [150.00-2000.00]
Relative Abundance
655.5
691.4
701.6
742.6
745.4
222.8
177.6
253.0
355.3
413.3
491.4
555.4
647.3
837.5
876.6
986.7
1065.7
1153.0
1185.2
1320.8
1381.5
1476.3
1541.7
1654.0
1756.2
1808.5
1898.4
1995.2
m/z

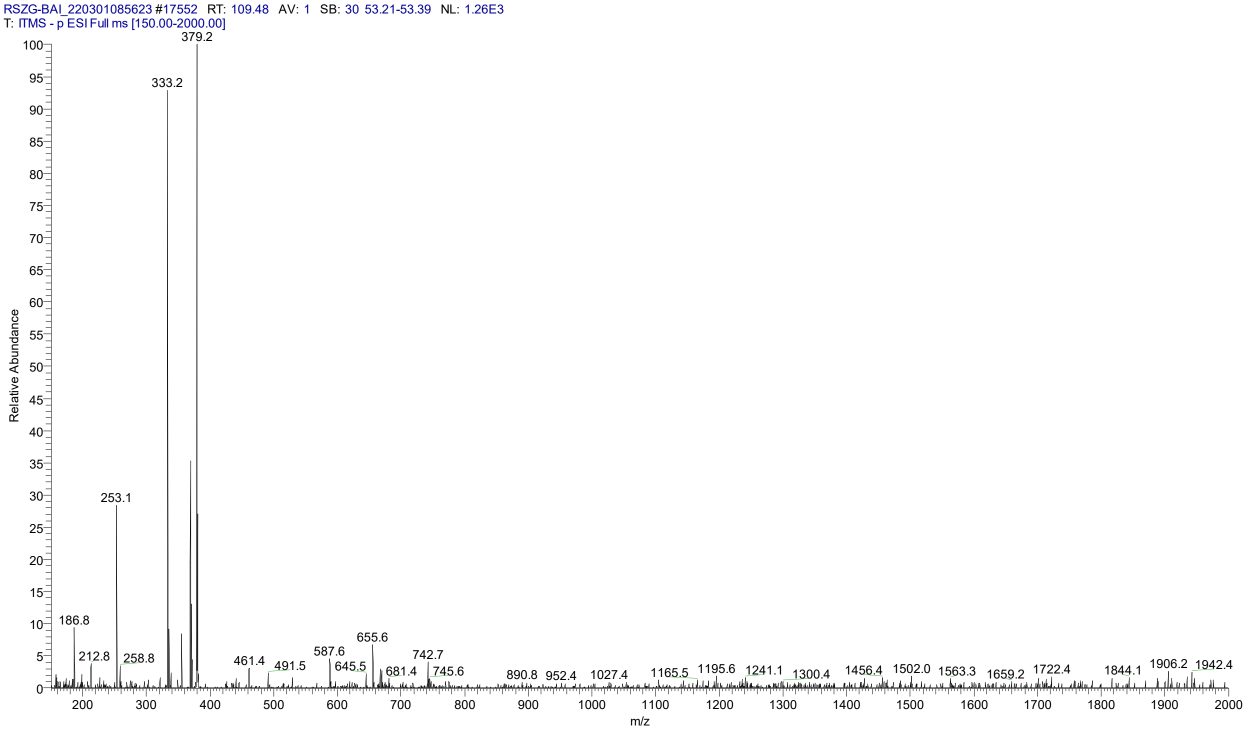
RSZG-BAI_220301085623 #17552 RT: 109.48 AV: 1 SB: 30 53.21-53.39 NL: 1.26E3
T: ITMS - p ESI Full ms [150.00-2000.00]
Relative Abundance
m/z
379.2
333.2
253.1
186.8
212.8
258.8
461.4
491.5
587.6
645.5
655.6
681.4
742.7
745.6
890.8
952.4
1027.4
1165.5
1195.6
1241.1
1300.4
1456.4
1502.0
1563.3
1659.2
1722.4
1844.1
1906.2
1942.4

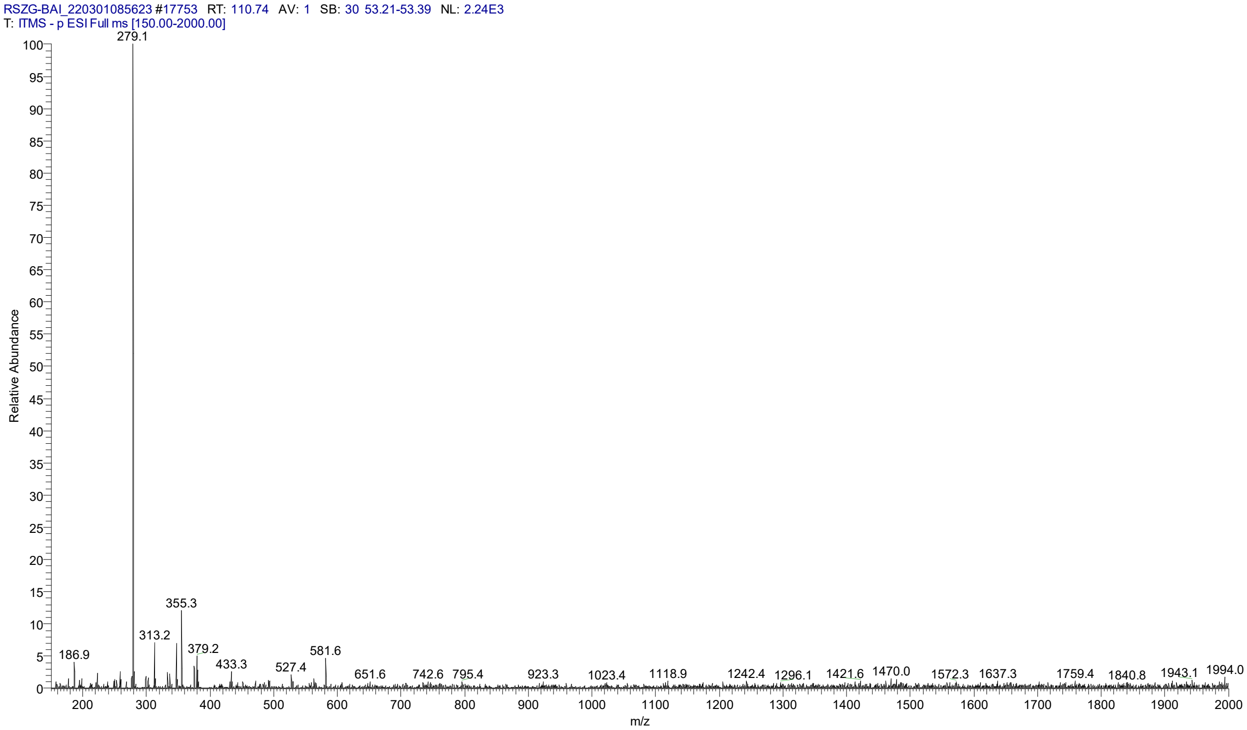
RSZG-BAI_220301085623 #17753 RT: 110.74 AV: 1 SB: 30 53.21-53.39 NL: 2.24E3
T: ITMS - p ESI Full ms [150.00-2000.00]
Relative Abundance
m/z
279.1
355.3
313.2
186.9
379.2
433.3
527.4
581.6
651.6
742.6
795.4
923.3
1023.4
1118.9
1242.4
1296.1
1421.6
1470.0
1572.3
1637.3
1759.4
1840.8
1943.1
1994.0

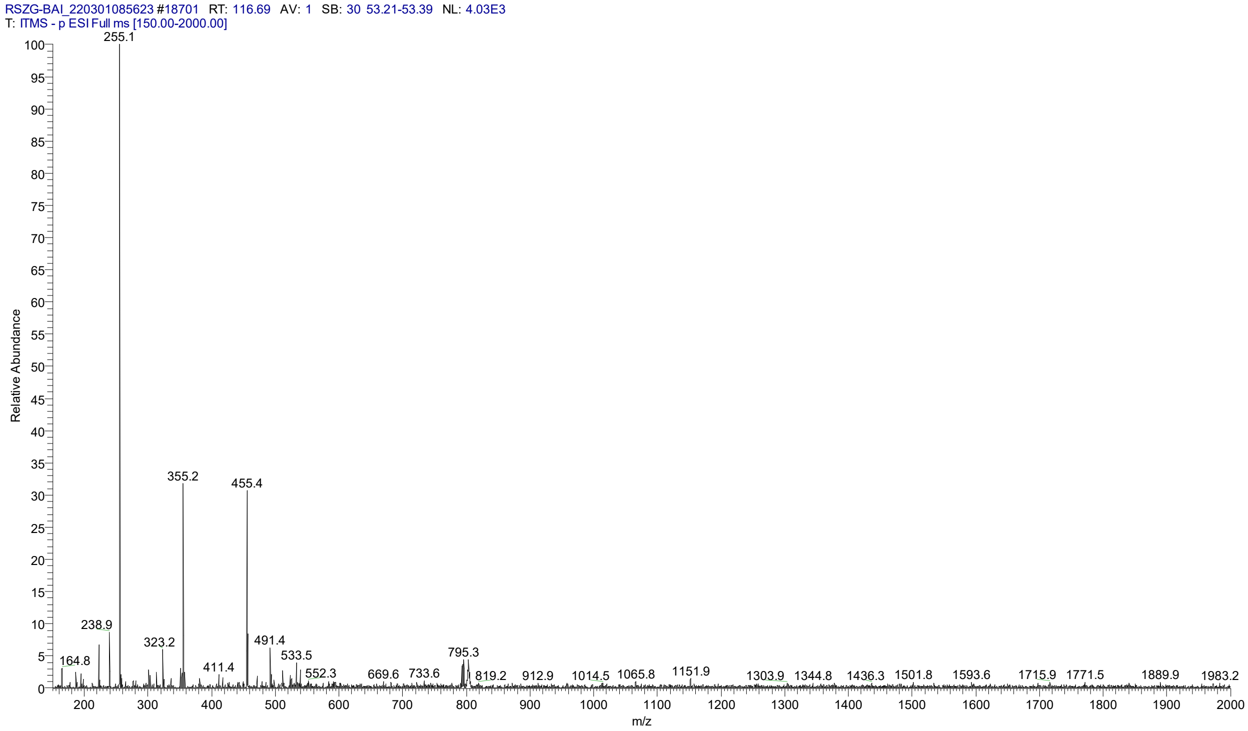

RSZG-BAI_220301085623 #18701 RT: 116.69 AV: 1 SB: 30 53.21-53.39 NL: 4.03E3
T: ITMS - p ESI Full ms [150.00-2000.00]
255.1
355.2
455.4
238.9
323.2
491.4
164.8
533.5
411.4
552.3
669.6
733.6
795.3
819.2
912.9
1014.5
1065.8
1151.9
1303.9
1344.8
1436.3
1501.8
1593.6
1715.9
1771.5
1889.9
1983.2
Relative Abundance
m/z

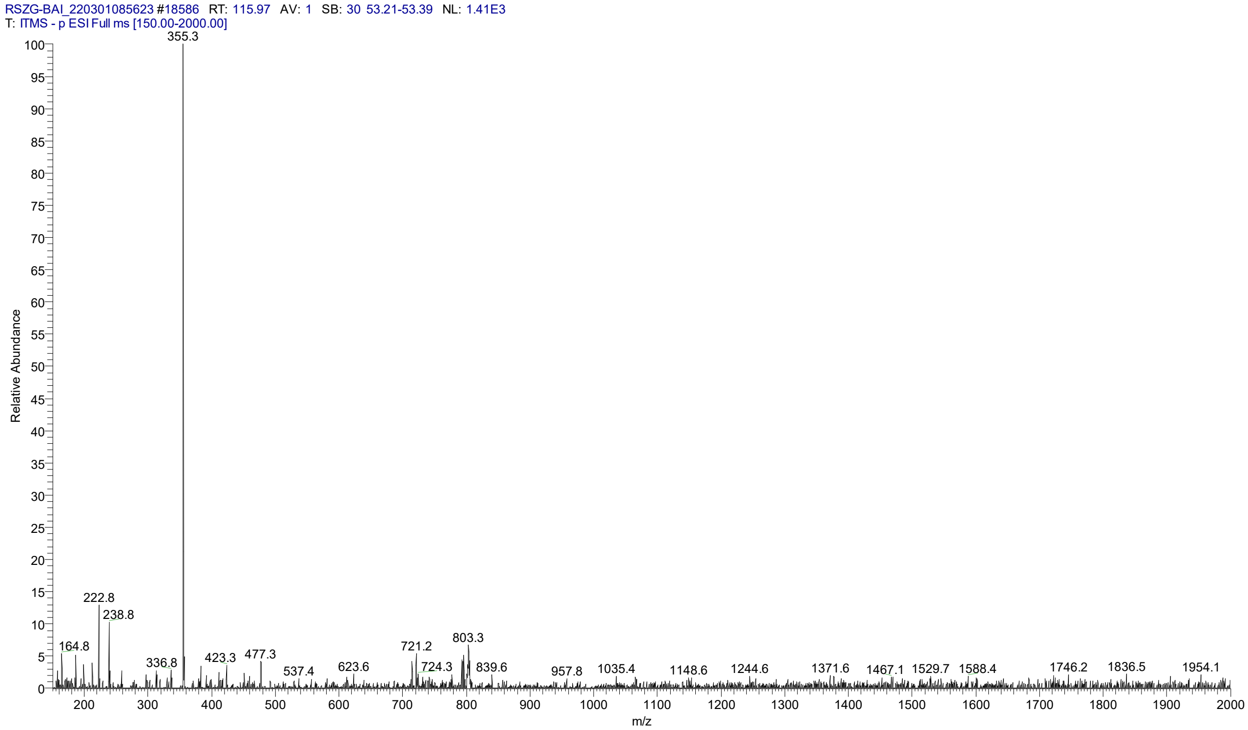

RSZG-BAI_220301085623 #18586 RT: 115.97 AV: 1 SB: 30 53.21-53.39 NL: 1.41E3
T: ITMS - p ESI Full ms [150.00-2000.00]
355.3
222.8
238.8
164.8
336.8
423.3
477.3
537.4
623.6
721.2
724.3
803.3
839.6
957.8
1035.4
1148.6
1244.6
1371.6
1467.1
1529.7
1588.4
1746.2
1836.5
1954.1
Relative Abundance
m/z

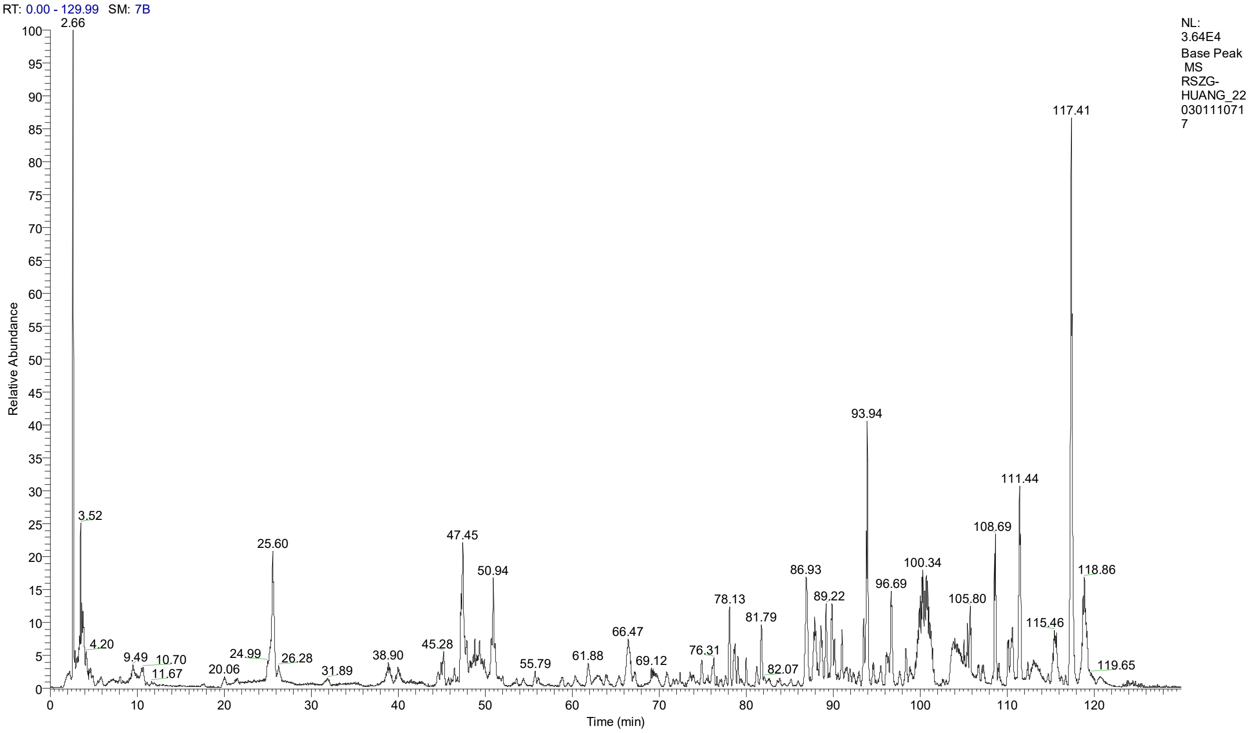

RT: 0.00 - 129.99 SM: 7B
NL: 3.64E4
Base Peak MS
RSZG-HUANG_220301110717
Relative Abundance
Time (min)
2.66
3.52
4.20
9.49
10.70
11.67
20.06
24.99
25.60
26.28
31.89
38.90
45.28
47.45
50.94
55.79
61.88
66.47
69.12
76.31
78.13
81.79
82.07
86.93
89.22
93.94
96.69
100.34
105.80
108.69
111.44
115.46
117.41
118.86
119.65

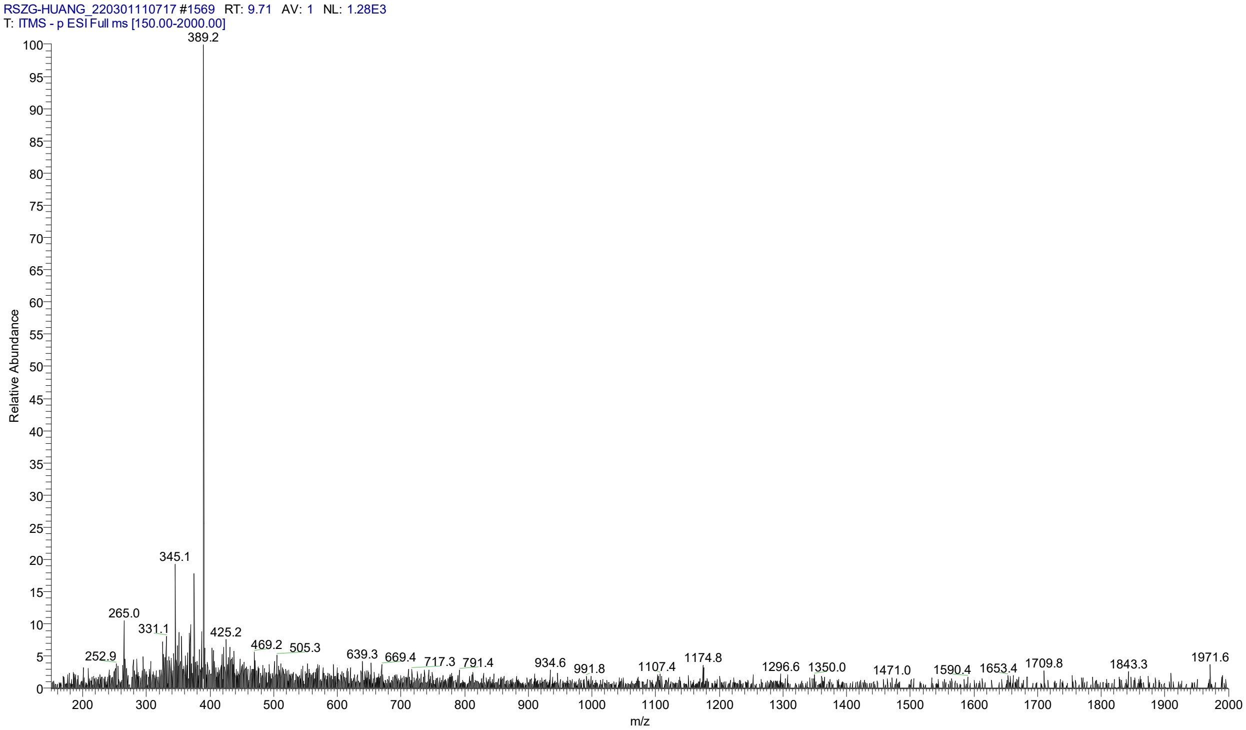

RSZG-HUANG_220301110717 #1569 RT: 9.71 AV: 1 NL: 1.28E3
T: ITMS - p ESI Full ms [150.00-2000.00]
Relative Abundance
m/z
389.2
345.1
265.0
331.1
425.2
252.9
469.2
505.3
639.3
669.4
717.3
791.4
934.6
991.8
1107.4
1174.8
1296.6
1350.0
1471.0
1590.4
1653.4
1709.8
1843.3
1971.6

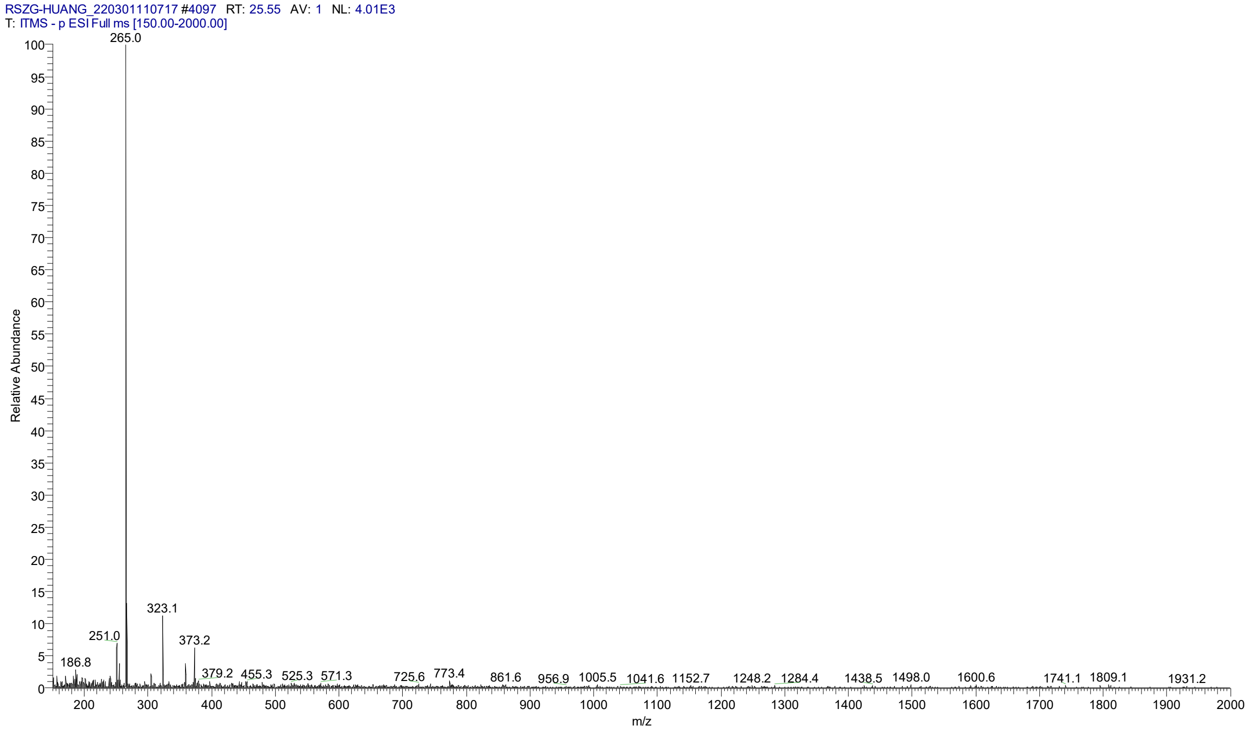
RSZG-HUANG_220301110717 #4097 RT: 25.55 AV: 1 NL: 4.01E3
T: ITMS - p ESI Full ms [150.00-2000.00]
Relative Abundance
m/z

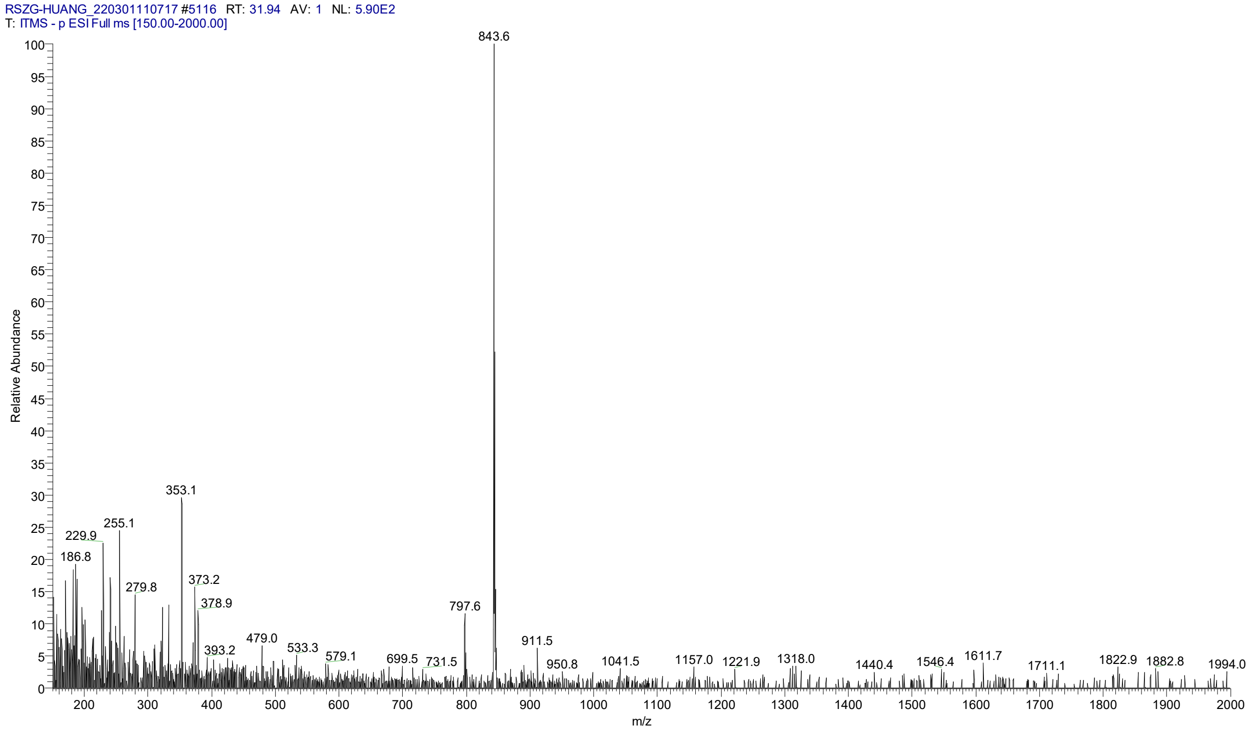
RSZG-HUANG_220301110717 #5116 RT: 31.94 AV: 1 NL: 5.90E2
T: ITMS - p ESI Full ms [150.00-2000.00]
Relative Abundance
m/z

RSZG-HUANG_220301110717 #6207 RT: 38.77 AV: 1 NL: 8.31E2
T: ITMS - p ESI Full ms [150.00-2000.00]

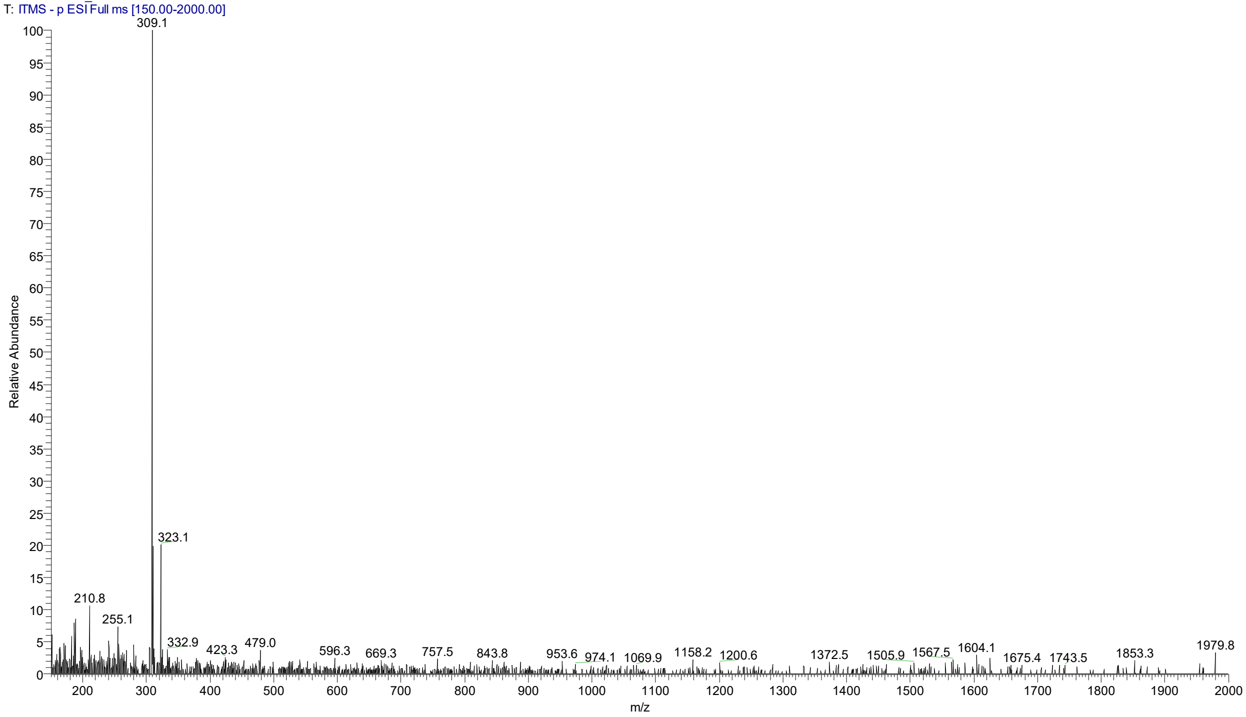

RSZG-HUANG_220301110717 #6394 RT: 39.95 AV: 1 NL: 1.17E3
T: ITMS - p ESI Full ms [150.00-2000.00]

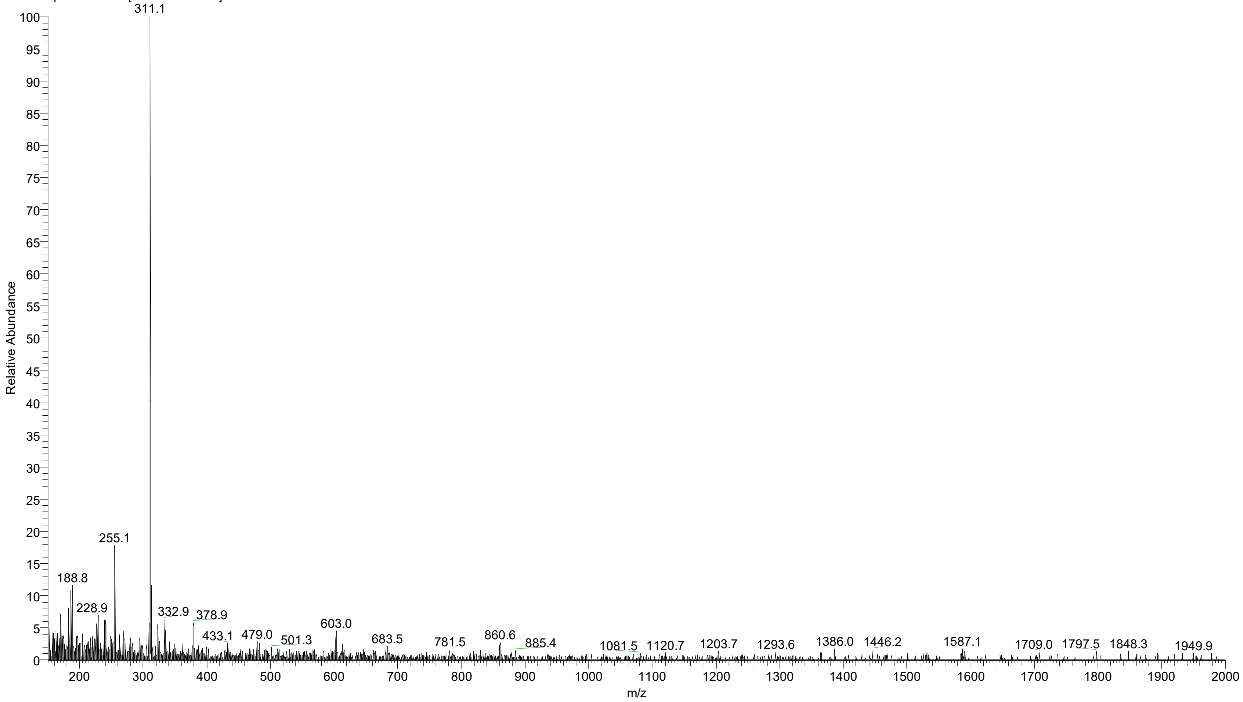

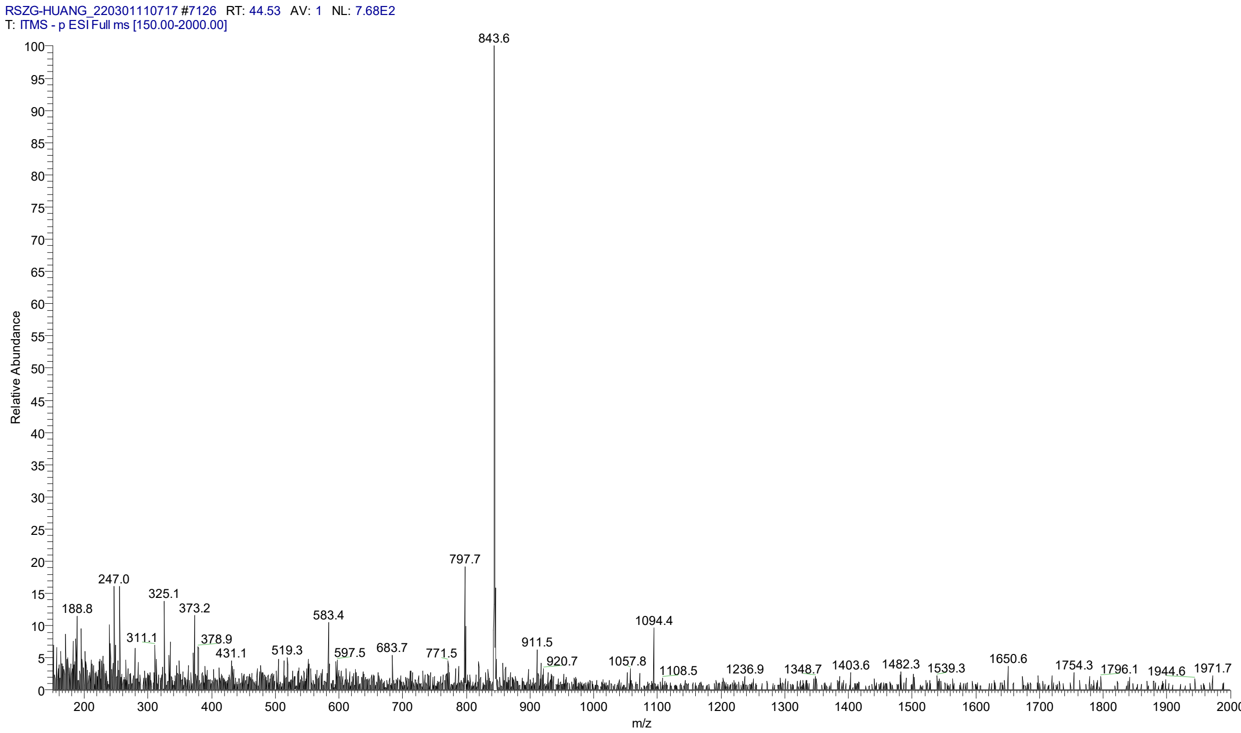
RSZG-HUANG_220301110717 #7126 RT: 44.53 AV: 1 NL: 7.68E2
T: ITMS - p ESI Full ms [150.00-2000.00]
Relative Abundance
m/z
843.6
797.7
247.0
325.1
373.2
188.8
311.1
378.9
431.1
519.3
583.4
597.5
683.7
771.5
911.5
920.7
1057.8
1094.4
1108.5
1236.9
1348.7
1403.6
1482.3
1539.3
1650.6
1754.3
1796.1
1944.6
1971.7

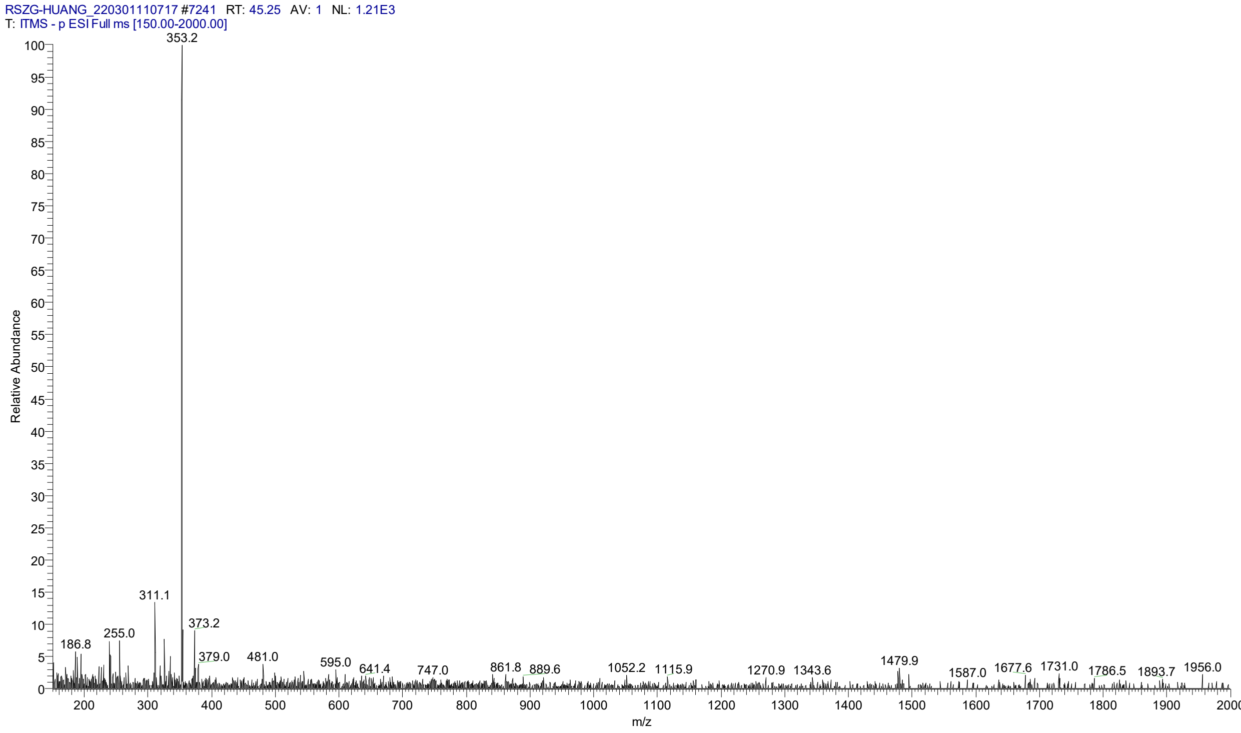
RSZG-HUANG_220301110717 #7241 RT: 45.25 AV: 1 NL: 1.21E3
T: ITMS - p ESI Full ms [150.00-2000.00]
Relative Abundance
m/z
353.2
311.1
373.2
255.0
186.8
379.0
481.0
595.0
641.4
747.0
861.8
889.6
1052.2
1115.9
1270.9
1343.6
1479.9
1587.0
1677.6
1731.0
1786.5
1893.7
1956.0

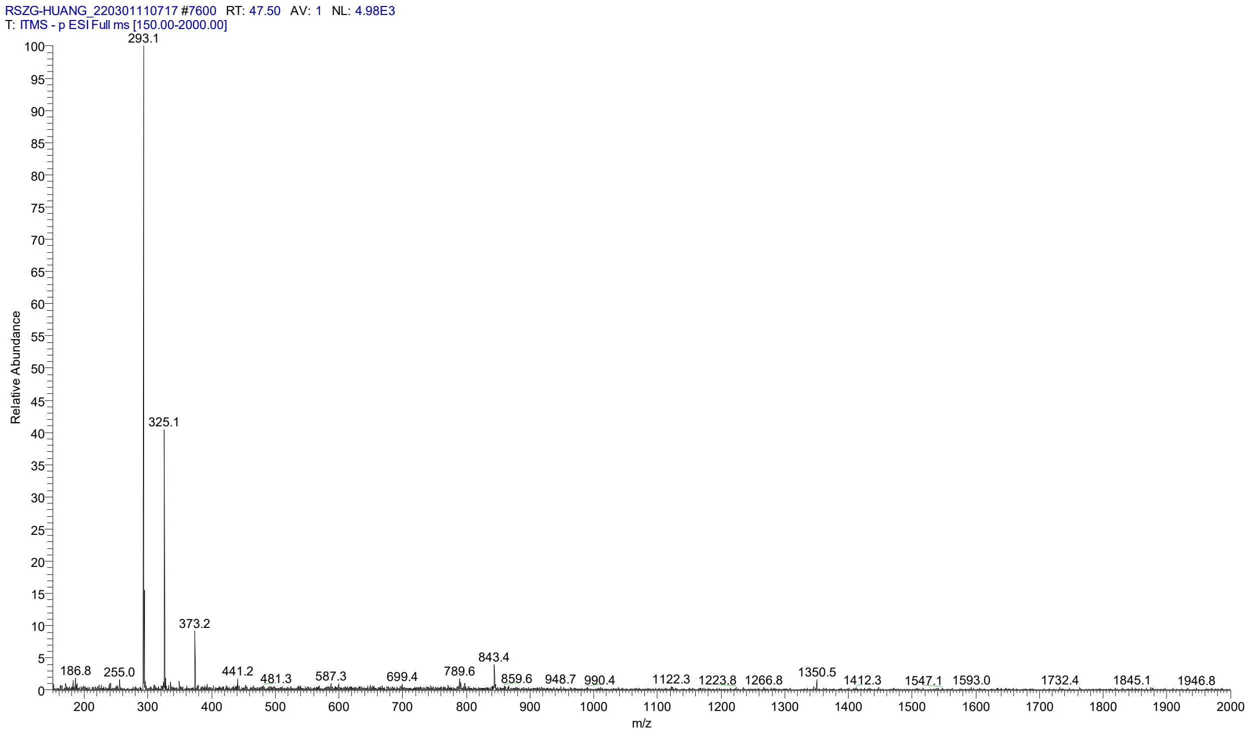
RSZG-HUANG_220301110717 #7600 RT: 47.50 AV: 1 NL: 4.98E3
T: ITMS - p ESI Full ms [150.00-2000.00]
293.1
325.1
373.2
186.8
255.0
441.2
481.3
587.3
699.4
789.6
843.4
859.6
948.7
990.4
1122.3
1223.8
1266.8
1350.5
1412.3
1547.1
1593.0
1732.4
1845.1
1946.8
Relative Abundance
m/z

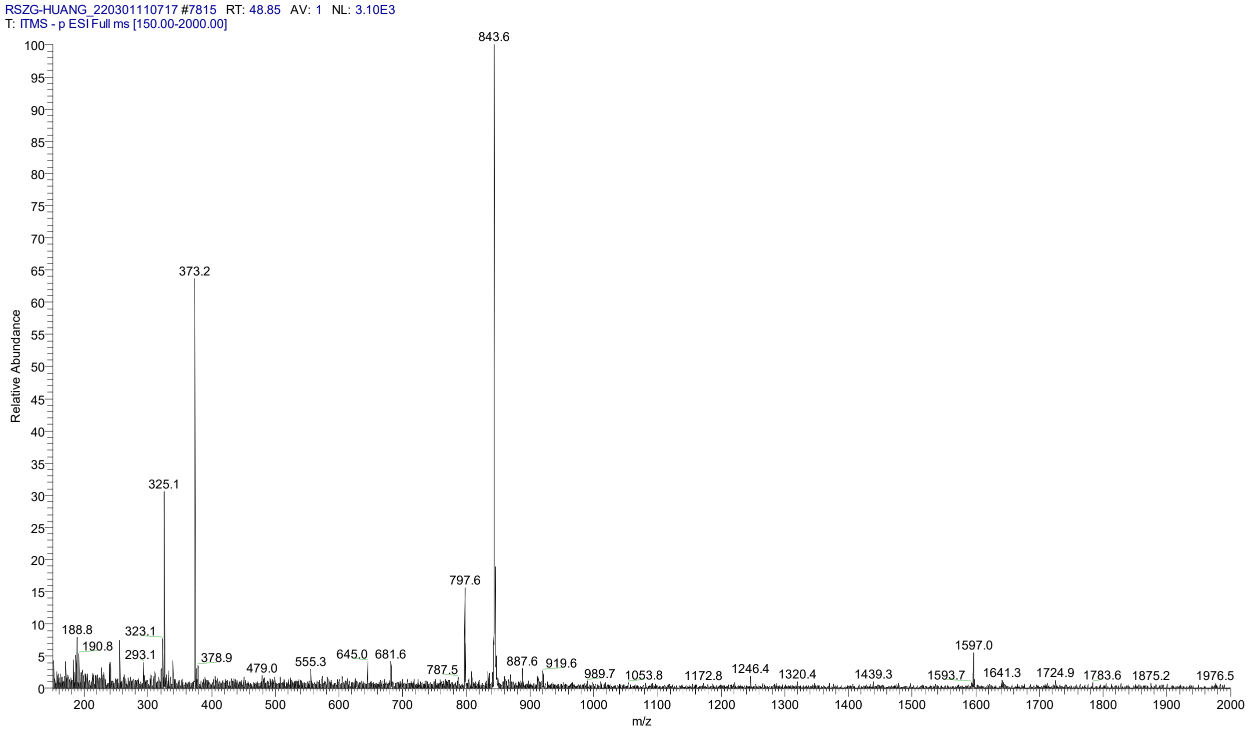
RSZG-HUANG_220301110717 #7815 RT: 48.85 AV: 1 NL: 3.10E3
T: ITMS - p ESI Full ms [150.00-2000.00]
843.6
373.2
325.1
797.6
188.8
190.8
323.1
293.1
378.9
479.0
555.3
645.0
681.6
787.5
887.6
919.6
989.7
1053.8
1172.8
1246.4
1320.4
1439.3
1593.7
1597.0
1641.3
1724.9
1783.6
1875.2
1976.5
Relative Abundance
m/z

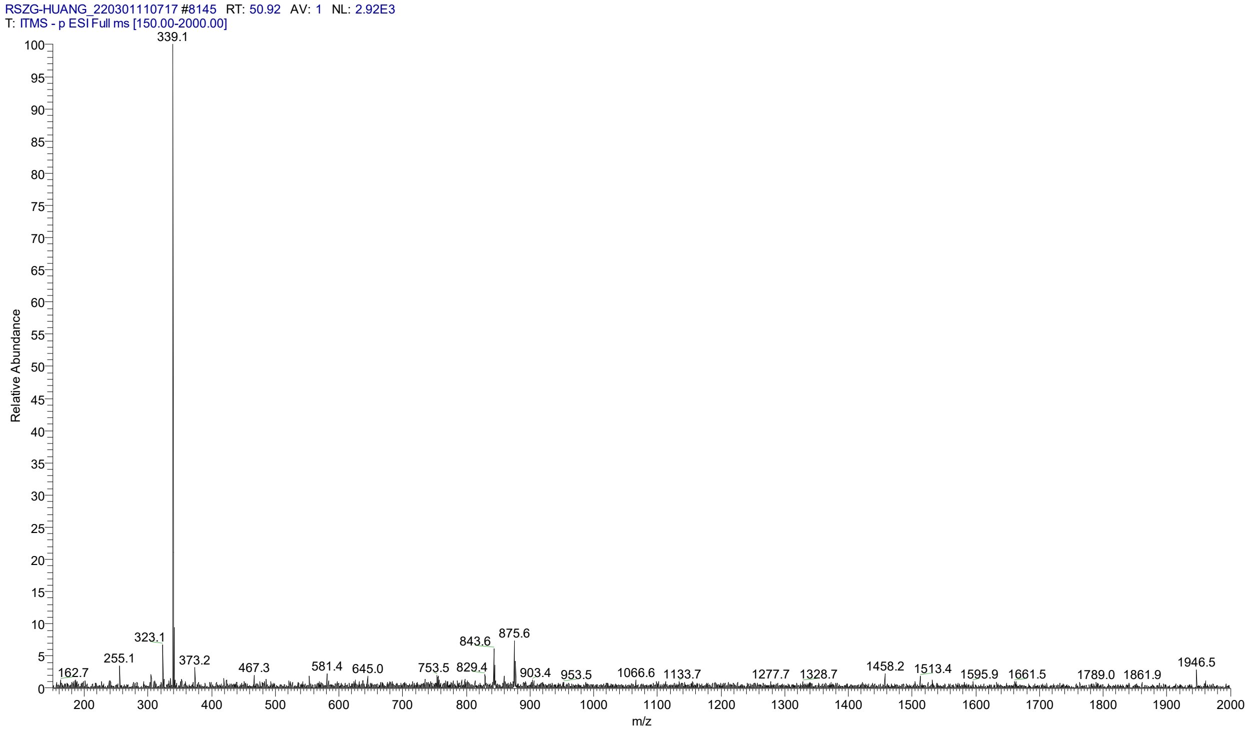
RSZG-HUANG_220301110717 #8145 RT: 50.92 AV: 1 NL: 2.92E3
T: ITMS - p ESI Full ms [150.00-2000.00]
Relative Abundance
m/z
339.1
323.1
255.1
373.2
162.7
467.3
581.4
645.0
753.5
829.4
843.6
875.6
903.4
953.5
1066.6
1133.7
1277.7
1328.7
1458.2
1513.4
1595.9
1661.5
1789.0
1861.9
1946.5

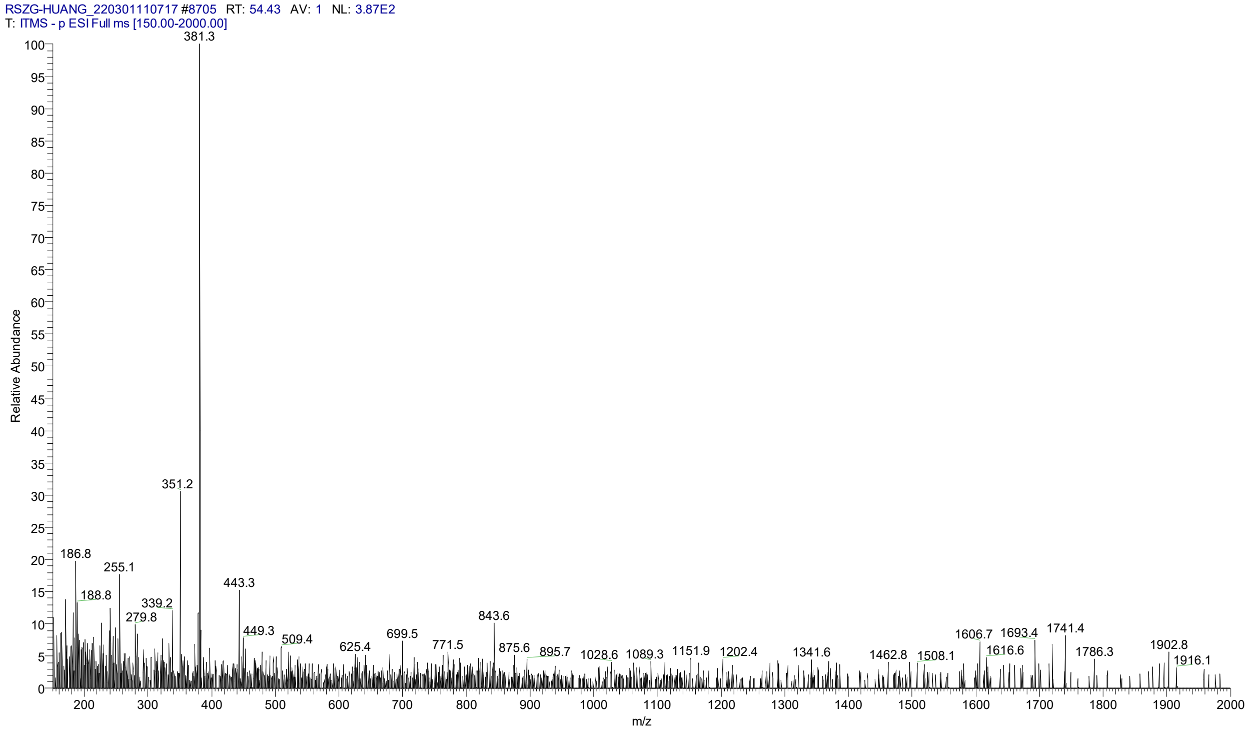
RSZG-HUANG_220301110717 #8705 RT: 54.43 AV: 1 NL: 3.87E2
T: ITMS - p ESI Full ms [150.00-2000.00]
Relative Abundance
m/z
381.3
351.2
186.8
255.1
188.8
339.2
279.8
443.3
449.3
509.4
625.4
699.5
771.5
843.6
875.6
895.7
1028.6
1089.3
1151.9
1202.4
1341.6
1462.8
1508.1
1606.7
1616.6
1693.4
1741.4
1786.3
1902.8
1916.1

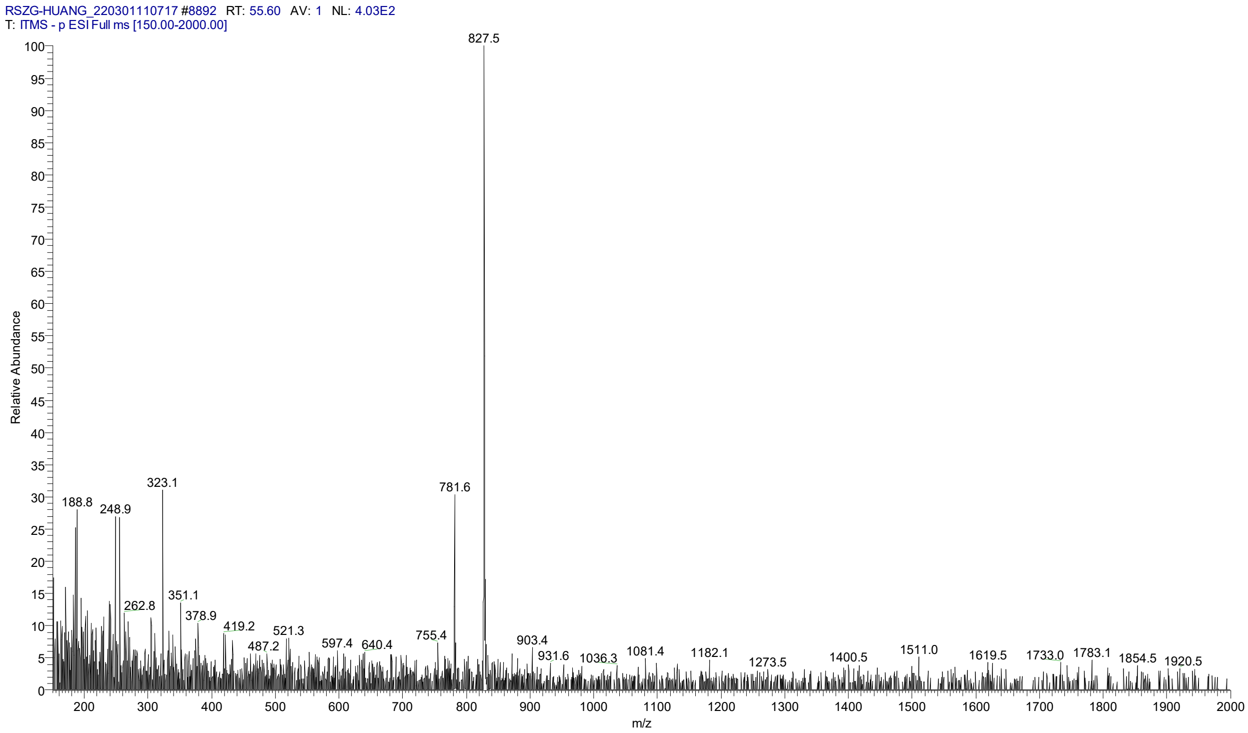

RSZG-HUANG_220301110717 #8892 RT: 55.60 AV: 1 NL: 4.03E2
T: ITMS - p ESI Full ms [150.00-2000.00]
Relative Abundance
m/z
827.5
781.6
323.1
188.8
248.9
262.8
351.1
378.9
419.2
487.2
521.3
597.4
640.4
755.4
903.4
931.6
1036.3
1081.4
1182.1
1273.5
1400.5
1511.0
1619.5
1733.0
1783.1
1854.5
1920.5

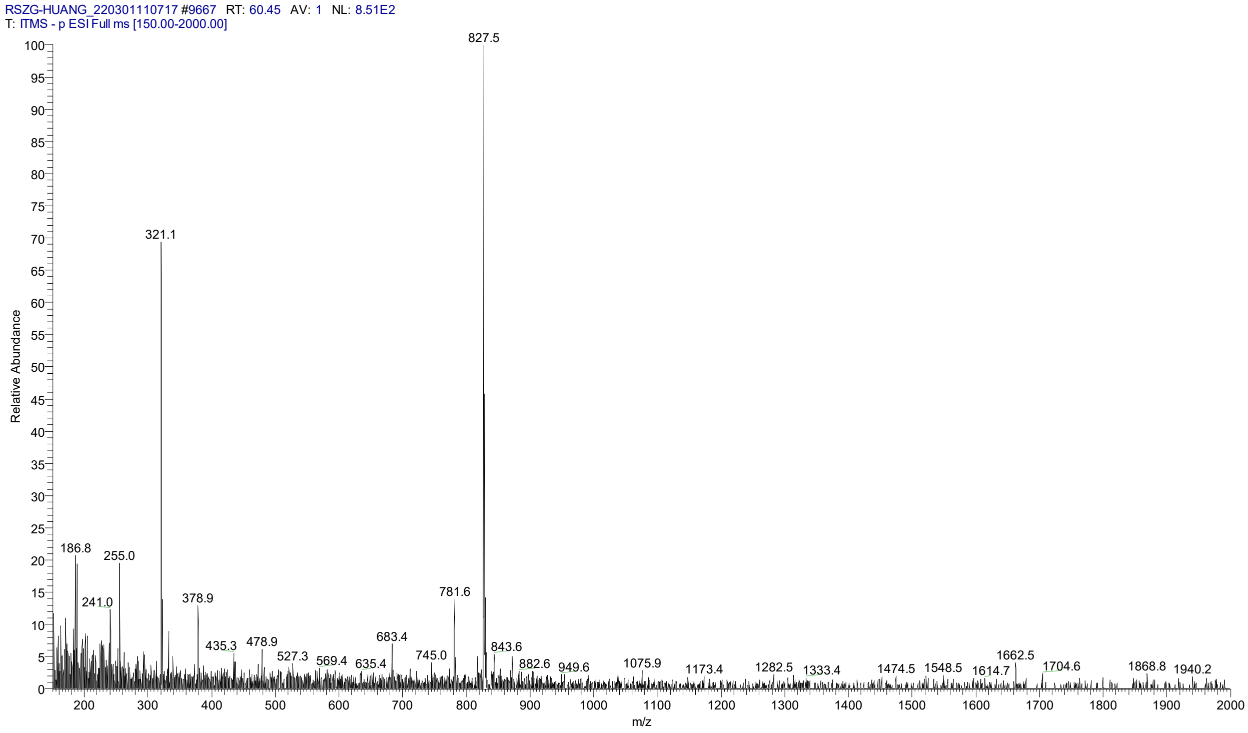

RSZG-HUANG_220301110717 #9667 RT: 60.45 AV: 1 NL: 8.51E2
T: ITMS - p ESI Full ms [150.00-2000.00]
Relative Abundance
m/z
827.5
321.1
186.8
255.0
241.0
378.9
781.6
435.3
478.9
527.3
569.4
635.4
683.4
745.0
843.6
882.6
949.6
1075.9
1173.4
1282.5
1333.4
1474.5
1548.5
1614.7
1662.5
1704.6
1868.8
1940.2

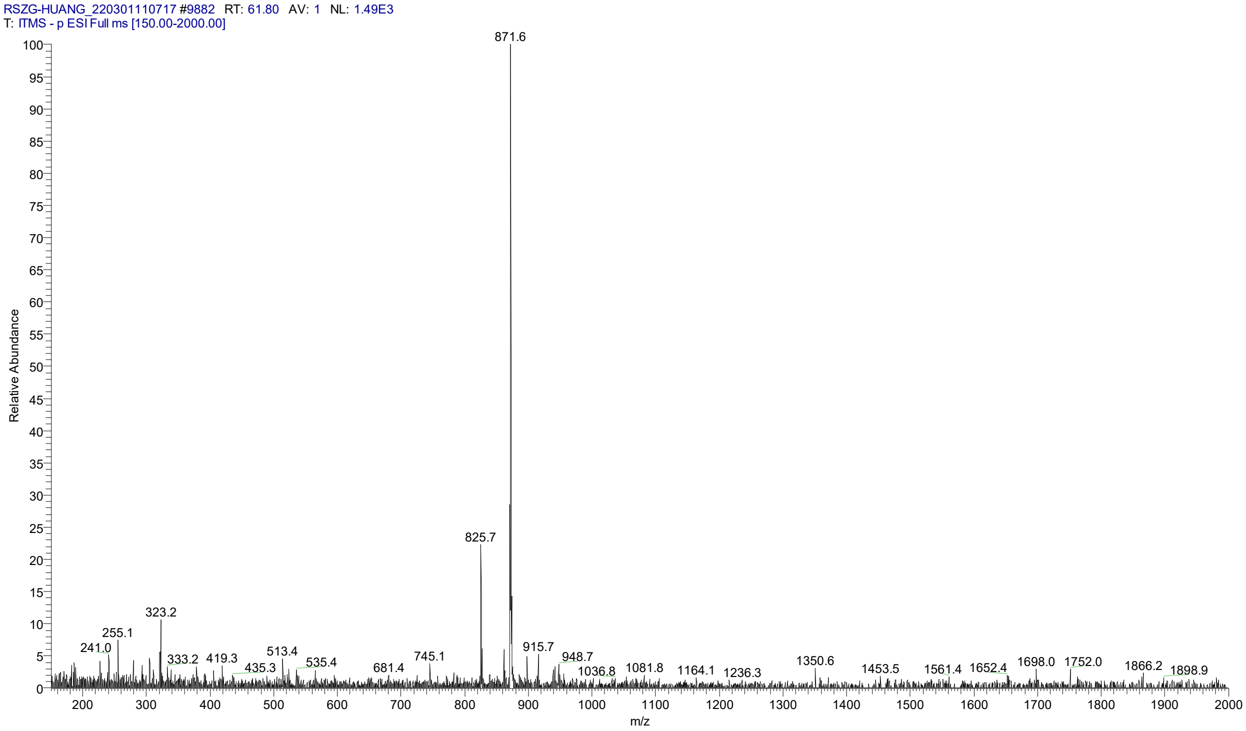
RSZG-HUANG_220301110717 #9882 RT: 61.80 AV: 1 NL: 1.49E3
T: ITMS - p ESI Full ms [150.00-2000.00]
Relative Abundance
m/z
871.6
825.7
323.2
255.1
241.0
333.2
419.3
435.3
513.4
535.4
681.4
745.1
915.7
948.7
1036.8
1081.8
1164.1
1236.3
1350.6
1453.5
1561.4
1652.4
1698.0
1752.0
1866.2
1898.9

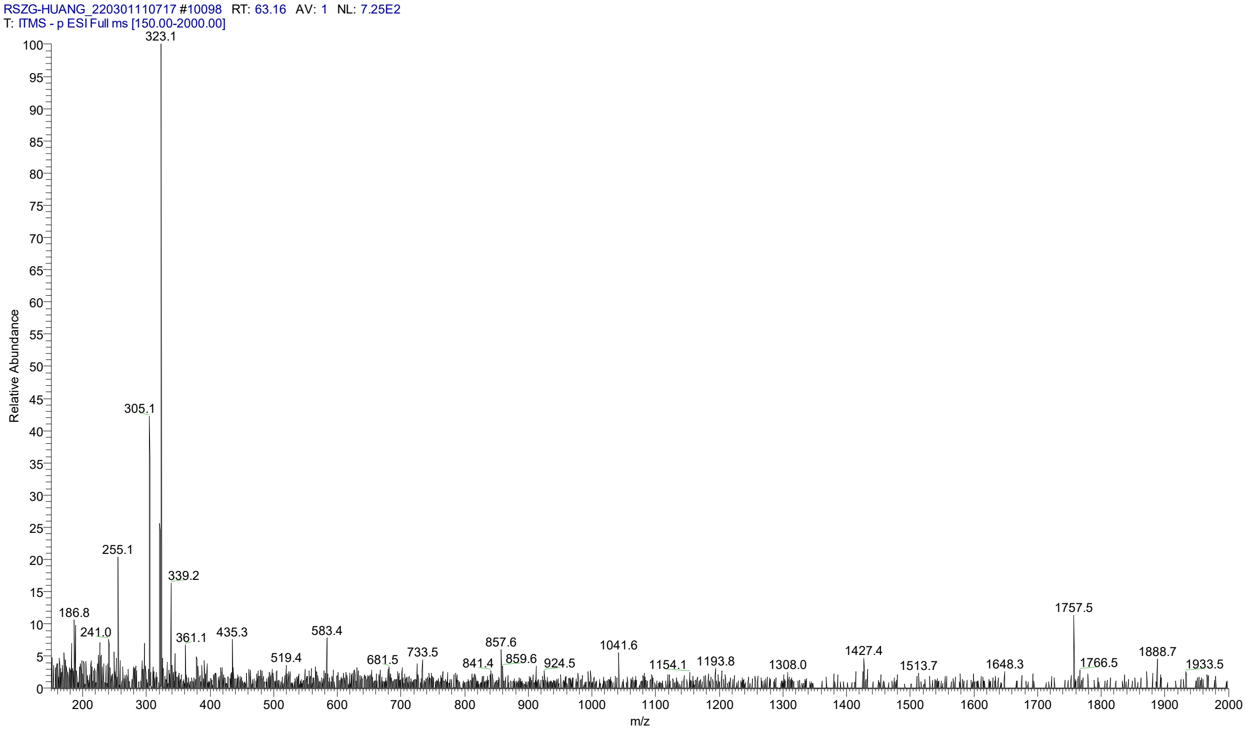
RSZG-HUANG_220301110717 #10098 RT: 63.16 AV: 1 NL: 7.25E2
T: ITMS - p ESI Full ms [150.00-2000.00]
Relative Abundance
m/z
323.1
305.1
255.1
339.2
186.8
241.0
361.1
435.3
519.4
583.4
681.5
733.5
841.4
857.6
859.6
924.5
1041.6
1154.1
1193.8
1308.0
1427.4
1513.7
1648.3
1757.5
1766.5
1888.7
1933.5

RSZG-HUANG_220301110717 #10227 RT: 63.96 AV: 1 NL: 6.33E2
T: ITMS - p ESI Full ms [150.00-2000.00]

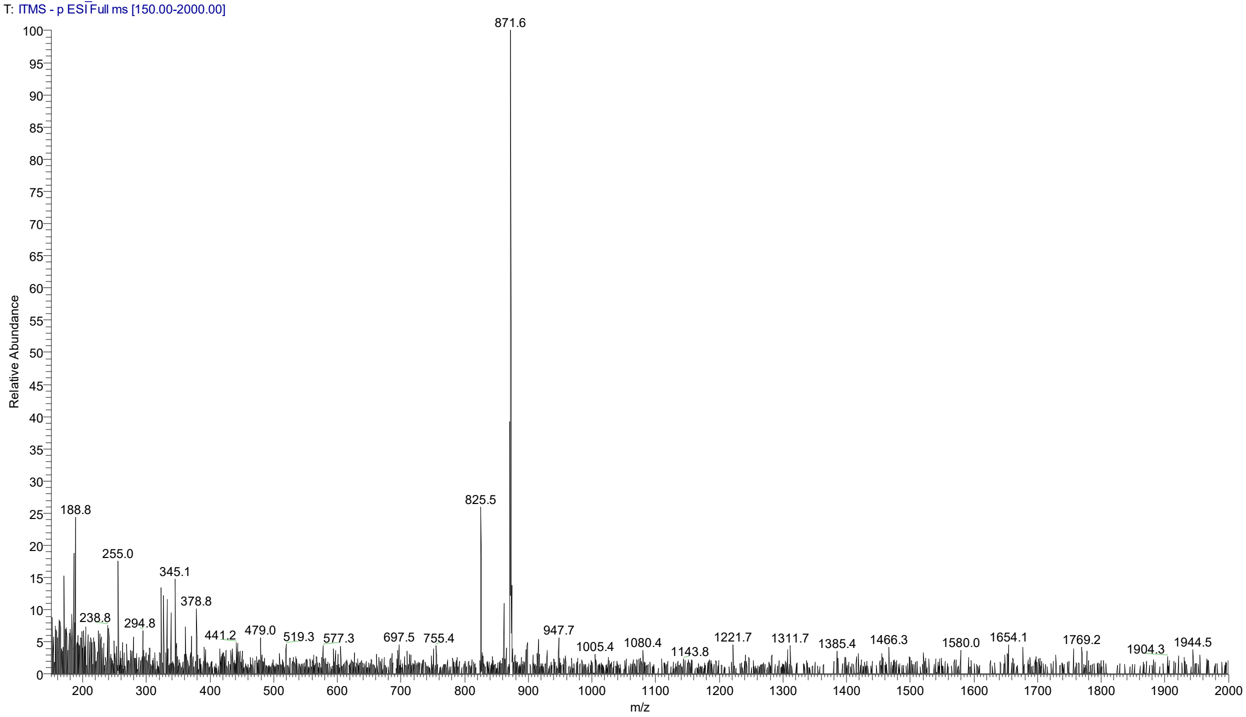

RSZG-HUANG_220301110717 #10442 RT: 65.31 AV: 1 NL: 6.87E2
T: ITMS - p ESI Full ms [150.00-2000.00]

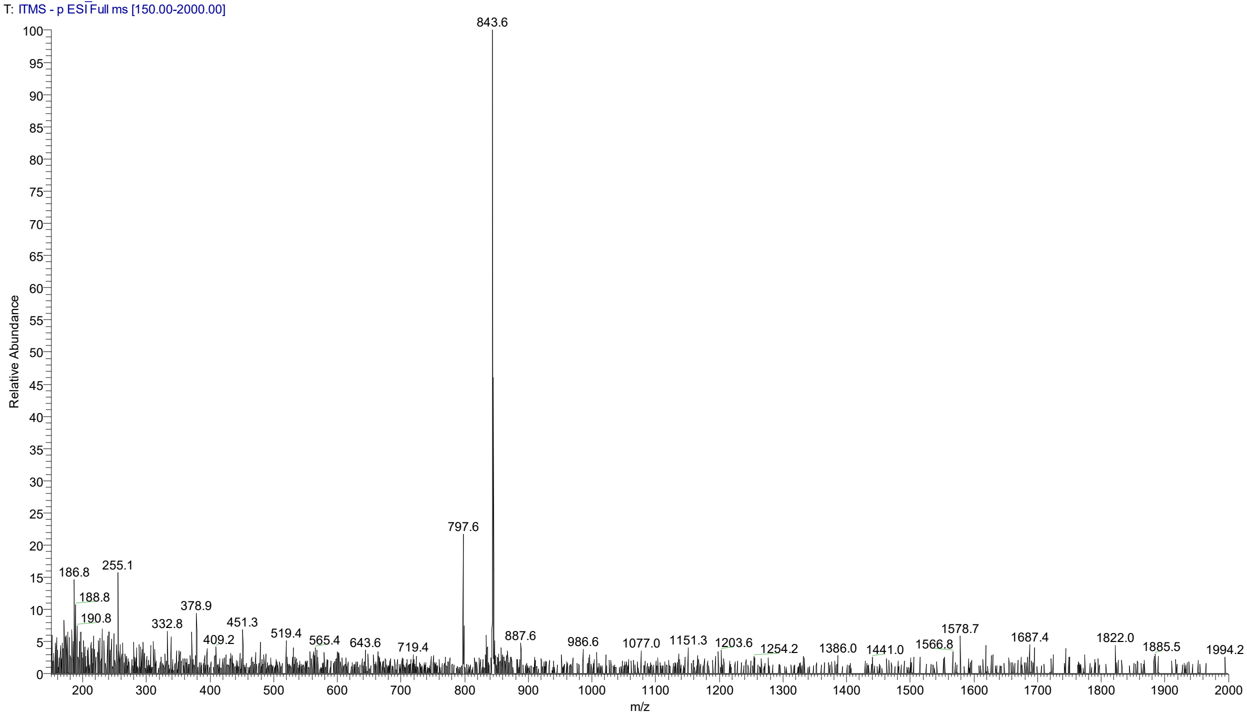

RSZG-HUANG_220301110717 #10643 RT: 66.57 AV: 1 NL: 1.41E3
T: ITMS - p ESI Full ms [150.00-2000.00]

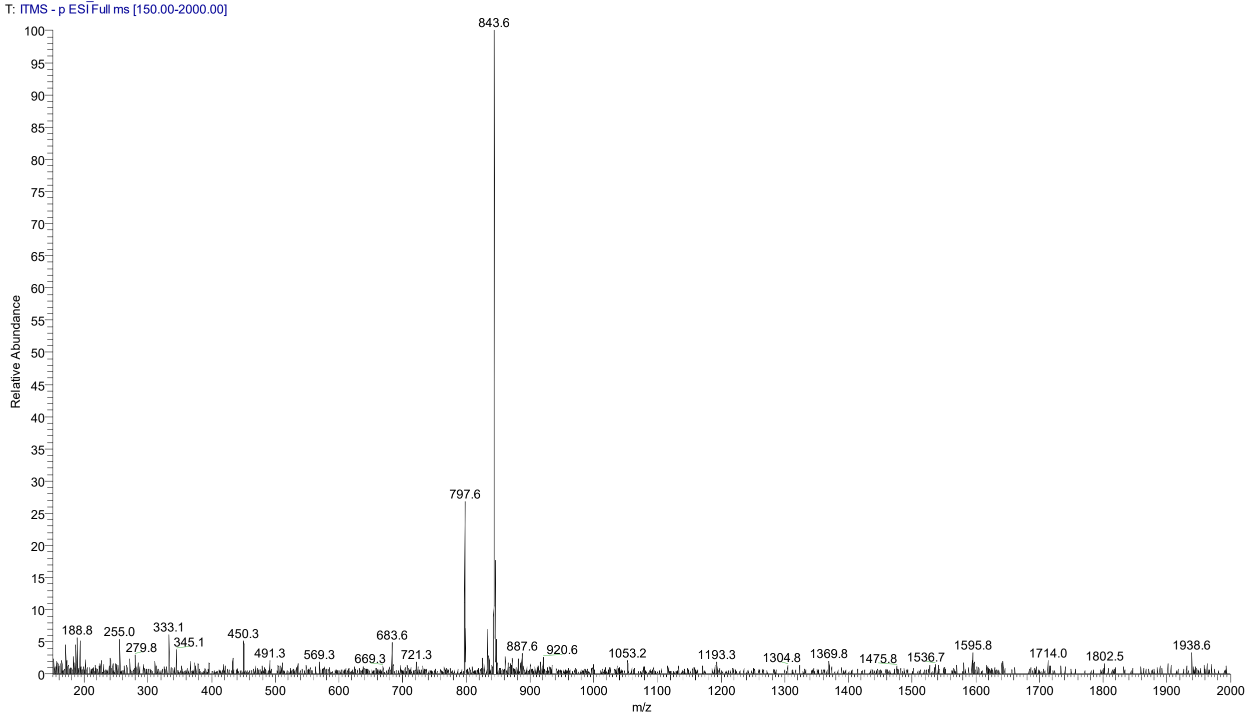

RSZG-HUANG_220301110717 #11118 RT: 69.54 AV: 1 NL: 9.79E2
T: ITMS - p ESI Full ms [150.00-2000.00]

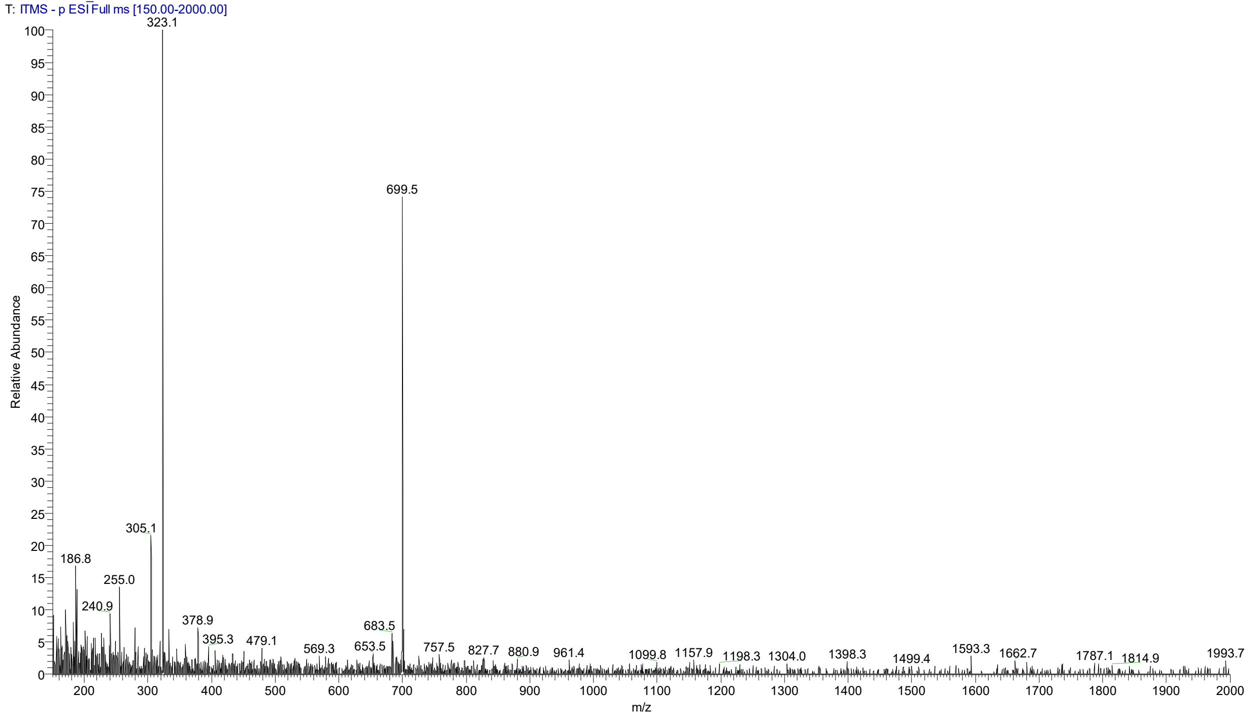

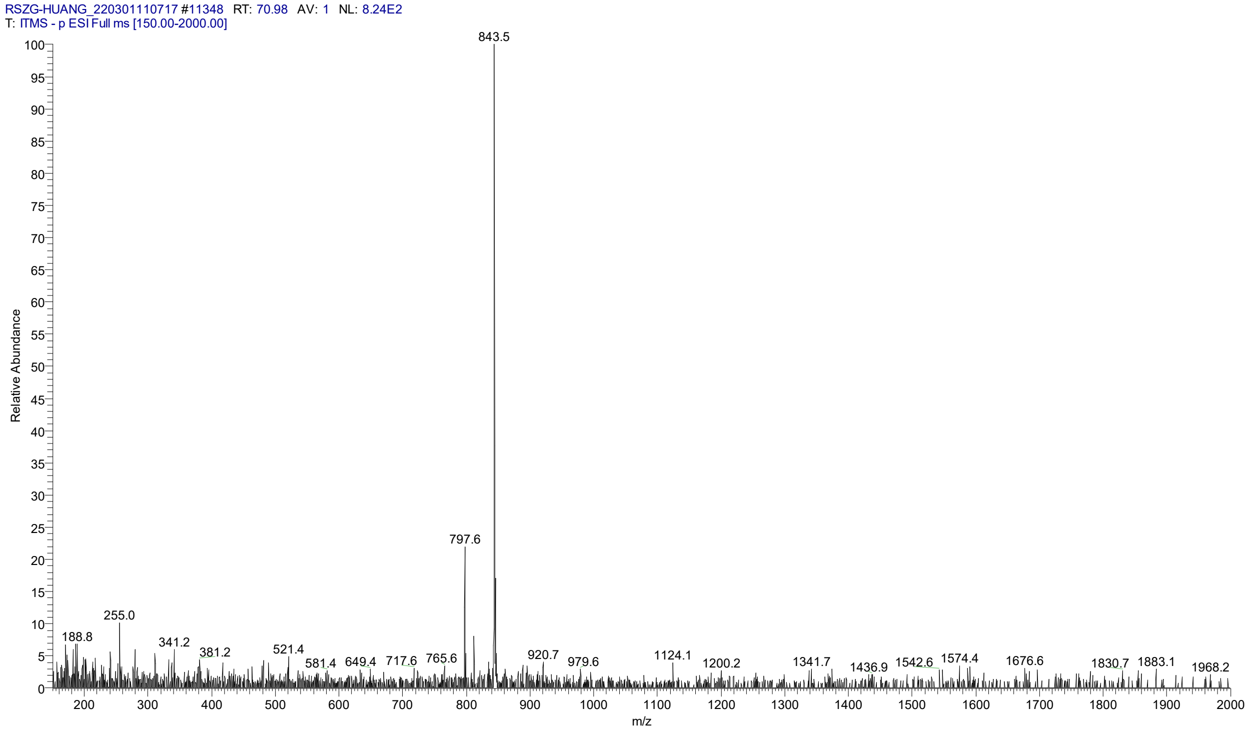
RSZG-HUANG_220301110717 #11348 RT: 70.98 AV: 1 NL: 8.24E2
T: ITMS - p ESI Full ms [150.00-2000.00]
843.5
797.6
255.0
188.8
341.2
381.2
521.4
581.4
649.4
717.6
765.6
920.7
979.6
1124.1
1200.2
1341.7
1436.9
1542.6
1574.4
1676.6
1830.7
1883.1
1968.2
Relative Abundance
m/z

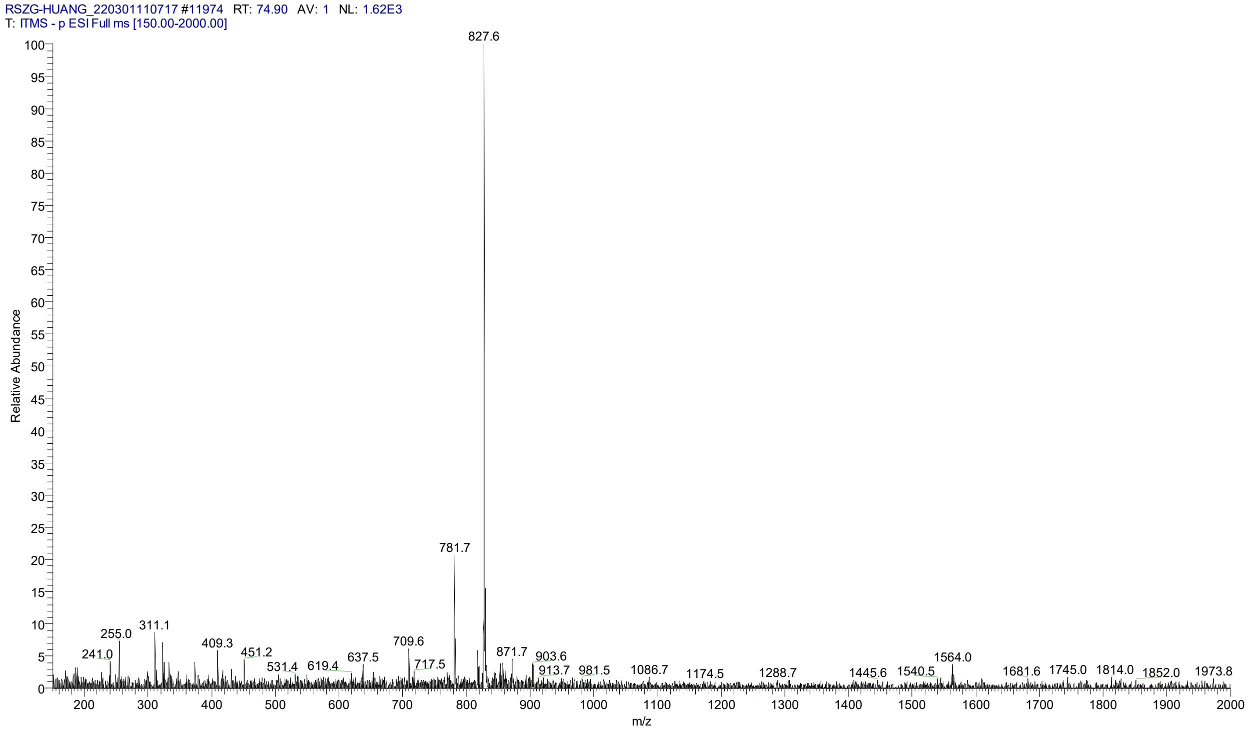
RSZG-HUANG_220301110717 #11974 RT: 74.90 AV: 1 NL: 1.62E3
T: ITMS - p ESI Full ms [150.00-2000.00]
827.6
781.7
255.0
241.0
311.1
409.3
451.2
531.4
619.4
637.5
709.6
717.5
871.7
903.6
913.7
981.5
1086.7
1174.5
1288.7
1445.6
1540.5
1564.0
1681.6
1745.0
1814.0
1852.0
1973.8
Relative Abundance
m/z

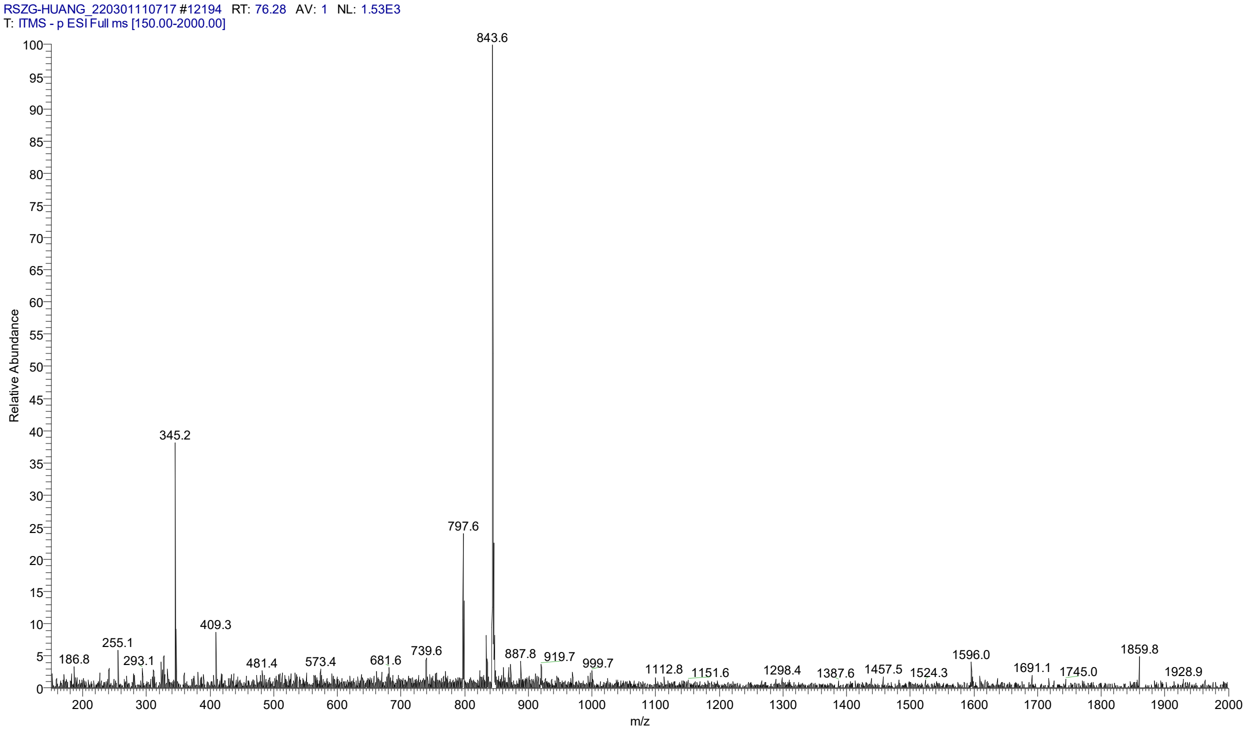

RSZG-HUANG_220301110717 #12194 RT: 76.28 AV: 1 NL: 1.53E3
T: ITMS - p ESI Full ms [150.00-2000.00]
843.6
345.2
797.6
409.3
255.1
186.8
293.1
481.4
573.4
681.6
739.6
887.8
919.7
999.7
1112.8
1151.6
1298.4
1387.6
1457.5
1524.3
1596.0
1691.1
1745.0
1859.8
1928.9
Relative Abundance
m/z

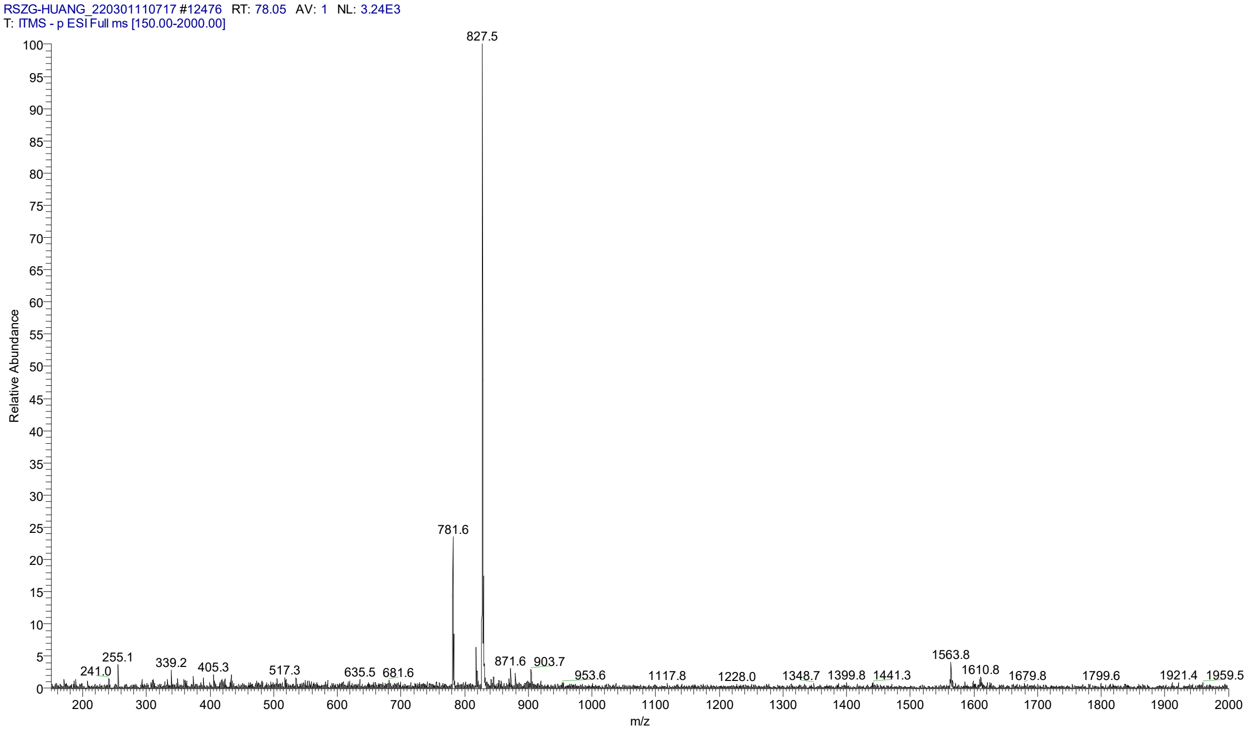

RSZG-HUANG_220301110717 #12476 RT: 78.05 AV: 1 NL: 3.24E3
T: ITMS - p ESI Full ms [150.00-2000.00]
827.5
781.6
241.0
255.1
339.2
405.3
517.3
635.5
681.6
871.6
903.7
953.6
1117.8
1228.0
1348.7
1399.8
1441.3
1563.8
1610.8
1679.8
1799.6
1921.4
1959.5
Relative Abundance
m/z

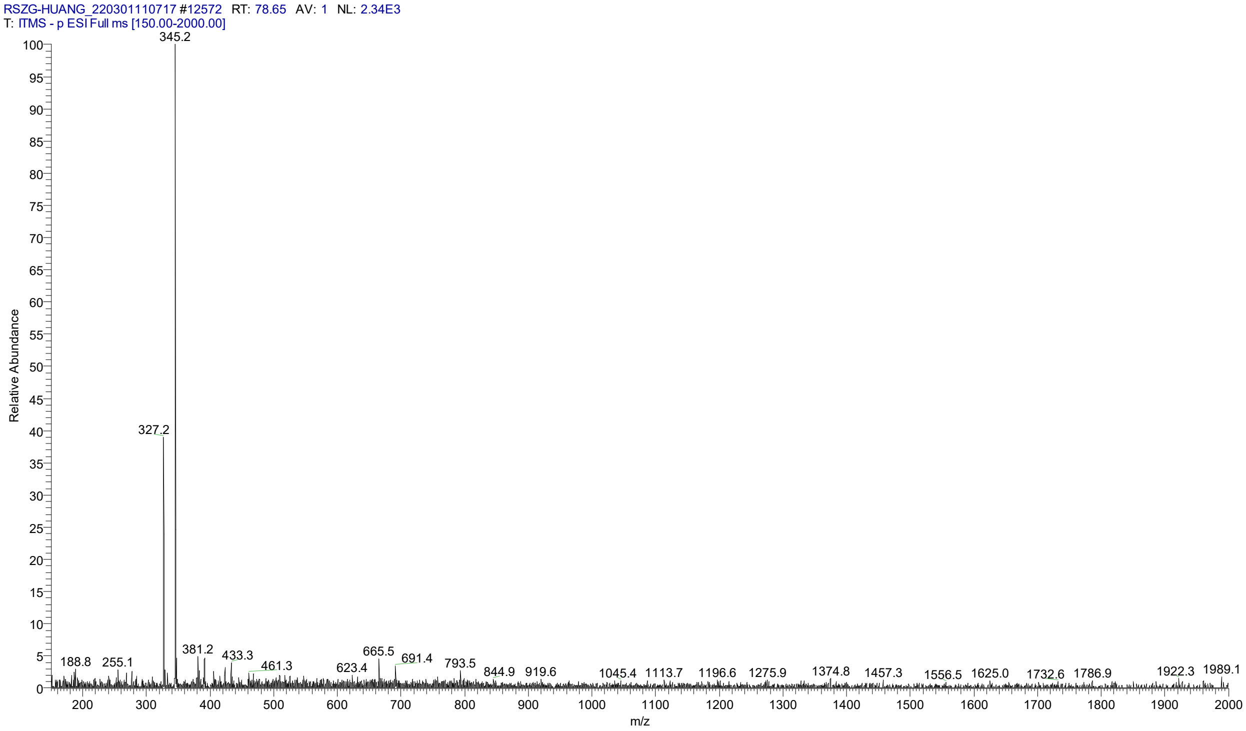
RSZG-HUANG_220301110717 #12572 RT: 78.65 AV: 1 NL: 2.34E3
T: ITMS - p ESI Full ms [150.00-2000.00]
Relative Abundance
m/z
345.2
327.2
188.8
255.1
381.2
433.3
461.3
623.4
665.5
691.4
793.5
844.9
919.6
1045.4
1113.7
1196.6
1275.9
1374.8
1457.3
1556.5
1625.0
1732.6
1786.9
1922.3
1989.1

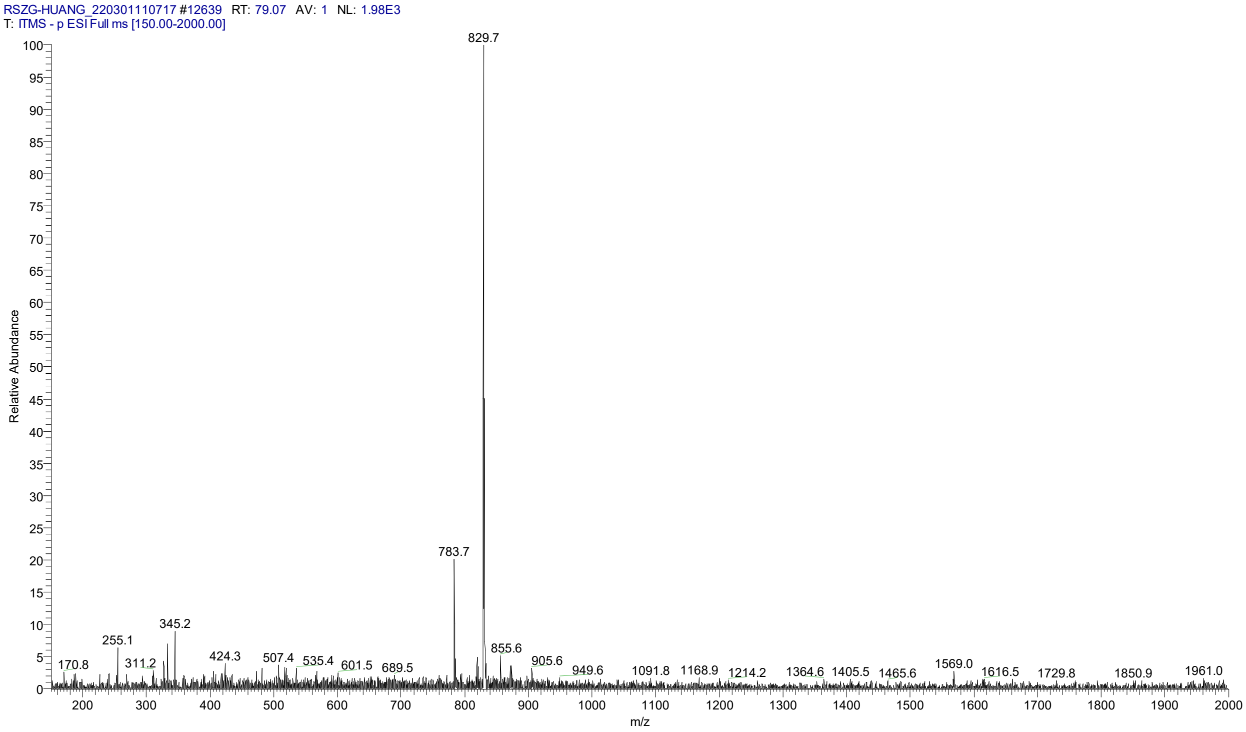
RSZG-HUANG_220301110717 #12639 RT: 79.07 AV: 1 NL: 1.98E3
T: ITMS - p ESI Full ms [150.00-2000.00]
Relative Abundance
m/z
829.7
783.7
345.2
255.1
170.8
311.2
424.3
507.4
535.4
601.5
689.5
855.6
905.6
949.6
1091.8
1168.9
1214.2
1364.6
1405.5
1465.6
1569.0
1616.5
1729.8
1850.9
1961.0

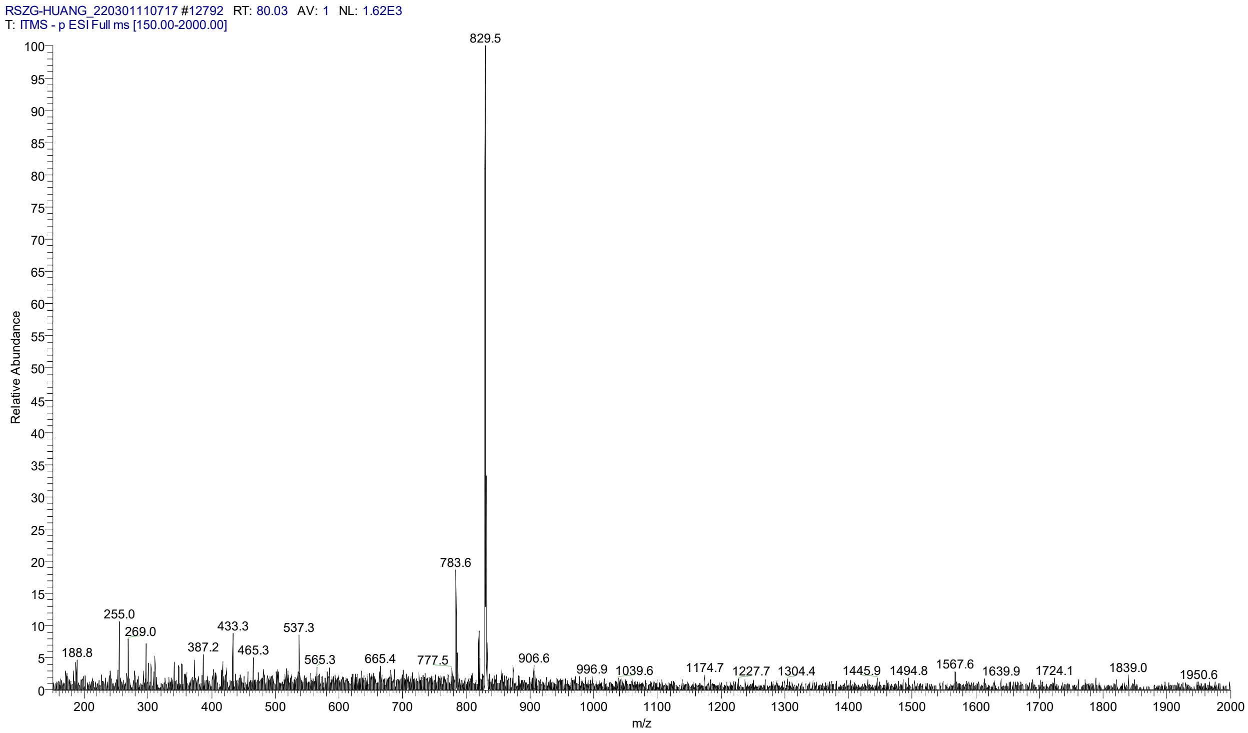
RSZG-HUANG_220301110717 #12792 RT: 80.03 AV: 1 NL: 1.62E3
T: ITMS - p ESI Full ms [150.00-2000.00]
Relative Abundance
m/z
829.5
783.6
255.0
269.0
188.8
387.2
433.3
465.3
537.3
565.3
665.4
777.5
906.6
996.9
1039.6
1174.7
1227.7
1304.4
1445.9
1494.8
1567.6
1639.9
1724.1
1839.0
1950.6

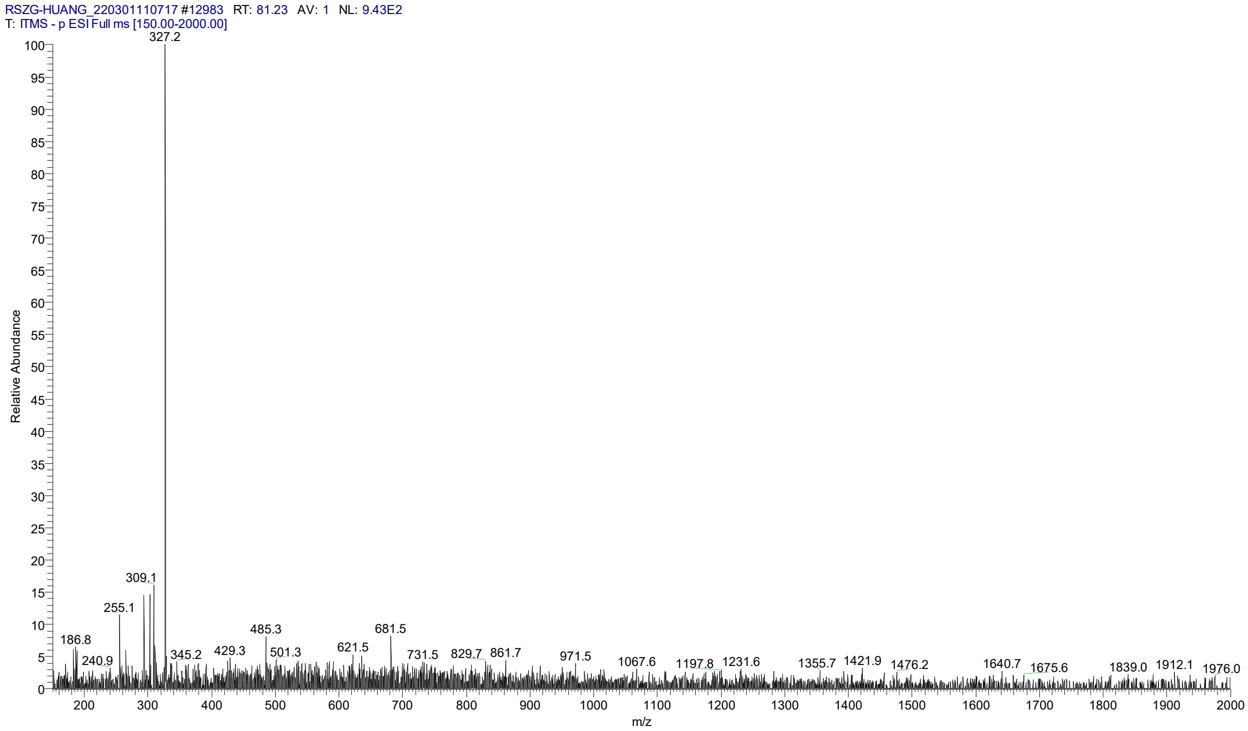
RSZG-HUANG_220301110717 #12983 RT: 81.23 AV: 1 NL: 9.43E2
T: ITMS - p ESI Full ms [150.00-2000.00]
Relative Abundance
m/z
327.2
309.1
255.1
186.8
240.9
345.2
429.3
485.3
501.3
621.5
681.5
731.5
829.7
861.7
971.5
1067.6
1197.8
1231.6
1355.7
1421.9
1476.2
1640.7
1675.6
1839.0
1912.1
1976.0

RSZG-HUANG_220301110717 #13079 RT: 81.83 AV: 1 NL: 1.76E3
T: ITMS - p ESI Full ms [150.00-2000.00]

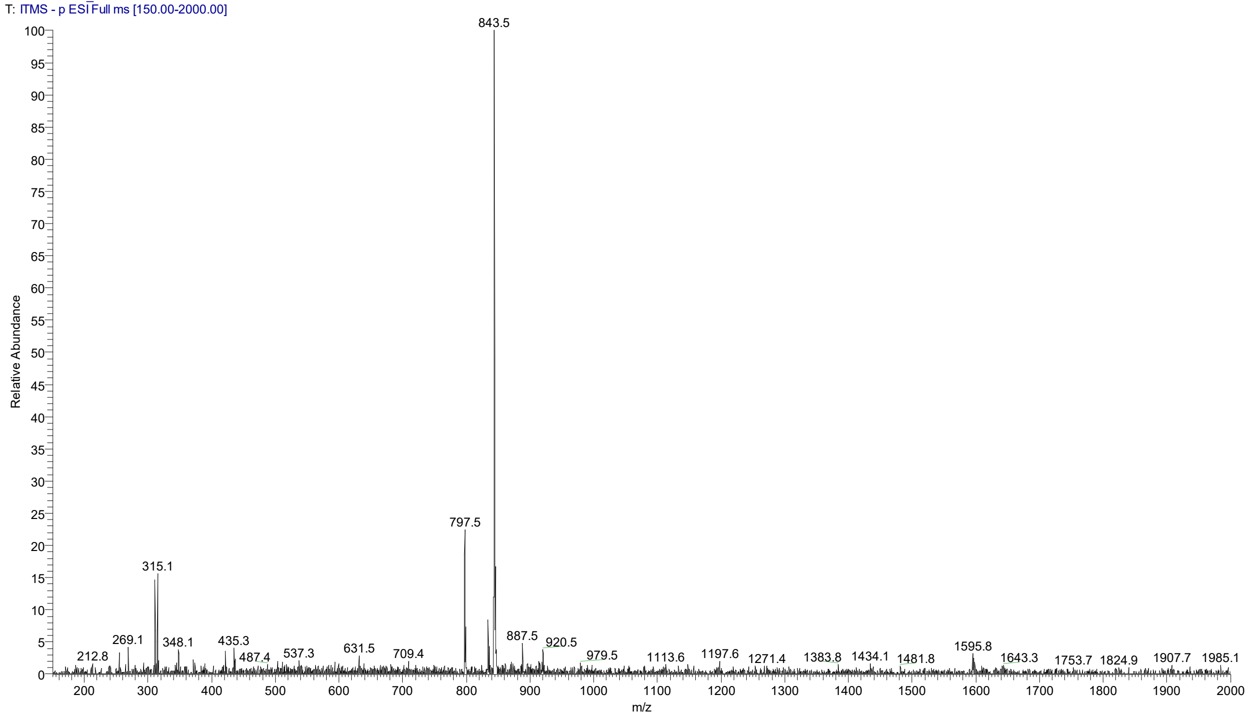

RSZG-HUANG_220301110717 #13881 RT: 86.85 AV: 1 NL: 3.93E3
T: ITMS - p ESI Full ms [150.00-2000.00]

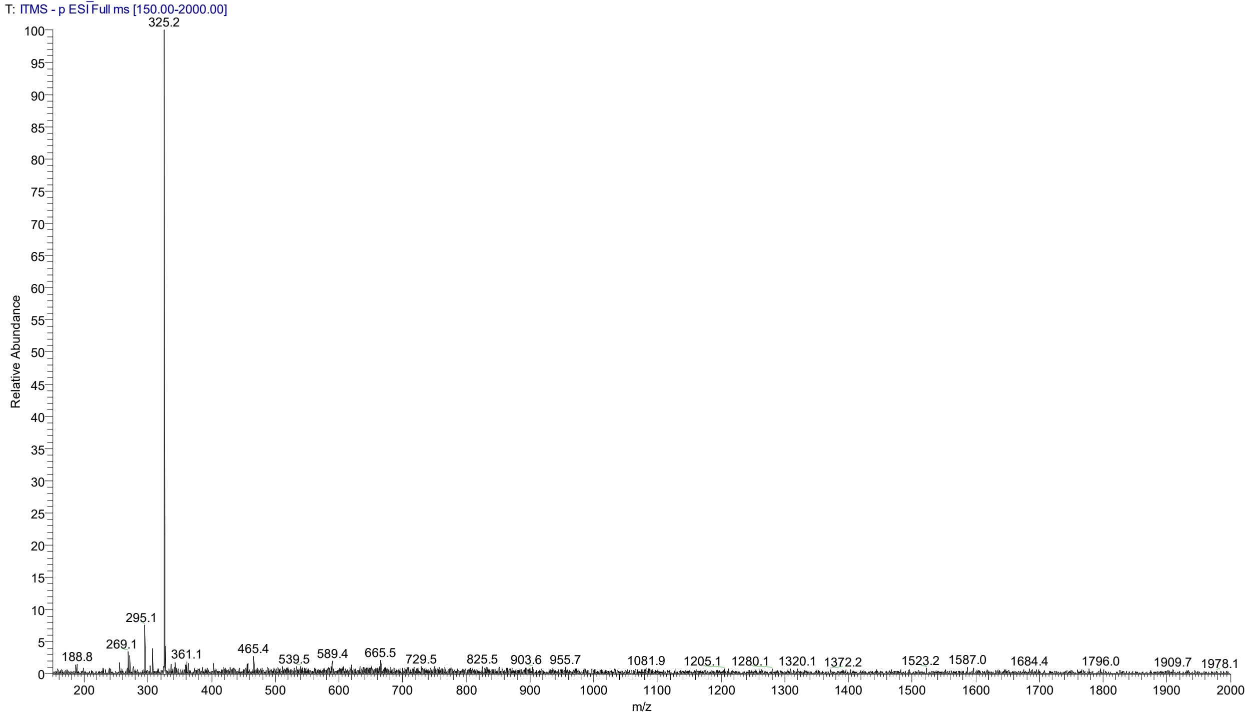

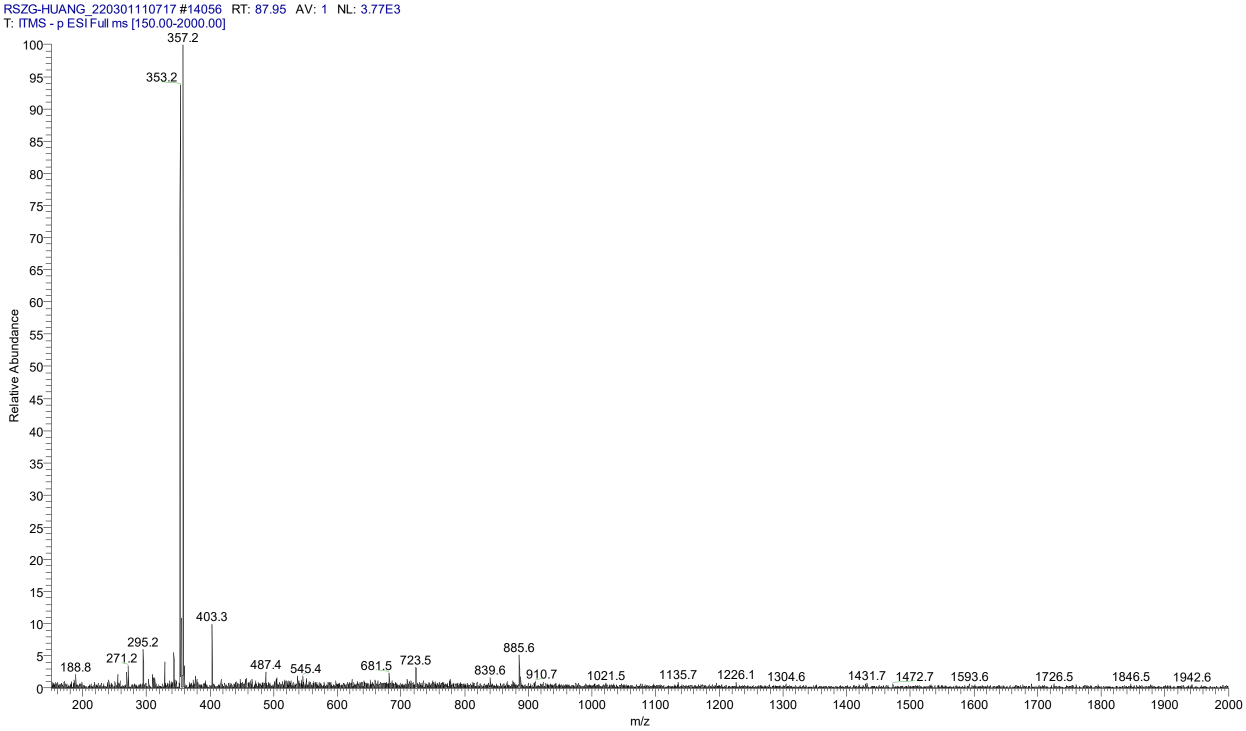
RSZG-HUANG_220301110717 #14056 RT: 87.95 AV: 1 NL: 3.77E3
T: ITMS - p ESI Full ms [150.00-2000.00]
357.2
353.2
403.3
295.2
271.2
188.8
487.4
545.4
681.5
723.5
885.6
839.6
910.7
1021.5
1135.7
1226.1
1304.6
1431.7
1472.7
1593.6
1726.5
1846.5
1942.6
Relative Abundance
m/z

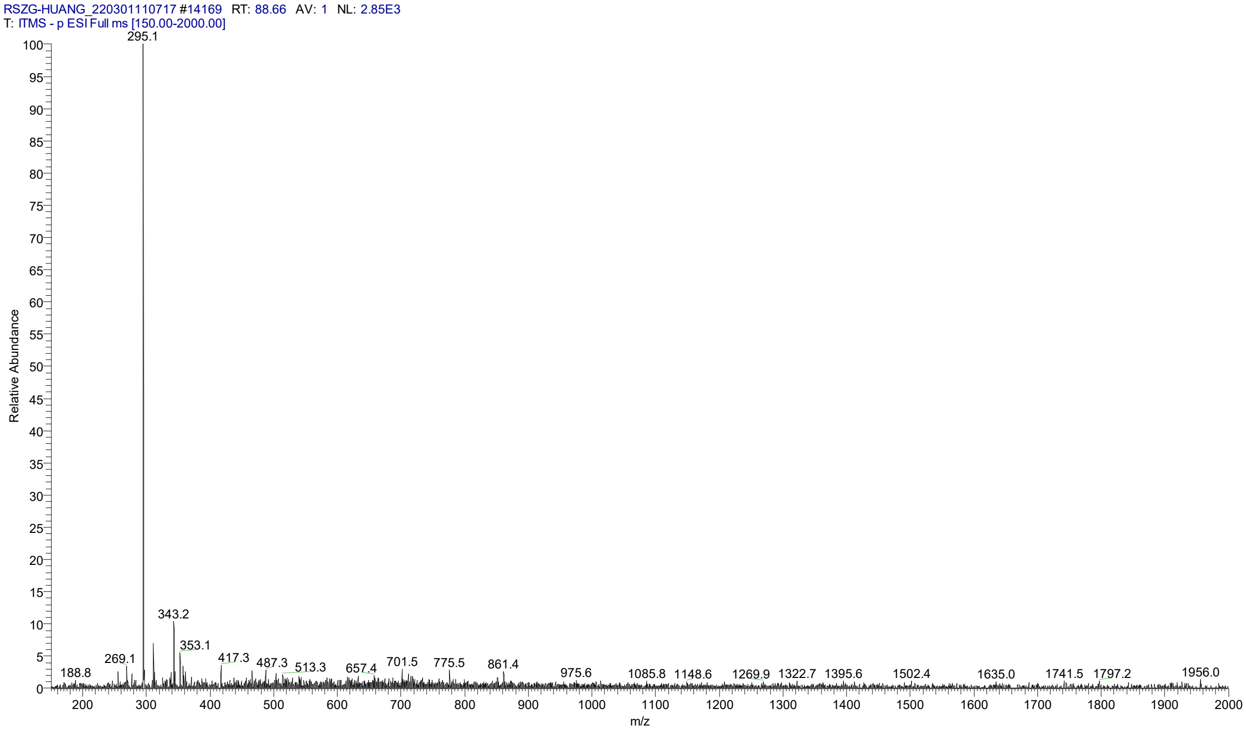
RSZG-HUANG_220301110717 #14169 RT: 88.66 AV: 1 NL: 2.85E3
T: ITMS - p ESI Full ms [150.00-2000.00]
295.1
343.2
353.1
188.8
269.1
417.3
487.3
513.3
657.4
701.5
775.5
861.4
975.6
1085.8
1148.6
1269.9
1322.7
1395.6
1502.4
1635.0
1741.5
1797.2
1956.0
Relative Abundance
m/z

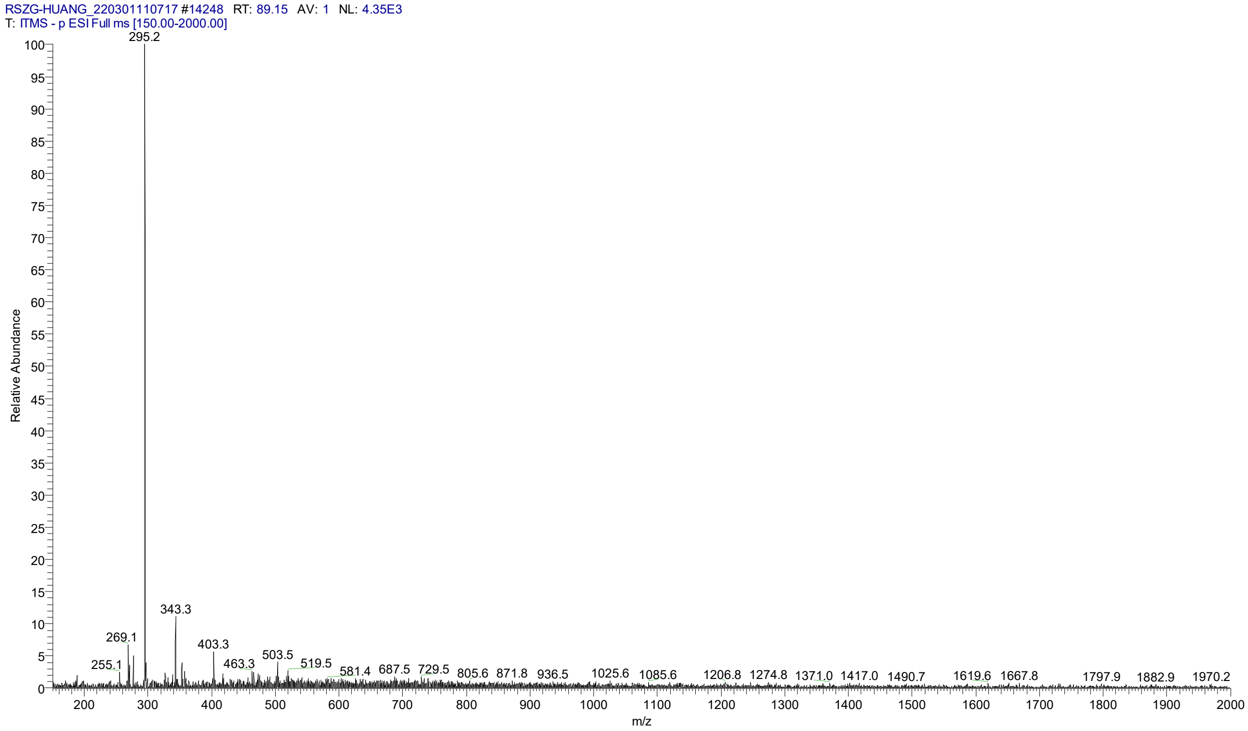
RSZG-HUANG_220301110717 #14248 RT: 89.15 AV: 1 NL: 4.35E3
T: ITMS - p ESI Full ms [150.00-2000.00]
Relative Abundance
m/z
295.2
343.3
269.1
403.3
255.1
463.3
503.5
519.5
581.4
687.5
729.5
805.6
871.8
936.5
1025.6
1085.6
1206.8
1274.8
1371.0
1417.0
1490.7
1619.6
1667.8
1797.9
1882.9
1970.2

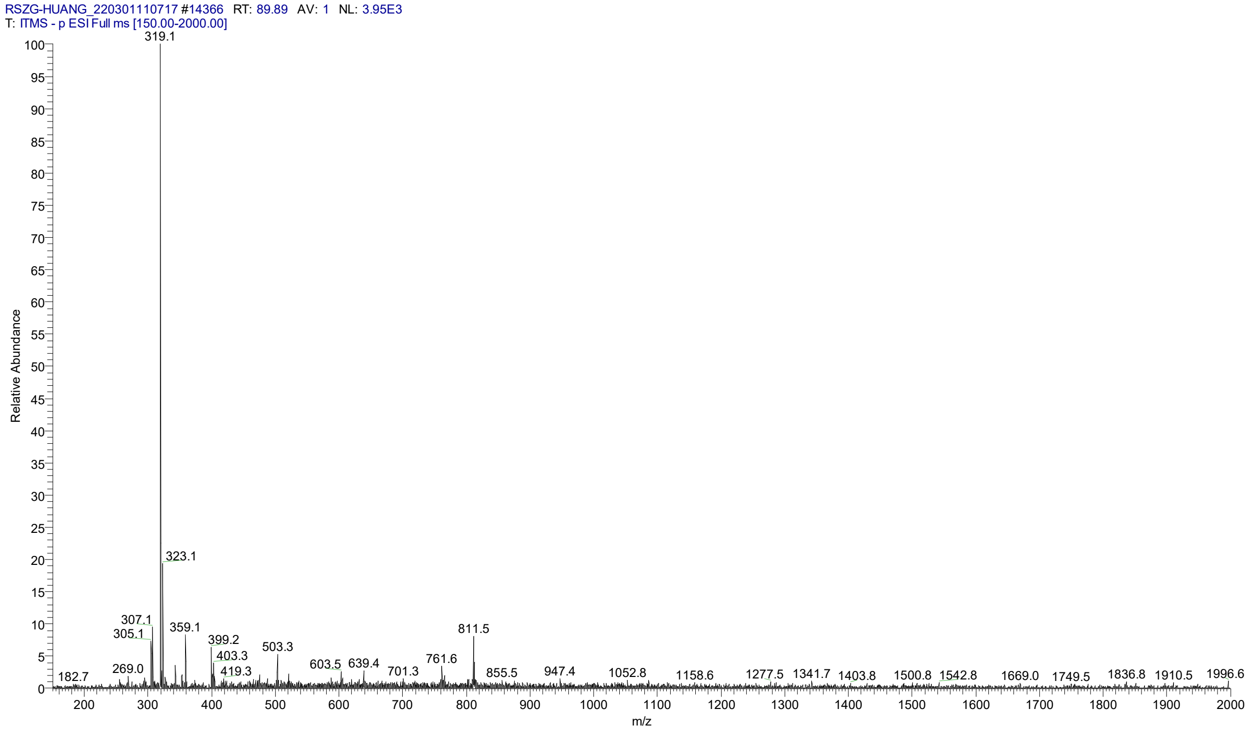
RSZG-HUANG_220301110717 #14366 RT: 89.89 AV: 1 NL: 3.95E3
T: ITMS - p ESI Full ms [150.00-2000.00]
Relative Abundance
m/z
319.1
323.1
307.1
305.1
359.1
399.2
403.3
419.3
503.3
811.5
761.6
182.7
269.0
603.5
639.4
701.3
855.5
947.4
1052.8
1158.6
1277.5
1341.7
1403.8
1500.8
1542.8
1669.0
1749.5
1836.8
1910.5
1996.6

RSZG-HUANG_220301110717 #14417 RT: 90.21 AV: 1 NL: 2.53E3
T: ITMS - p ESI Full ms [150.00-2000.00]

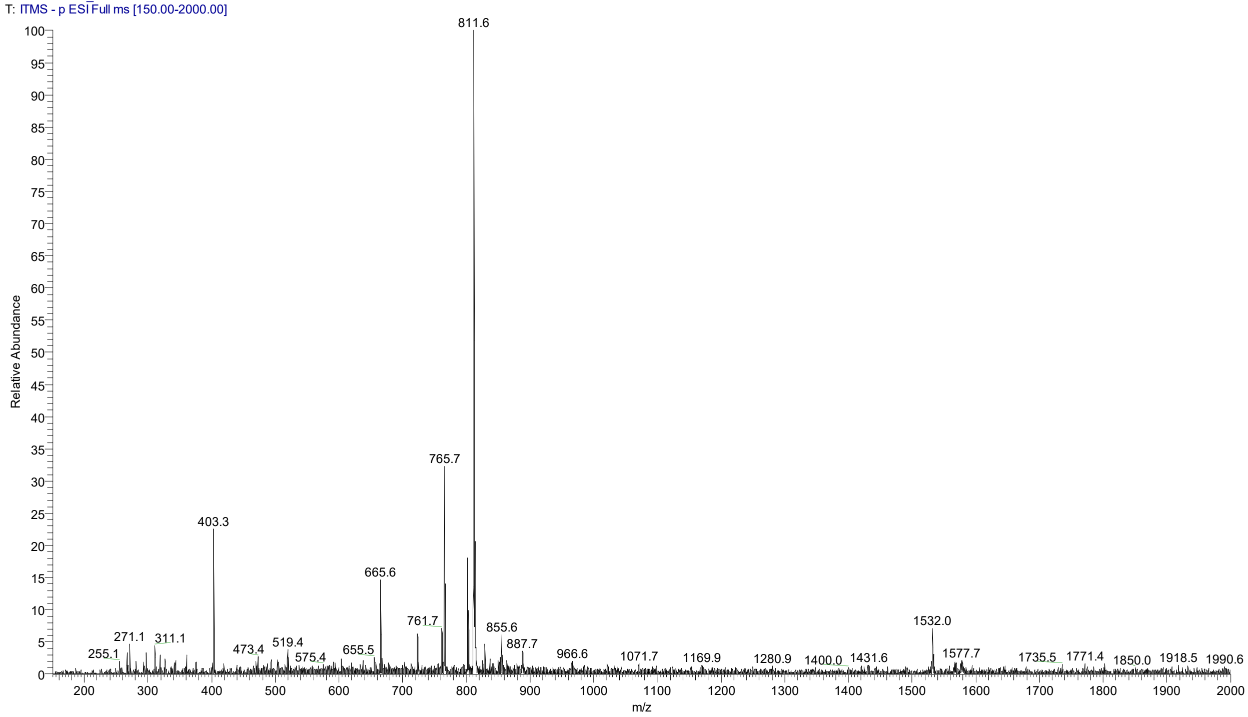

RSZG-HUANG_220301110717 #14541 RT: 90.99 AV: 1 NL: 2.30E3
T: ITMS - p ESI Full ms [150.00-2000.00]

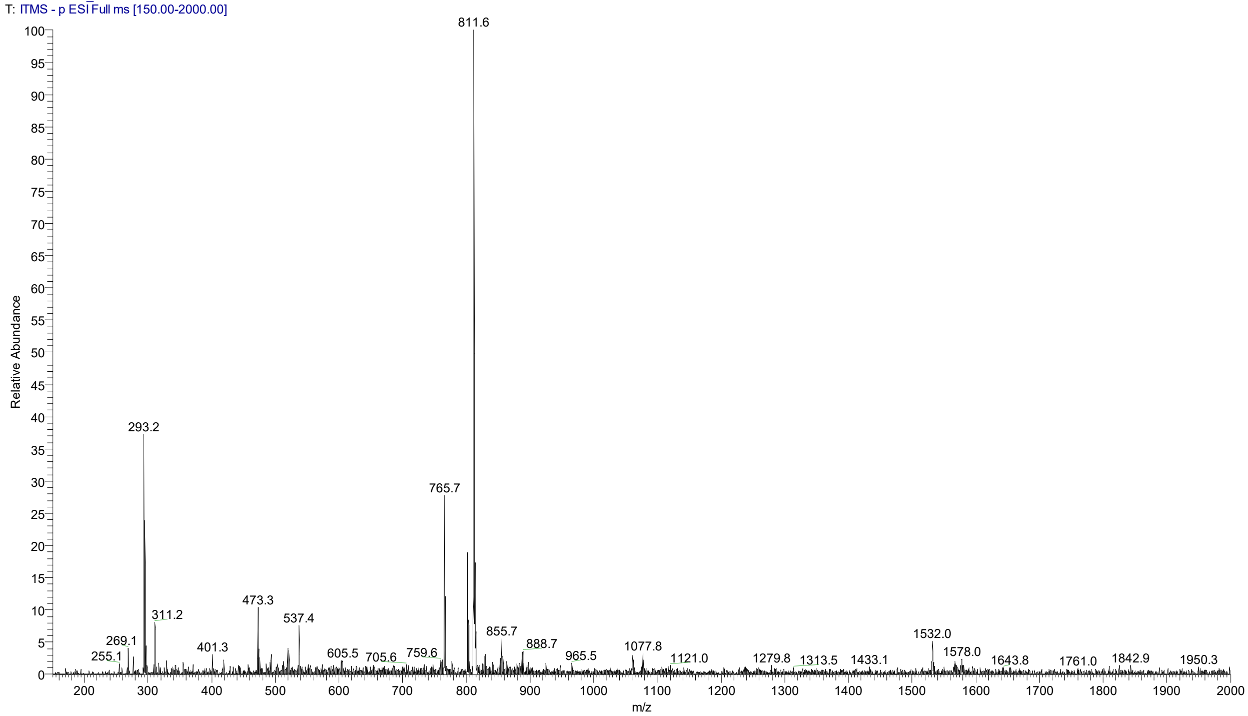

RSZG-HUANG_220301110717 #14631 RT: 91.55 AV: 1 NL: 1.17E3
T: ITMS - p ESI Full ms [150.00-2000.00]

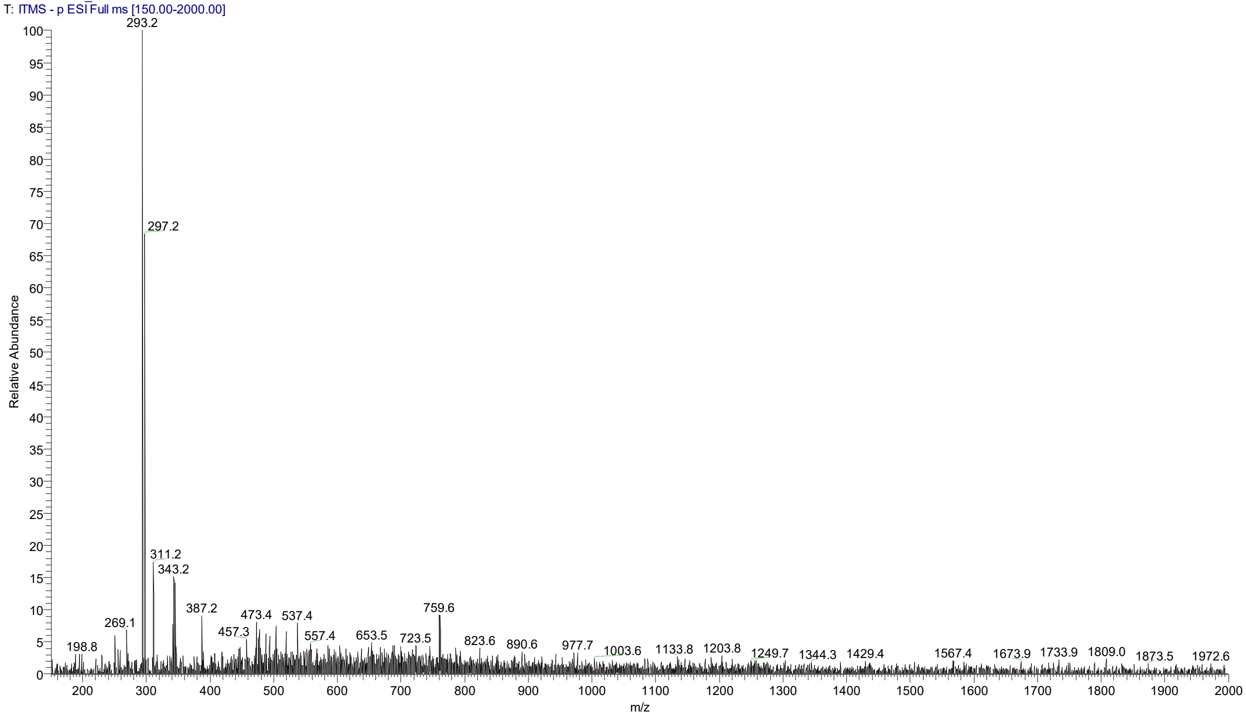

RSZG-HUANG_220301110717 #14693 RT: 91.94 AV: 1 NL: 7.77E2
T: ITMS - p ESI Full ms [150.00-2000.00]

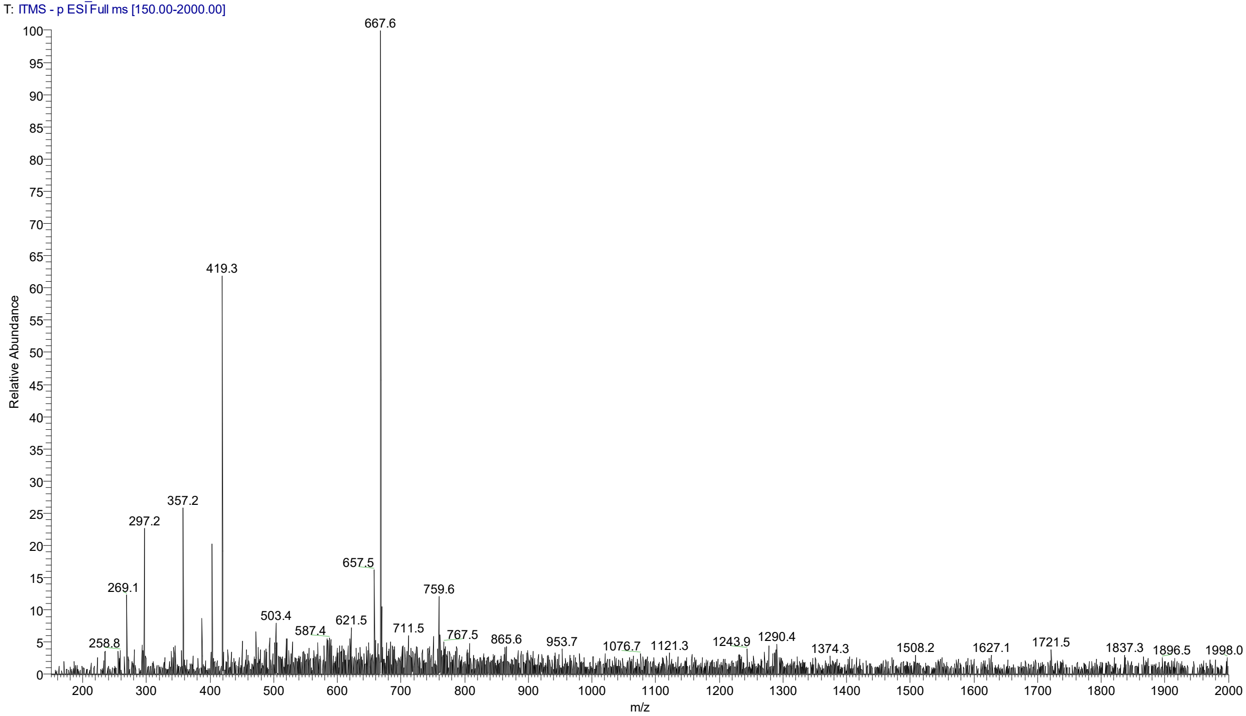

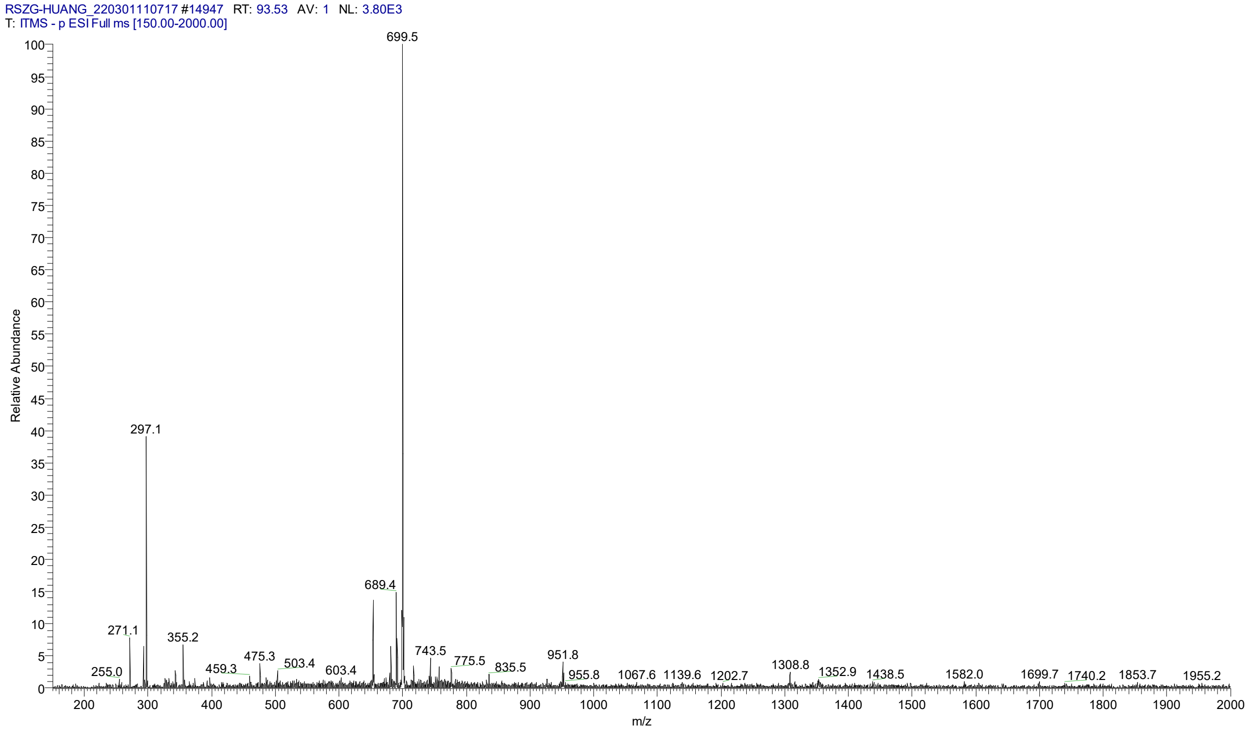
RSZG-HUANG_220301110717 #14947 RT: 93.53 AV: 1 NL: 3.80E3
T: ITMS - p ESI Full ms [150.00-2000.00]
Relative Abundance
m/z
699.5
297.1
689.4
271.1
355.2
255.0
459.3
475.3
503.4
603.4
743.5
775.5
835.5
951.8
955.8
1067.6
1139.6
1202.7
1308.8
1352.9
1438.5
1582.0
1699.7
1740.2
1853.7
1955.2
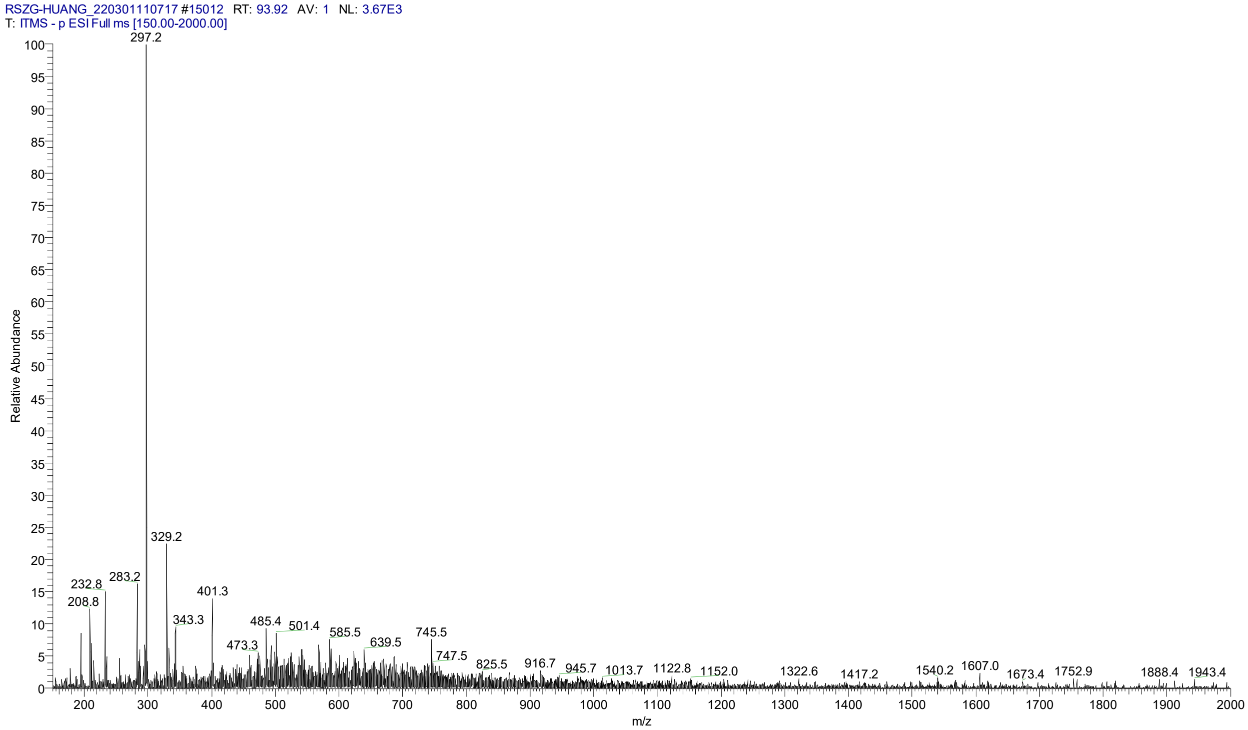
RSZG-HUANG_220301110717 #15012 RT: 93.92 AV: 1 NL: 3.67E3
T: ITMS - p ESI Full ms [150.00-2000.00]
Relative Abundance
m/z
297.2
329.2
232.8
283.2
208.8
401.3
343.3
485.4
501.4
473.3
585.5
639.5
745.5
747.5
825.5
916.7
945.7
1013.7
1122.8
1152.0
1322.6
1417.2
1540.2
1607.0
1673.4
1752.9
1888.4
1943.4

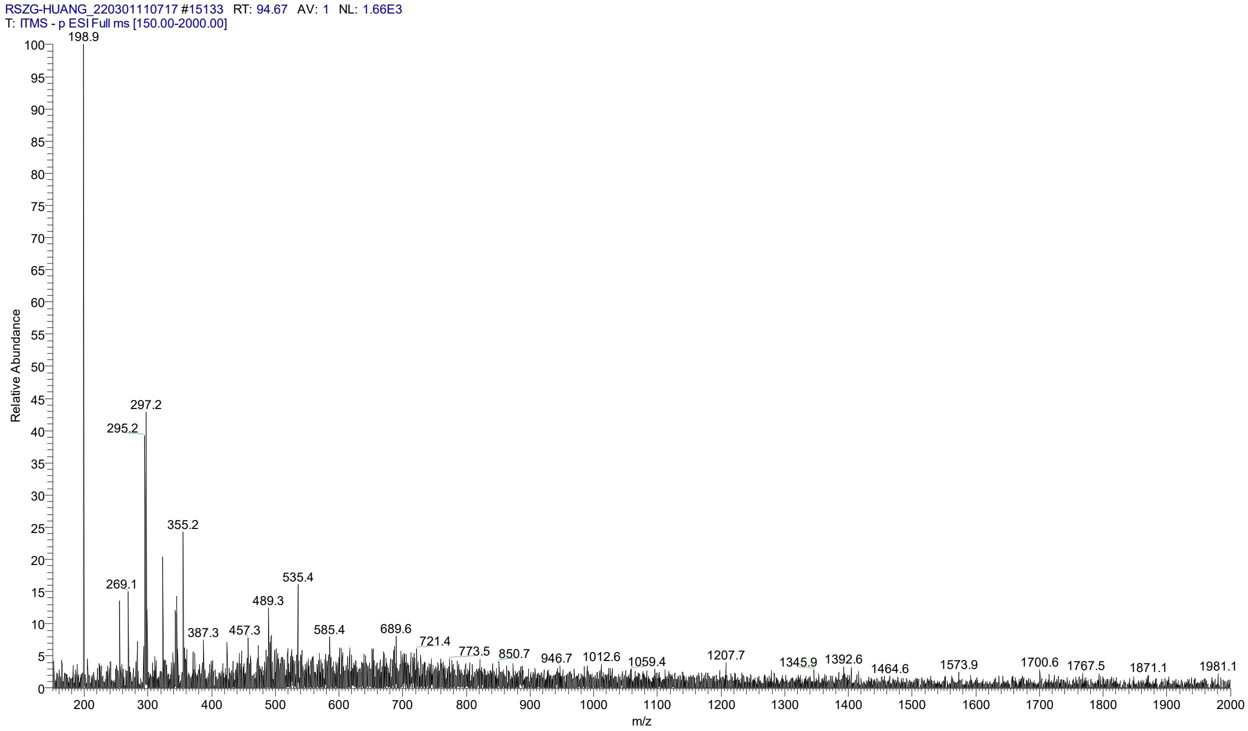
RSZG-HUANG_220301110717 #15133 RT: 94.67 AV: 1 NL: 1.66E3
T: ITMS - p ESI Full ms [150.00-2000.00]
Relative Abundance
m/z
198.9
297.2
295.2
355.2
269.1
535.4
489.3
387.3
457.3
585.4
689.6
721.4
773.5
850.7
946.7
1012.6
1059.4
1207.7
1345.9
1392.6
1464.6
1573.9
1700.6
1767.5
1871.1
1981.1

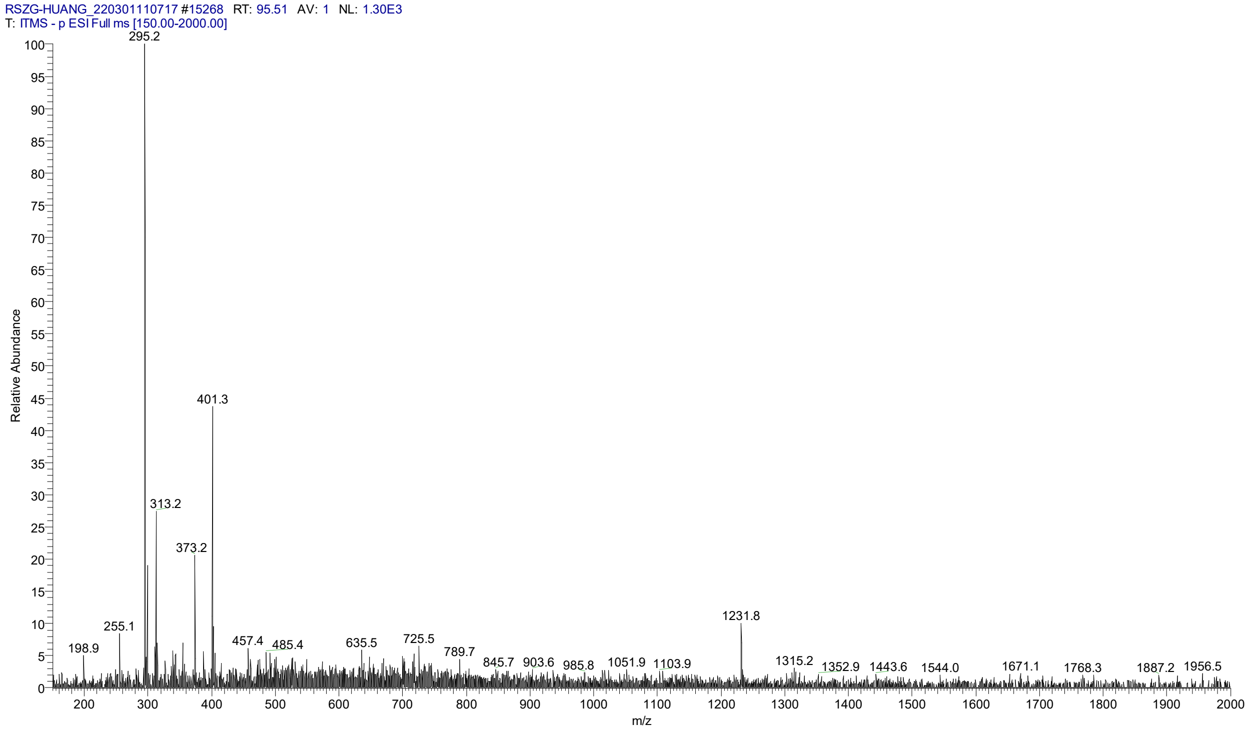
RSZG-HUANG_220301110717 #15268 RT: 95.51 AV: 1 NL: 1.30E3
T: ITMS - p ESI Full ms [150.00-2000.00]
Relative Abundance
m/z
295.2
401.3
313.2
373.2
255.1
198.9
457.4
485.4
635.5
725.5
789.7
845.7
903.6
985.8
1051.9
1103.9
1231.8
1315.2
1352.9
1443.6
1544.0
1671.1
1768.3
1887.2
1956.5

RSZG-HUANG_220301110717 #15381 RT: 96.22 AV: 1 NL: 1.98E3
T: ITMS - p ESI Full ms [150.00-2000.00]

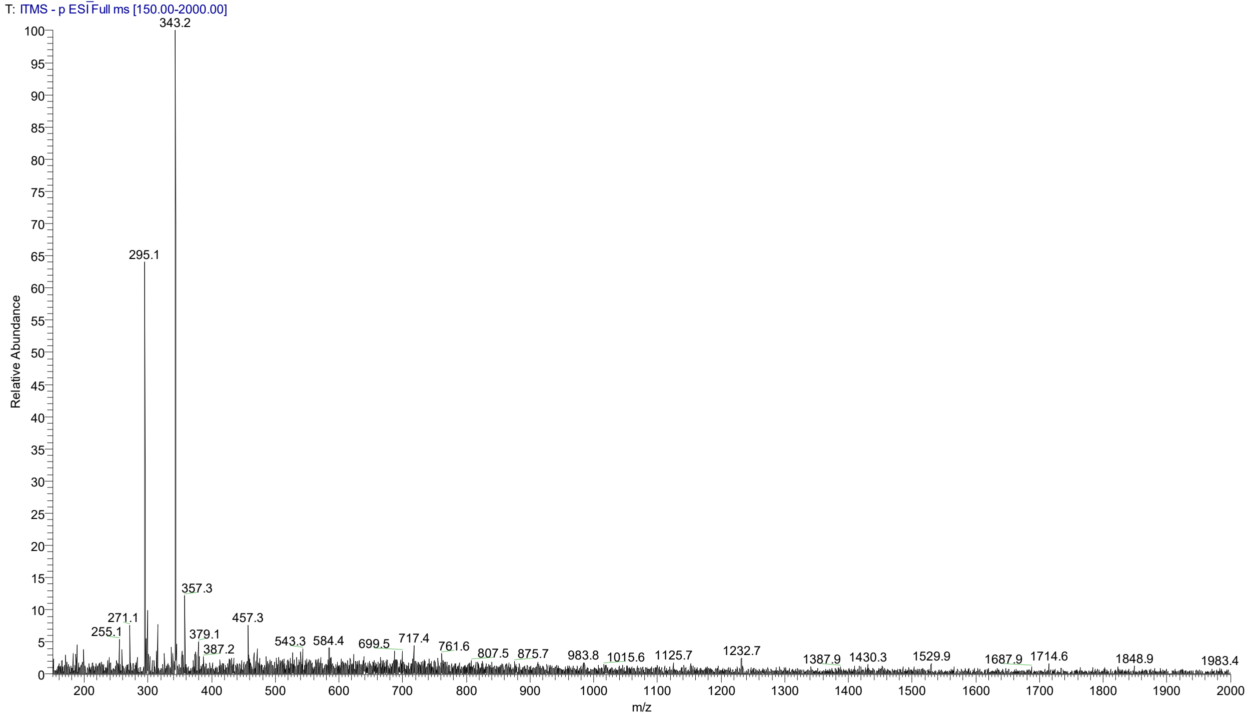

RSZG-HUANG_220301110717 #15460 RT: 96.72 AV: 1 NL: 5.41E3
T: ITMS - p ESI Full ms [150.00-2000.00]

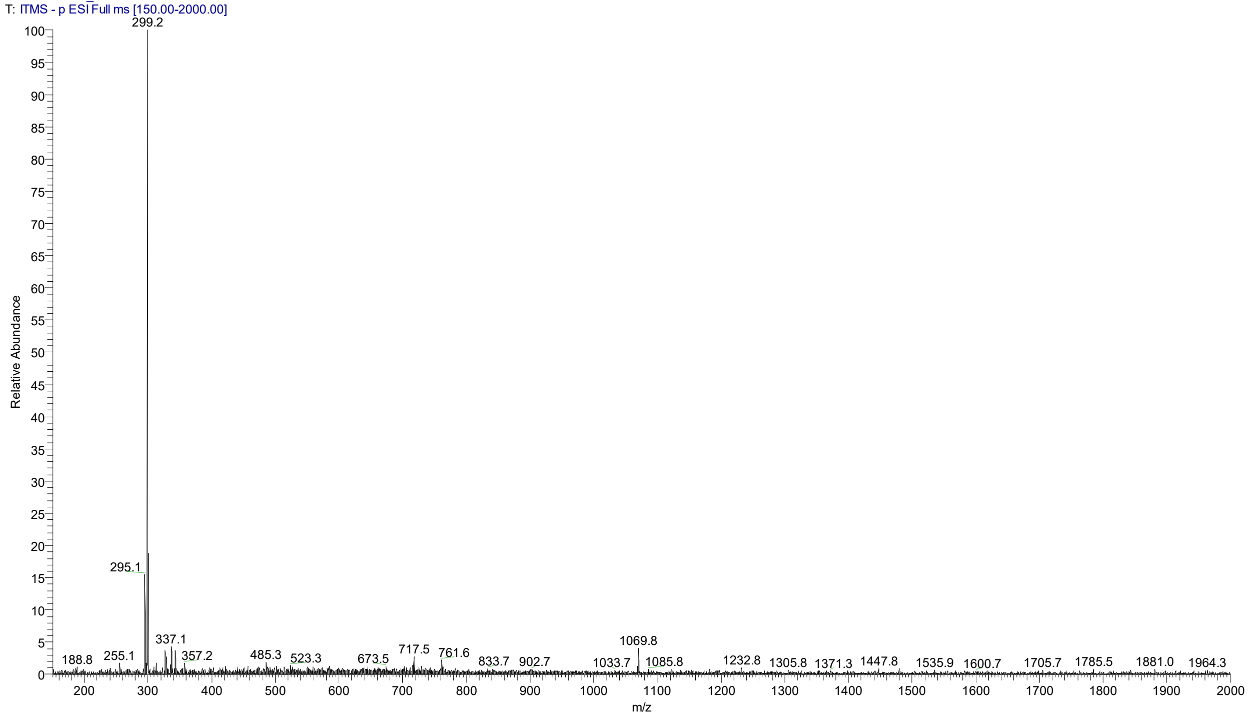

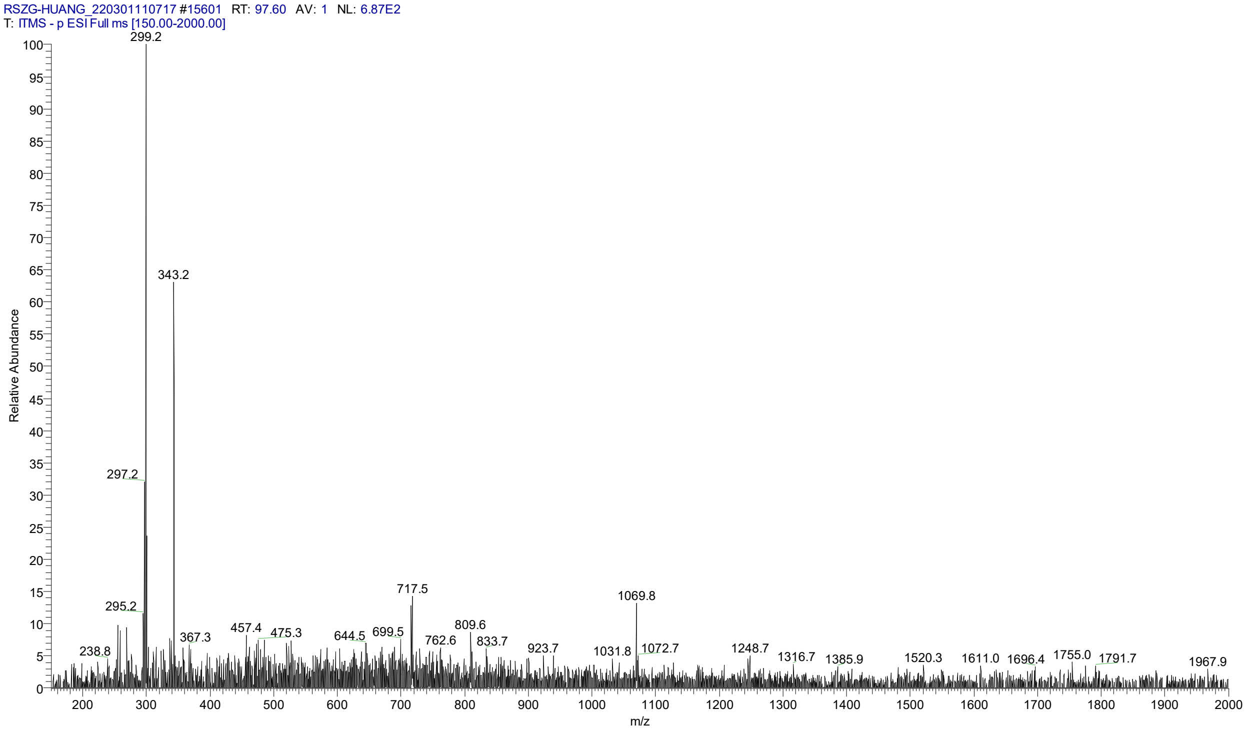
RSZG-HUANG_220301110717 #15601 RT: 97.60 AV: 1 NL: 6.87E2
T: ITMS - p ESI Full ms [150.00-2000.00]
Relative Abundance
m/z
299.2
343.2
297.2
295.2
238.8
367.3
457.4
475.3
644.5
699.5
717.5
762.6
809.6
833.7
923.7
1031.8
1069.8
1072.7
1248.7
1316.7
1385.9
1520.3
1611.0
1696.4
1755.0
1791.7
1967.9

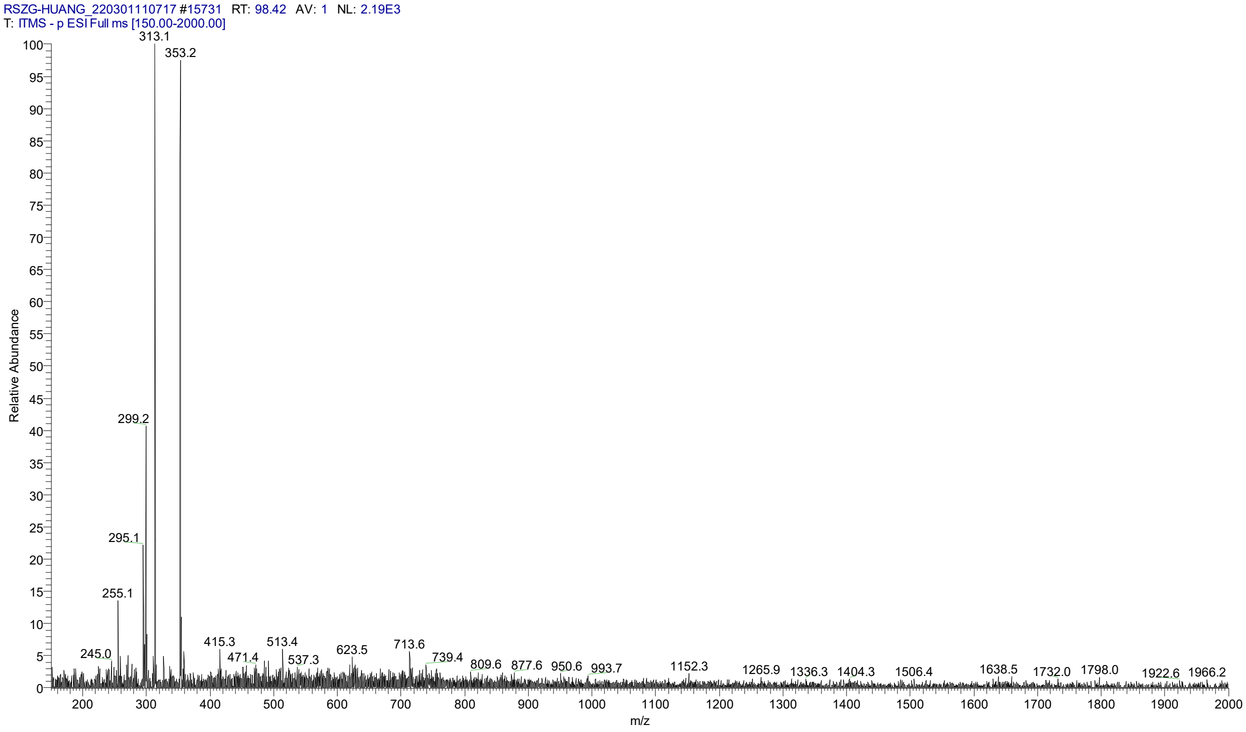
RSZG-HUANG_220301110717 #15731 RT: 98.42 AV: 1 NL: 2.19E3
T: ITMS - p ESI Full ms [150.00-2000.00]
Relative Abundance
m/z
313.1
353.2
299.2
295.1
255.1
245.0
415.3
471.4
513.4
537.3
623.5
713.6
739.4
809.6
877.6
950.6
993.7
1152.3
1265.9
1336.3
1404.3
1506.4
1638.5
1732.0
1798.0
1922.6
1966.2

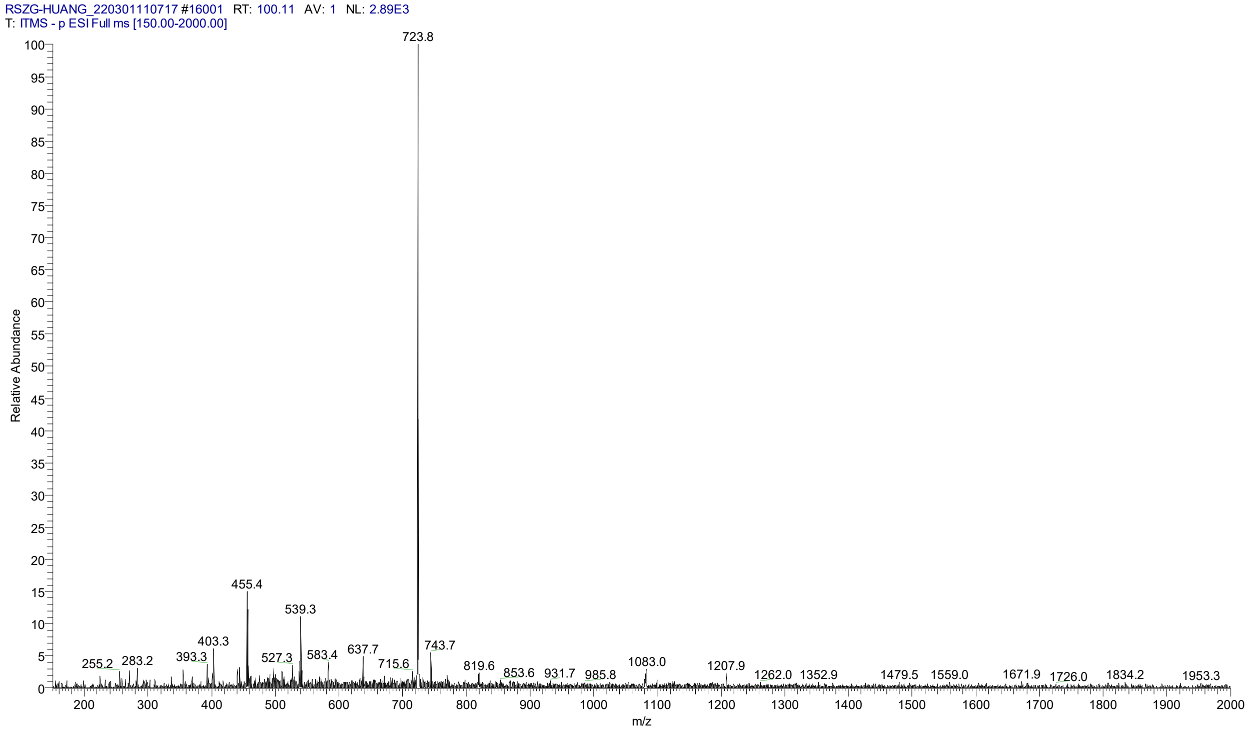
RSZG-HUANG_220301110717 #16001 RT: 100.11 AV: 1 NL: 2.89E3
T: ITMS - p ESI Full ms [150.00-2000.00]
Relative Abundance
m/z
723.8
455.4
539.3
403.3
743.7
637.7
583.4
527.3
393.3
283.2
255.2
715.6
819.6
853.6
931.7
985.8
1083.0
1207.9
1262.0
1352.9
1479.5
1559.0
1671.9
1726.0
1834.2
1953.3

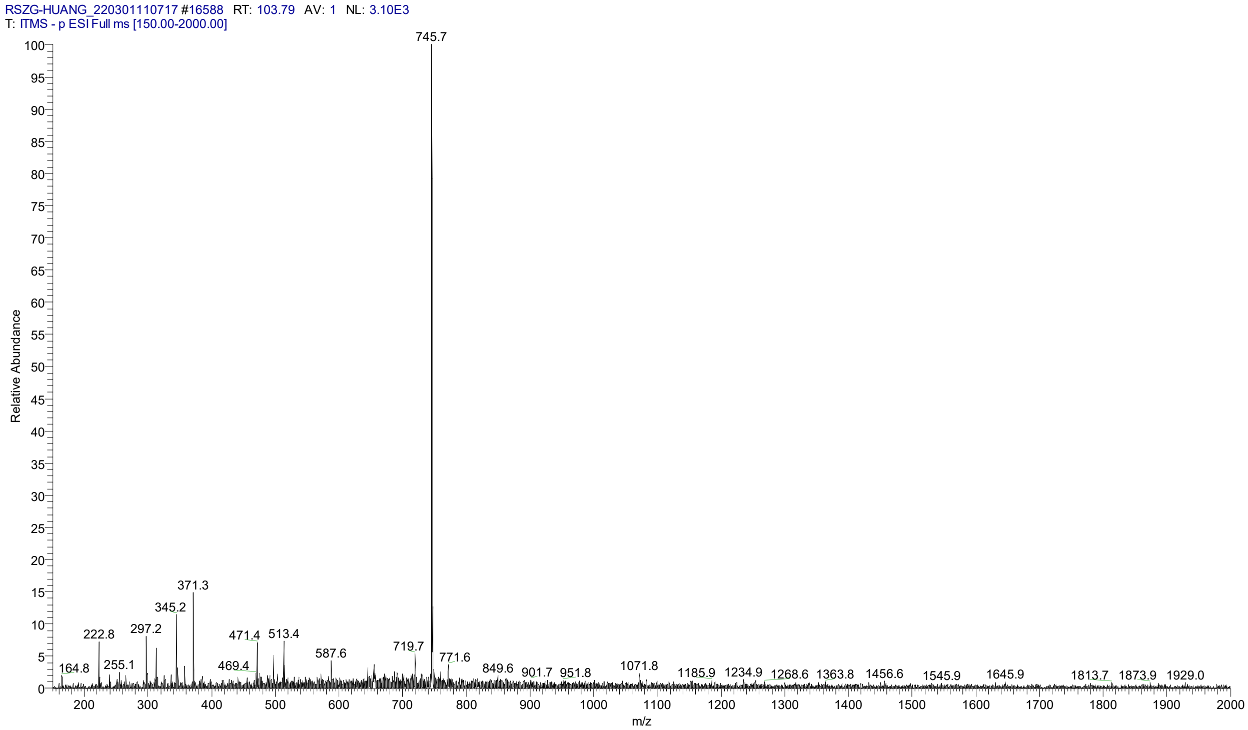
RSZG-HUANG_220301110717 #16588 RT: 103.79 AV: 1 NL: 3.10E3
T: ITMS - p ESI Full ms [150.00-2000.00]
Relative Abundance
m/z
745.7
371.3
345.2
297.2
222.8
471.4
513.4
587.6
719.7
771.6
164.8
255.1
469.4
849.6
901.7
951.8
1071.8
1185.9
1234.9
1268.6
1363.8
1456.6
1545.9
1645.9
1813.7
1873.9
1929.0

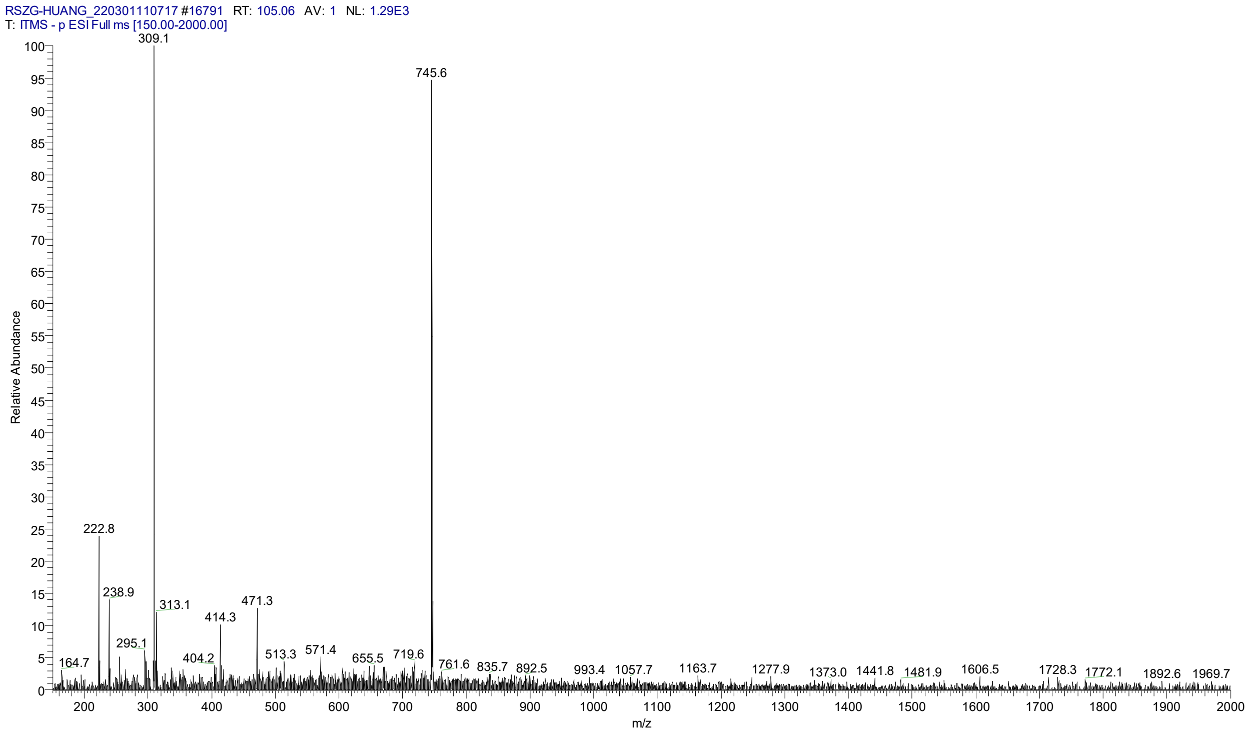
RSZG-HUANG_220301110717 #16791 RT: 105.06 AV: 1 NL: 1.29E3
T: ITMS - p ESI Full ms [150.00-2000.00]
Relative Abundance
m/z
309.1
745.6
222.8
238.9
313.1
471.3
414.3
295.1
164.7
404.2
513.3
571.4
655.5
719.6
761.6
835.7
892.5
993.4
1057.7
1163.7
1277.9
1373.0
1441.8
1481.9
1606.5
1728.3
1772.1
1892.6
1969.7

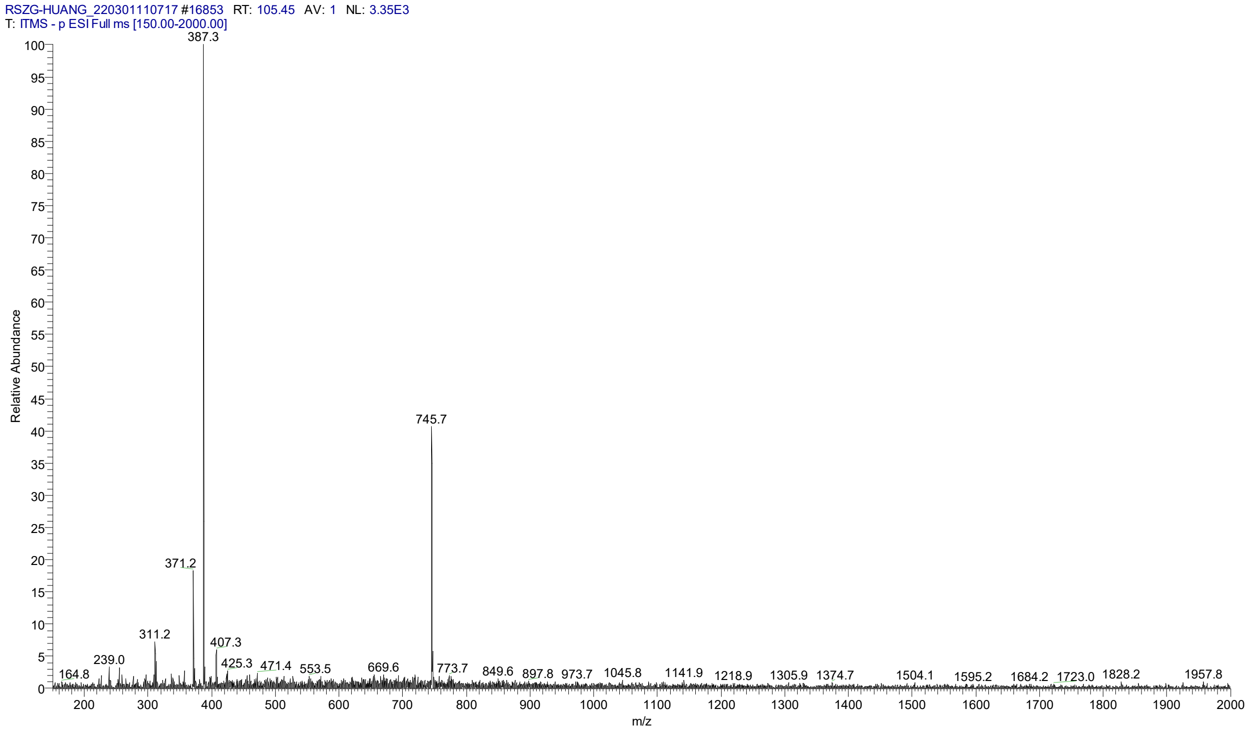
RSZG-HUANG_220301110717 #16853 RT: 105.45 AV: 1 NL: 3.35E3
T: ITMS - p ESI Full ms [150.00-2000.00]
Relative Abundance
m/z
387.3
745.7
371.2
311.2
407.3
239.0
164.8
425.3
471.4
553.5
669.6
773.7
849.6
897.8
973.7
1045.8
1141.9
1218.9
1305.9
1374.7
1504.1
1595.2
1684.2
1723.0
1828.2
1957.8

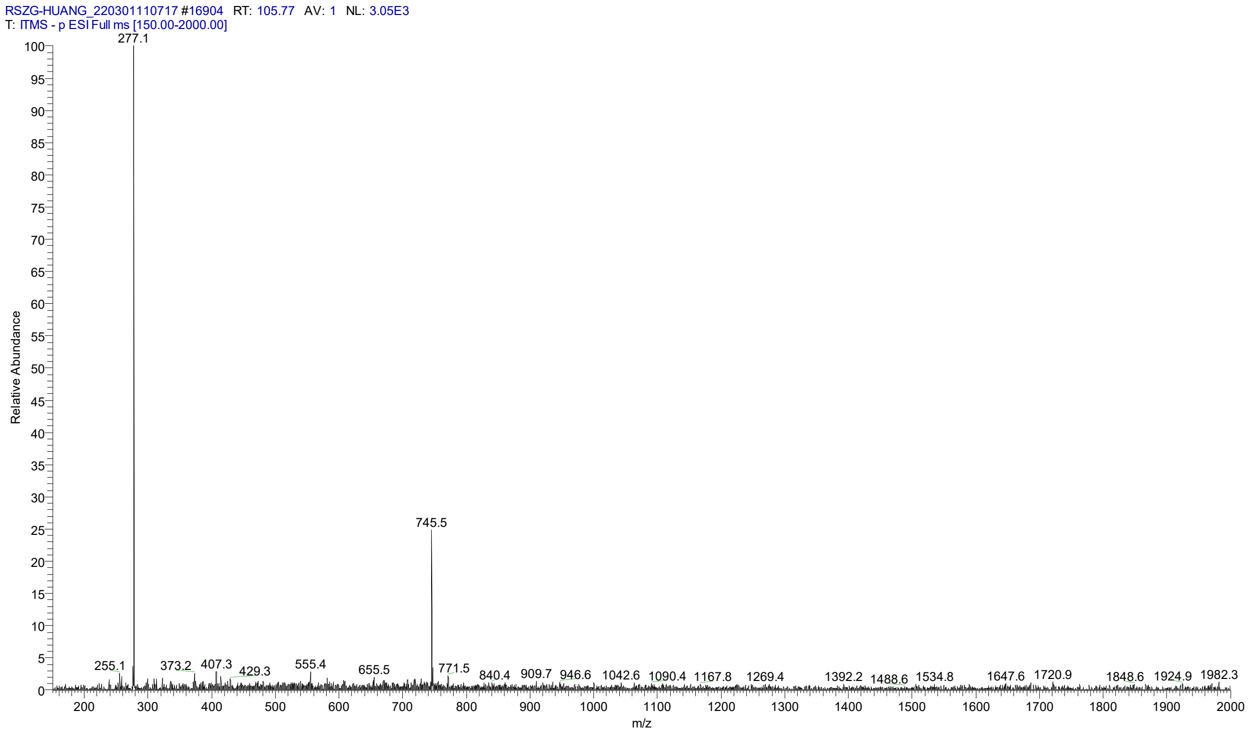
RSZG-HUANG_220301110717 #16904 RT: 105.77 AV: 1 NL: 3.05E3
T: ITMS - p ESI Full ms [150.00-2000.00]
277.1
745.5
255.1
373.2
407.3
429.3
555.4
655.5
771.5
840.4
909.7
946.6
1042.6
1090.4
1167.8
1269.4
1392.2
1488.6
1534.8
1647.6
1720.9
1848.6
1924.9
1982.3
Relative Abundance
m/z

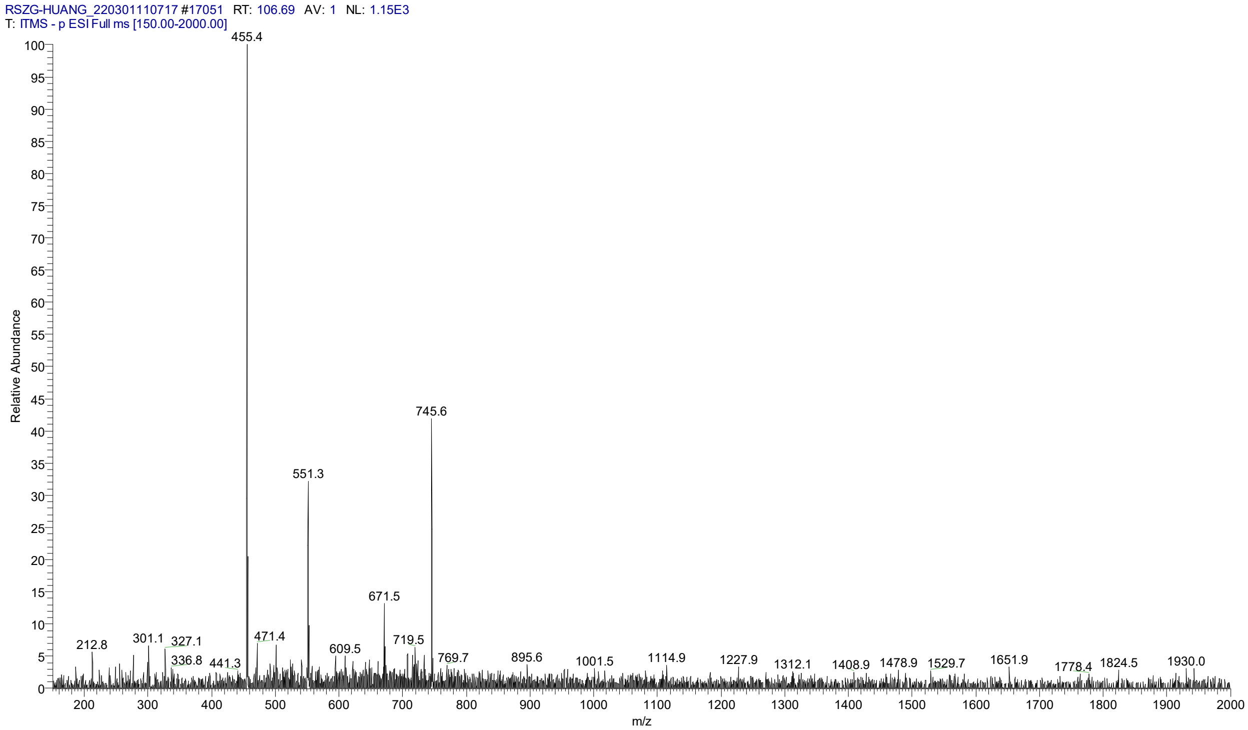
RSZG-HUANG_220301110717 #17051 RT: 106.69 AV: 1 NL: 1.15E3
T: ITMS - p ESI Full ms [150.00-2000.00]
455.4
745.6
551.3
671.5
212.8
301.1
327.1
336.8
441.3
471.4
609.5
719.5
769.7
895.6
1001.5
1114.9
1227.9
1312.1
1408.9
1478.9
1529.7
1651.9
1778.4
1824.5
1930.0
Relative Abundance
m/z

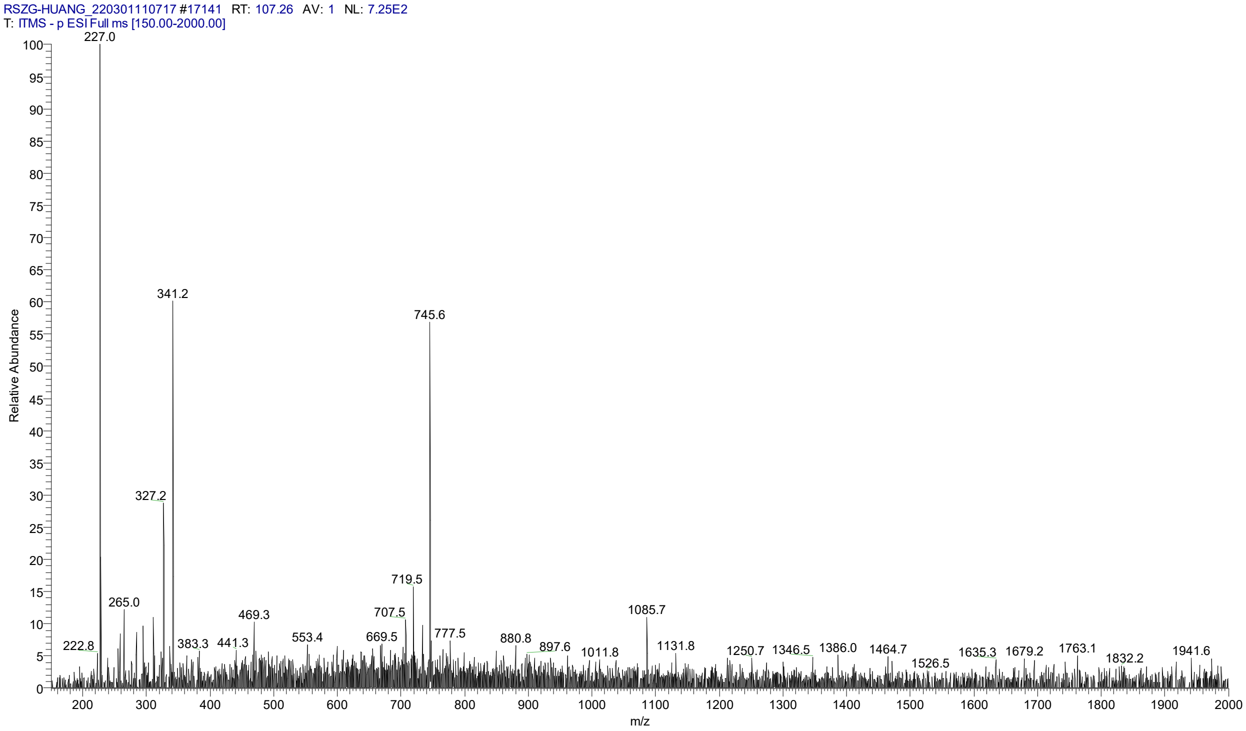
RSZG-HUANG_220301110717 #17141 RT: 107.26 AV: 1 NL: 7.25E2
T: ITMS - p ESI Full ms [150.00-2000.00]
Relative Abundance
m/z
227.0
341.2
745.6
327.2
719.5
265.0
469.3
707.5
1085.7
222.8
383.3
441.3
553.4
669.5
777.5
880.8
897.6
1011.8
1131.8
1250.7
1346.5
1386.0
1464.7
1526.5
1635.3
1679.2
1763.1
1832.2
1941.6

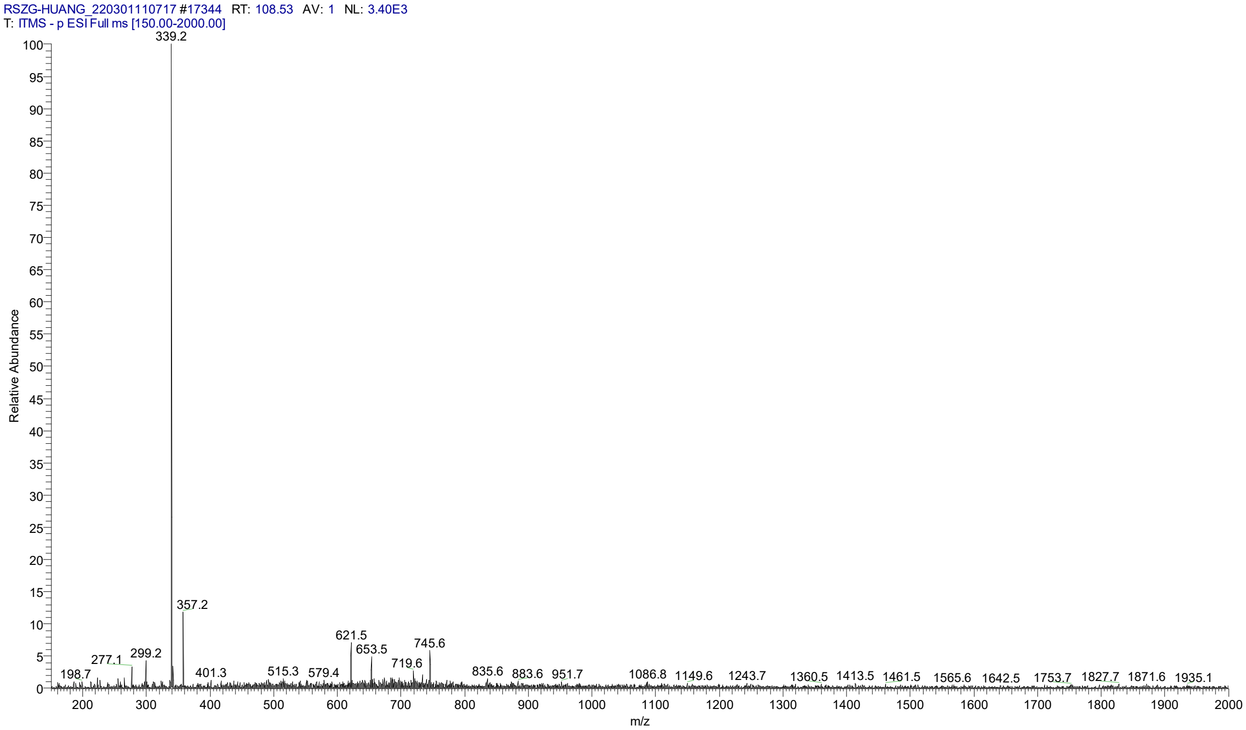
RSZG-HUANG_220301110717 #17344 RT: 108.53 AV: 1 NL: 3.40E3
T: ITMS - p ESI Full ms [150.00-2000.00]
Relative Abundance
m/z
339.2
357.2
621.5
653.5
745.6
719.6
299.2
277.1
198.7
401.3
515.3
579.4
835.6
883.6
951.7
1086.8
1149.6
1243.7
1360.5
1413.5
1461.5
1565.6
1642.5
1753.7
1827.7
1871.6
1935.1

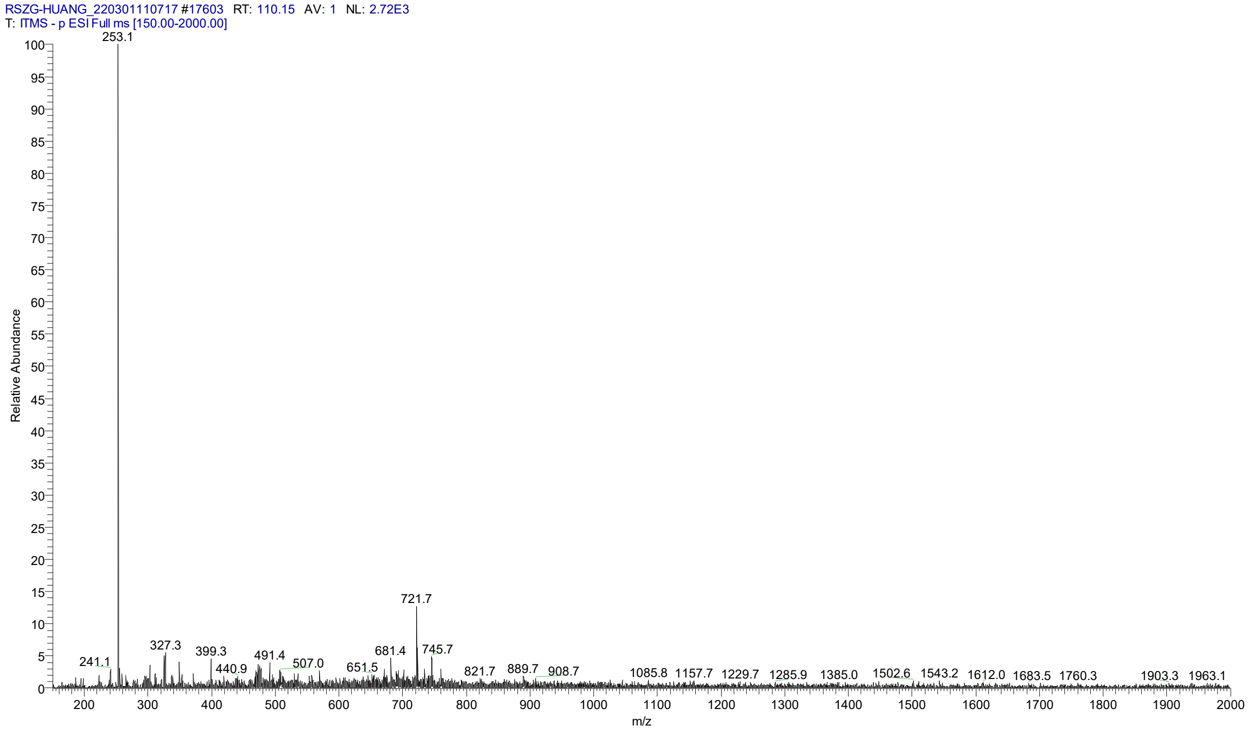
RSZG-HUANG_220301110717 #17603 RT: 110.15 AV: 1 NL: 2.72E3
T: ITMS - p ESI Full ms [150.00-2000.00]
Relative Abundance
m/z
253.1
721.7
327.3
399.3
491.4
681.4
745.7
241.1
440.9
507.0
651.5
821.7
889.7
908.7
1085.8
1157.7
1229.7
1285.9
1385.0
1502.6
1543.2
1612.0
1683.5
1760.3
1903.3
1963.1

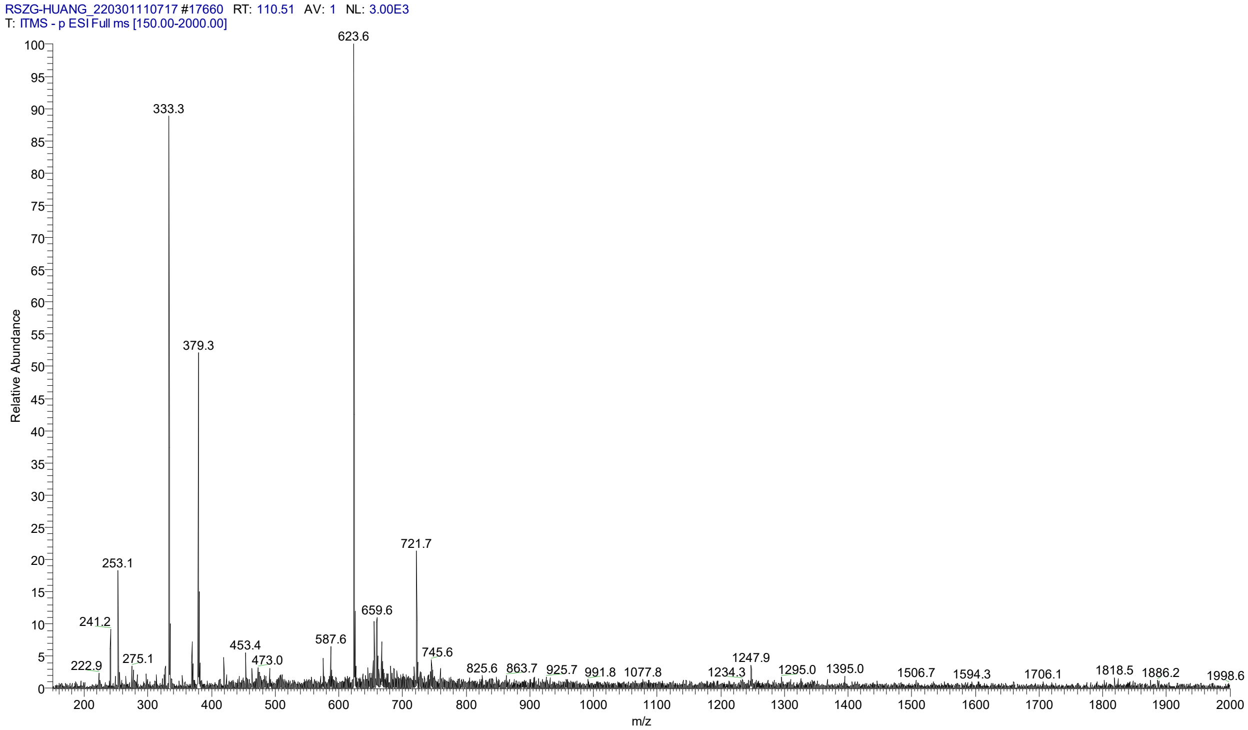
RSZG-HUANG_220301110717 #17660 RT: 110.51 AV: 1 NL: 3.00E3
T: ITMS - p ESI Full ms [150.00-2000.00]
Relative Abundance
m/z
623.6
333.3
379.3
253.1
721.7
659.6
241.2
587.6
453.4
745.6
222.9
275.1
473.0
825.6
863.7
925.7
991.8
1077.8
1234.3
1247.9
1295.0
1395.0
1506.7
1594.3
1706.1
1818.5
1886.2
1998.6

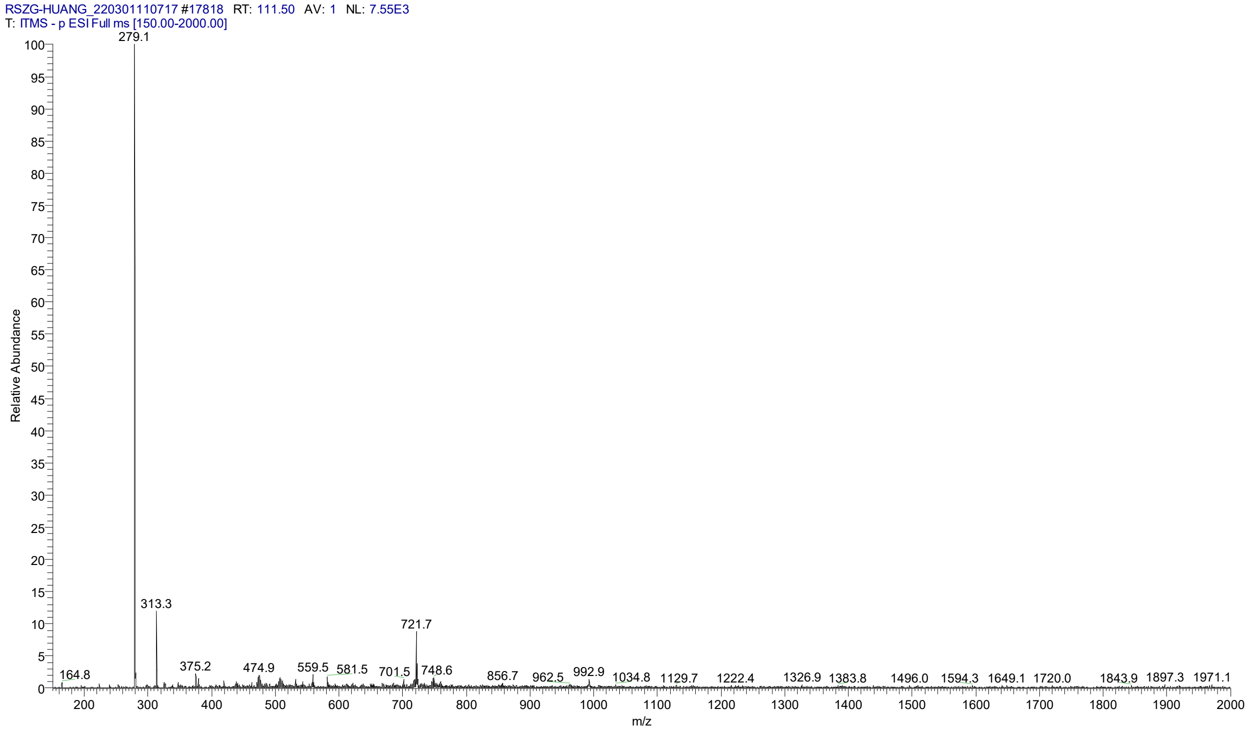
RSZG-HUANG_220301110717 #17818 RT: 111.50 AV: 1 NL: 7.55E3
T: ITMS - p ESI Full ms [150.00-2000.00]
Relative Abundance
m/z
279.1
313.3
721.7
375.2
474.9
559.5
581.5
701.5
748.6
164.8
856.7
962.5
992.9
1034.8
1129.7
1222.4
1326.9
1383.8
1496.0
1594.3
1649.1
1720.0
1843.9
1897.3
1971.1

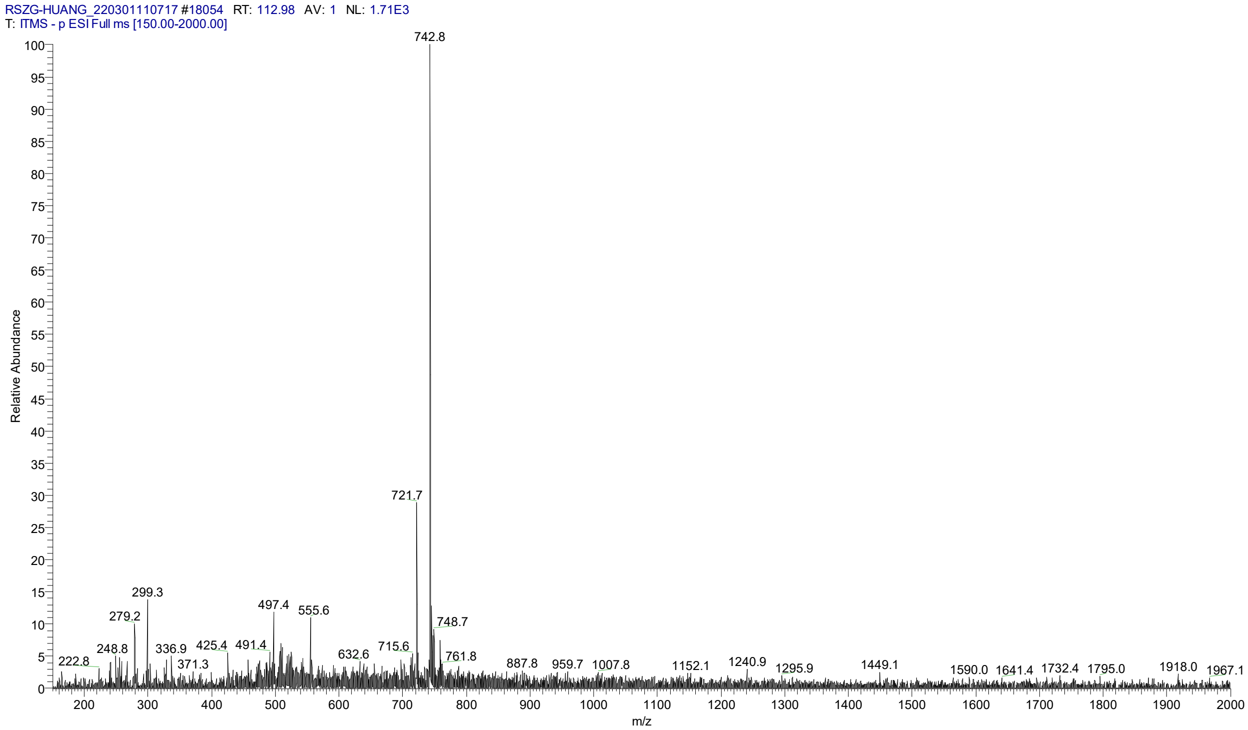
RSZG-HUANG_220301110717 #18054 RT: 112.98 AV: 1 NL: 1.71E3
T: ITMS - p ESI Full ms [150.00-2000.00]
Relative Abundance
m/z
742.8
721.7
299.3
279.2
497.4
555.6
748.7
248.8
222.8
336.9
371.3
425.4
491.4
632.6
715.6
761.8
887.8
959.7
1007.8
1152.1
1240.9
1295.9
1449.1
1590.0
1641.4
1732.4
1795.0
1918.0
1967.1

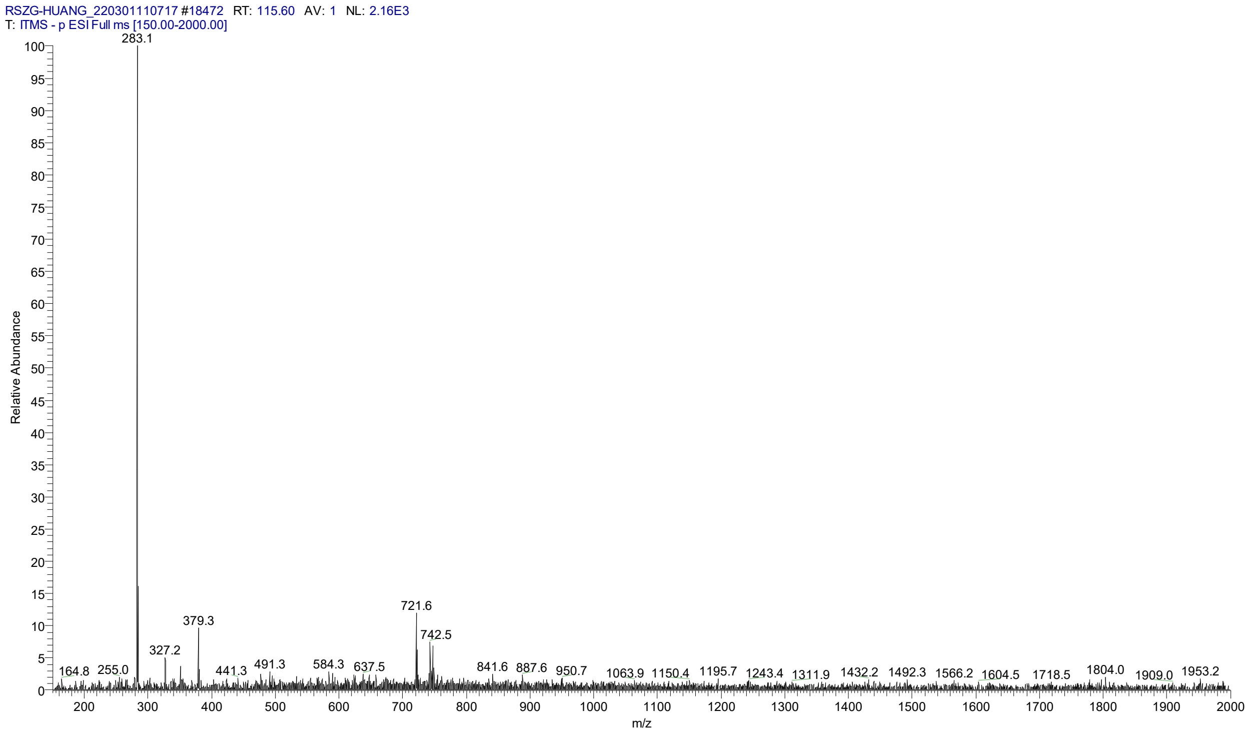
RSZG-HUANG_220301110717 #18472 RT: 115.60 AV: 1 NL: 2.16E3
T: ITMS - p ESI Full ms [150.00-2000.00]
Relative Abundance
m/z
283.1
164.8
255.0
327.2
379.3
441.3
491.3
584.3
637.5
721.6
742.5
841.6
887.6
950.7
1063.9
1150.4
1195.7
1243.4
1311.9
1432.2
1492.3
1566.2
1604.5
1718.5
1804.0
1909.0
1953.2

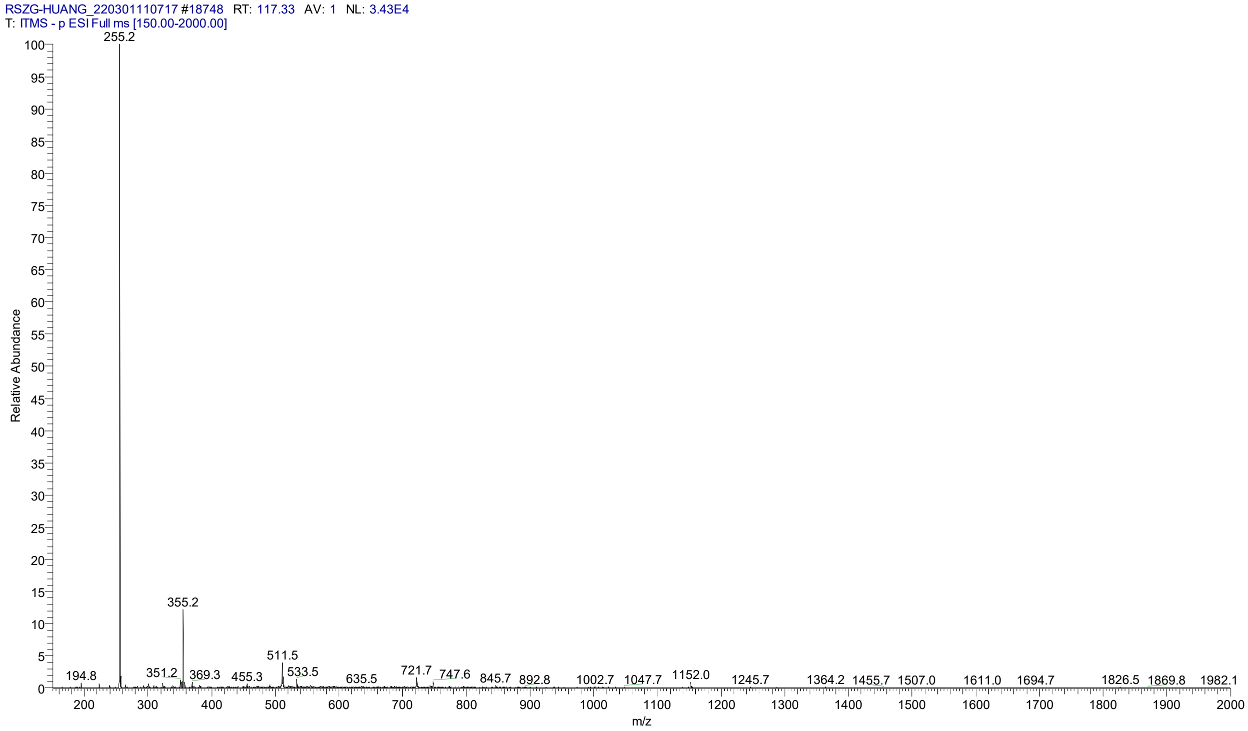
RSZG-HUANG_220301110717 #18748 RT: 117.33 AV: 1 NL: 3.43E4
T: ITMS - p ESI Full ms [150.00-2000.00]
Relative Abundance
m/z
255.2
355.2
511.5
533.5
194.8
351.2
369.3
455.3
635.5
721.7
747.6
845.7
892.8
1002.7
1047.7
1152.0
1245.7
1364.2
1455.7
1507.0
1611.0
1694.7
1826.5
1869.8
1982.1

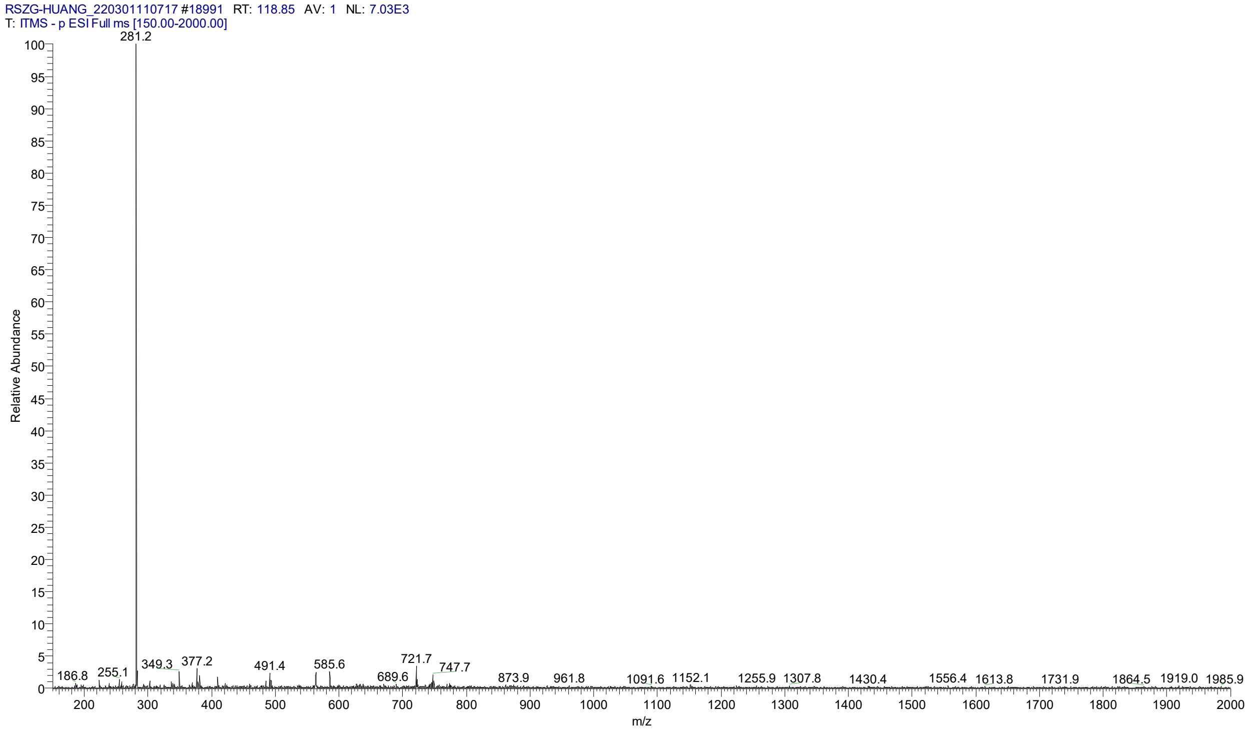
RSZG-HUANG_220301110717 #18991 RT: 118.85 AV: 1 NL: 7.03E3
T: ITMS - p ESI Full ms [150.00-2000.00]
Relative Abundance
m/z
281.2
186.8
255.1
349.3
377.2
491.4
585.6
689.6
721.7
747.7
873.9
961.8
1091.6
1152.1
1255.9
1307.8
1430.4
1556.4
1613.8
1731.9
1864.5
1919.0
1985.9
